# Detectability of radiation-induced changes in magnetic resonance biomarkers following stereotactic radiosurgery: A pilot study

**DOI:** 10.1371/journal.pone.0207933

**Published:** 2018-11-26

**Authors:** Jeff D. Winter, Fabio Y. Moraes, Caroline Chung, Catherine Coolens

**Affiliations:** 1 Radiation Medicine Program, Princess Margaret Cancer Center and University Health Network, Toronto, Ontario, Canada; 2 Department of Radiation Oncology, University of Texas MD Anderson Cancer Center, Houston, Texas, United States of America; 3 TECHNA Institute, University Health Network, Toronto, Ontario, Canada; 4 Department of Radiation Oncology, University of Toronto, Toronto, Ontario, Canada; 5 Institute of Biomaterials and Biomedical Engineering, University of Toronto, Ontario, Canada; North Shore Long Island Jewish Health System, UNITED STATES

## Abstract

Our objective was to investigate direct voxel-wise relationship between dose and early MR biomarker changes both within and in the high-dose region surrounding brain metastases following stereotactic radiosurgery (SRS). Specifically, we examined the apparent diffusion coefficient (ADC) from diffusion-weighted imaging and the contrast transfer coefficient (K^trans^) and volume of extracellular extravascular space (v_e_) derived from dynamic contrast-enhanced (DCE) MRI data. We investigated 29 brain metastases in 18 patients using 3 T MRI to collect imaging data at day 0, day 3 and day 20 following SRS. The ADC maps were generated by the scanner and K^trans^ and v_e_ maps were generated using in-house software for dynamic tracer-kinetic analysis. To enable spatially-correlated voxel-wise analysis, we developed a registration pipeline to register all ADC, K^trans^ and v_e_ maps to the planning MRI scan. To interrogate longitudinal changes, we computed absolute ΔADC, ΔK^trans^ and Δv_e_ for day 3 and 20 post-SRS relative to day 0. We performed a Kruskall-Wallice test on each biomarker between time points and investigated dose correlations within the gross tumour volume (GTV) and surrounding high dose region > 12 Gy via Spearman’s rho. Only v_e_ exhibited significant differences between day 0 and 20 (p < 0.005) and day 3 and 20 (p < 0.05) within the GTV following SRS. Strongest dose correlations were observed for ADC within the GTV (rho = 0.17 to 0.20) and weak correlations were observed for ADC and K^trans^ in the surrounding > 12 Gy region. Both ΔK^trans^ and Δv_e_ showed a trend with dose at day 20 within the GTV and > 12 Gy region (rho = -0.04 to -0.16). Weak dose-related decreases in K^trans^ and v_e_ within the GTV and high dose region at day 20 most likely reflect underlying vascular responses to radiation. Our study also provides a voxel-wise analysis schema for future MR biomarker studies with the goal of elucidating surrogates for radionecrosis.

## Introduction

Stereotactic radiosurgery (SRS) is a well established treatment for patients with brain metastases [1]. Patients with brain metastases typically present with 1–4 lesions, and many exhibit hallmarks of oligiometastatic disease. Utilizing SRS to ablate these brain lesions in patients with well-controlled primary disease and further systemic therapy options has the potential to improve patient-related outcomes, including overall survival (OS) [2]. Recent evidence suggests that SRS alone reduces cognitive deterioration at 3 months without significant differences in OS compared with whole brain irradiation [3]. With ablative SRS dose prescriptions; local target control (LC) must be balanced with the potential for radiation-induced necrosis (radionecrosis). Distinguishing local tumour progression from radionecrosis is a key challenge for evaluation and clinical management of patients with treated brain metastases [4]. Although biopsy following SRS provides histologic confirmation of radionecrosis, it is invasive, only applicable to accessible lesions, and has inherent surgical morbidities [5].

Use of MR biomarkers has the potential to non-invasively characterize dose-related changes within the tumour and surrounding tissue following SRS [6–12]. Early physiological indicators may help to personalize dose prescriptions and assist on better defining clinical endpoints (e.g. LC). A previous report demonstrated that apparent diffusion coefficient (ADC) values generated from diffusion-weighted images (DWI) and a permeability metric from dynamic susceptibility-contrast imaging (DSC) were able to distinguish between responders and non-responders [7,12]. Since radiation dose is a potential determinant for both LC and radionecrosis, it is critical to determine how MR biomarker changes correlate with radiation dose [13].

Understanding the relationship between SRS dose and MR biomarkers changes will enable development of strategies for personalized assessment of treated brain lesions. Utilization of MR biomarkers may help us better delineate treatment response in terms of LC, edema [14], subclinical normal brain injury and most importantly potential radionecrosis. Individual measurements of MR biomarkers as well as longitudinal changes in these parameters has potential to elucidate underlying biophysical changes occurring post-SRS. We hypothesized that dose-related physiological changes following SRS would be detectable using MR biomarkers.

Here we investigate the detectability of voxel-wise dose-correlated changes in MR biomarkers at discrete early time points following SRS treatment of 1–4 brain lesions within the spatially co-registered gross tumour volume (GTV) and the surrounding high-dose normal tissue region. We also investigated impact of tissue edema, identified as hyperintense signal observed on T_2_-weighted MR images on dose-correlated changes in the MRI biomarkers.

## Methods

### Patients

Patients we included in this study were enrolled in one of two clinical trials investigating multi-parametric imaging biomarkers at our institution UNH Research Ethics Board #10-0743-C and 09-0115-C. Both studies were approved by our institution’s Research Ethics Board and all patients provided written consent to participate. Patient inclusion criteria included: age ≥ 18 years old, biopsy proven malignancy of at least one lesion of diameter ≥ 1 cm, life expectancy > 3 months and no anti-cancer therapy or WBRT within 3 days of SRS. We excluded patients with previous cranial radiation < 6 months prior to SRS or previous SRS to the index lesion as well as any counter-indications for MRI scanning. Presence of radionecrosis was assessed via classic morphological changes on consecutive serial post-SRS MRI images for each treated lesion. As this is a prospective phase I pilot study, sample size is by convenience.

### RT Planning and delivery

All patient SRS treatments were planned with GammaPlan V10 software (Elekta, Sweden) and delivered as single fraction SRS on the Gamma Knife Perfexion platform (Elekta, Sweden). Targets were delineated on T_1_-weighted images and prescribed radiation dose between 15–21 Gy in a single fraction (following institutional guidelines). We exported dose and contours in the post-contrast T_1_-weighted image volume coordinate system.

### MR imaging

We collected MRI data on a 3 Tesla Siemens Verio scanner (Siemens Medical Systems, Erlangen, Germany) at day 0, day 3 and day 20 post-SRS. Initial imaging consisted of either a T_2_-weighted or fluid-attenuated inversion recovery (FLAIR) acquisition: echo time (TE) = 96 ms, repetition time (TR) = 7253 ms, field-of-view (FOV) = 220 x 220 mm, matrix = 320 x 320, slice thickness (SL_TH_) = 3mm, and number of slices (N_SL_) = 50. Next, we collected DWI scans using single shot echo planar imaging: TR = 7700, TE = 110 ms, FOV = 200 x 200 mm, matrix size = 128 x 128, number of averages = 3, SL_TH_ = 3 mm, N_SL_ = 50, and b-values = 0, 300, 1000, 1800 s/mm^2^ and used the ADC maps generated by the scanner in our analysis. DCE-MRI data were collected using a 3D spoiled gradient echo sequence: TR = 4.7 or 4.8 ms, TE = 1.49 or 1.86 ms, flip angle (FA) = 10 or 20°, FOV = 200 x 220 mm, matrix = 174 x 192, SL_TH_ = 1.5 mm, N_SL_ = 22 or 40, and temporal resolution = 5.8 s. Prior to contrast injection, we collected data for a T_1_-map using the variable flip angle approach with flip angles of 2, 10, 20, and 30° using the same DCE MRI imaging parameters. We injected an intravenous contrast bolus of 0.2 mmol/kg Gadolinium-diethylenetriamine pentaacetic acid at 4 mL/s after 20 seconds of DCE image acquisition followed by 20 mL saline. Finally, we collected contrast-enhanced T_1_-weighted 3D magnetization-prepared rapid gradient-echo images: TE = 2.2 ms, TR = 1400 ms, inversion time = 900 ms, FOV = 200 x 200 mm, matrix = 320 x 320, SL_TH_ = 1.5 mm; and N_SL_ ≈ 100.

### DCE-MRI Analysis

For quantification of DCE-MRI parameters, we employed the 4-dimensional temporal dynamic analysis (TDA) approach [15] that we recently validated against DCE-CT in brain metastases [16]. Our TDA approach classifies voxels based on contrast-enhancement dynamics and applies specific kinetic model fitting based on each classification. The TDA algorithm then iteratively updates parameter maps and voxel classification to generate robust estimates of DCE-MRI parameter maps. Using TDA, we quantified DCE-MRI tissue contrast uptake using the modified Tofts model [17]:

$$C_{t}(t)=\frac{K^{trans}}{1-Hct}(C_{a}(t)⊗e^{{-k}_{ep}(t-\tau )})+V_{b}C_{a}(t)$$ (1)

where *C*_*t*_(*t*) is the tissue contrast concentration, *C*_*a*_(*t*) is the arterial input function (AIF), *K*^*trans*^ is the transfer constant from blood plasma to the extracellular extravascular space (EES), *k*_*ep*_ is the transfer constant from the EES to blood plasma, *V*_*b*_ is the blood volume per unit tissue (ml / g), *Hct* is the hematocrit (assumed to be 0.4 for all patients). We employed a population-averaged AIF to estimate *C*_*a*_(*t*). To obtain *C*_*t*_(*t*) values for each voxel, we used T_1_ maps wherever possible or assumed baseline tumour T_1_ of 1700 ms, the spoiled gradient echo signal equation and the linear relationship between ΔR_1_ and contrast agent concentration. We also computed the volume of EES (*ve*) as the ratio *K*^*trans*^/*k*_*ep*_. We performed voxel-wise fitting of DCE-MRI data following motion-compensatory image registration of the dynamic DCE-MRI data.

### Image registration

To enable spatially registered voxel-wise analyses, we developed a rigorous in-house registration pipeline to perform all image registration steps as well as visualize image registration results using Python (Python Software Foundation, https://www.python.org/) interacting with 3D Slicer [18]. Our first step in the imaging pipeline was to generate semi-automated masks for the non-brain regions, which we used for all registration steps. Next, we co-registered all ADC and DCE image volumes to the post-contrast T_1_-weighted volume for each imaging session. For the ADC registration, we first registered T_2_-weighted or FLAIR volumes to the post-contrast T_1_-weighted volume via rigid-body registration followed by registration of b = 0 s/mm^2^ DWI volume to the T2-weighted or FLAIR volume via B-Spline deformable registration. We then registered DCE parameter map volumes (K^trans^, k_ep_) to the post-contrast T_1_-weighted via rigid-body registration of the first volume in the dynamic series. Once all intra-session registrations were complete, we registered all session images to the planning T_1_-weighted image. Following these registration steps, all MR volume data were in the common coordinate system of the planning MR volume, including radiation therapy dose and structure sets.

### Image analysis

Following image registration, we used the clinical GTV contour to extract dose, ADC, K^trans^, and v_e_ data from within each of the imaged brain metastases at each time point. From each GTV, we extracted the mean MR biomarker value for each of the following dose levels: 12–18 Gy, 18–24 Gy, 24–30 Gy, and > 30 Gy. We also extracted ADC, K^trans^ and v_e_ values from the high dose region surrounding each GTV (> 12 Gy). We selected the threshold of 12 Gy because the volume receiving > 12 Gy has been previously linked to radionecrosis [19]. We calculated ΔADC, ΔK^trans^, and Δv_e_ values for day 3 and 20 as the absolute change in each parameter relative to day 0. For each patient, we also manually segmented regions of T_2_ signal hyperintensity within the volume receiving > 12 Gy to investigate the influence of edema on dose correlations with MR biomarkers. We reviewed each registration step to ensure accuracy. Moreover, we created a contralateral control ROI of approximately the same volume as each lesion to investigate potential systematic variations in the MR biomarkers using an unpaired Kruskall-Wallis H-test for ΔADC, ΔK^trans^, and Δv_e_ using the null hypothesis of zero.

### Statistics

We investigated between-day ADC and K^trans^ differences using the non-parametric Kruskall-Wallis H-test for both within the GTV as well as within the > 12 Gy dose region. We also used the Kruskall-Wallis H-test to examine significant differences between different dose ranges within the GTV, including 12–18, 18–24, 24–30, and > 30 Gy. Any significant differences in the omnibus tests were investigated with non-parametric paired Kruskall-Wallis H-tests. To assess the relationship between dose and MR biomarkers, we performed a series of linear regression tests at each time point in the GTV and > 12 Gy dose regions to determine the slope of the dose versus MR biomarker parameter plot:

- global ADC and K^trans^, v_e_, ΔADC and ΔK^trans^ Δv_e_ versus dose;
- ΔADC and ΔK^trans^ Δv_e_ versus dose for the radionecrotic lesions only; and,
- ADC and K^trans^, v_e_, ΔADC and ΔK^trans^ Δv_e_ versus dose for each metastasis;

To assess the strength for each correlation we performed a Spearman's rank correlation coefficient test. The large number of voxels included in the regression makes the use of the p-value for identifying significance challenging as all voxels within the same metastasis represents a repeated measure. However, each voxel also has potential to exhibit independent characteristics, including perfusion, vascularity, oxygenation and cellularity and radiation dose level. For this reason we did not use a mixed model regression and have plotted all individual lesion data in the Supplemental information (S1 Fig). We also investigated ΔADC, ΔK^trans^, and Δv_e_ relationships with dose in the > 12 Gy high dose region in regions with and without edema.

## Results

### Mean biomarker changes

**Fig 1:**
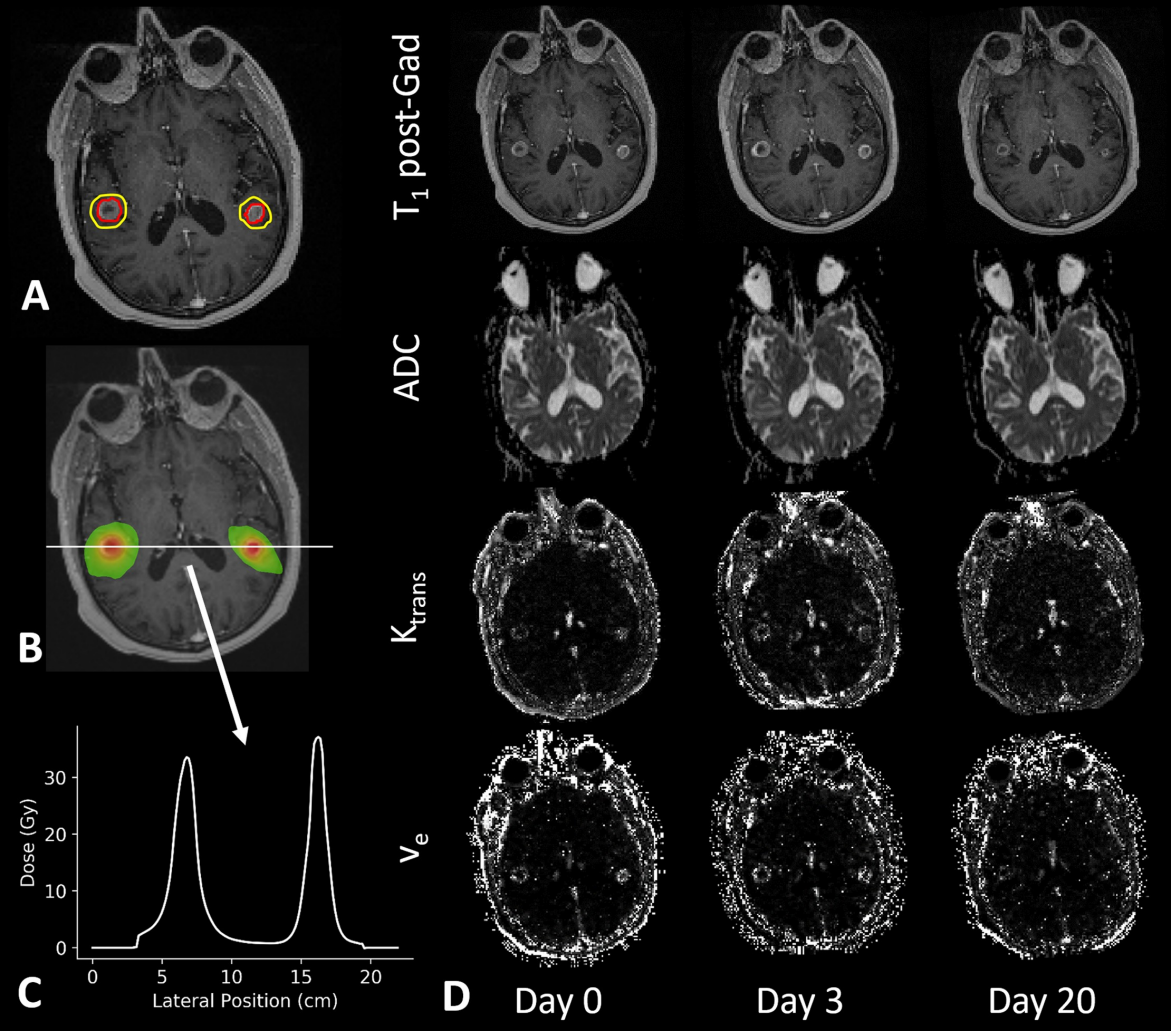
Representative patient with (A) GTV (red) and > 12 Gy high-dose (yellow) contours, (B) dose map and (C) corresponding dose profile through the two lesions. In panel (D) ADC, K^trans^ and v_e_ maps are illustrated at day 0, day 3 and day 20 post-SRS.

**Fig 2:**
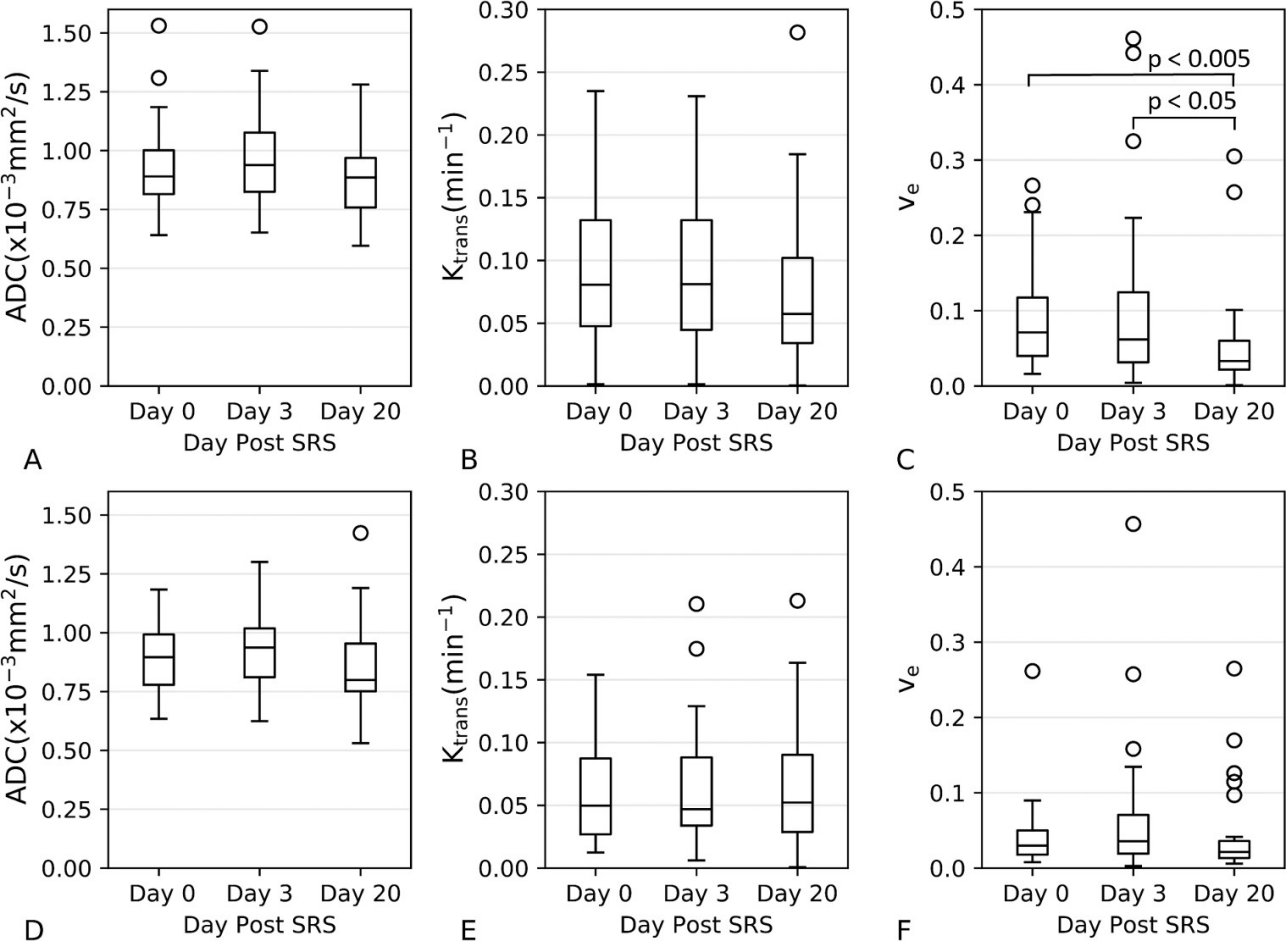
Mean ADC, K^trans^ and v_e_ values at each time point for all patients in the GTV (A—C) and non-target region > 12 Gy (D–F).

**Fig 3:**
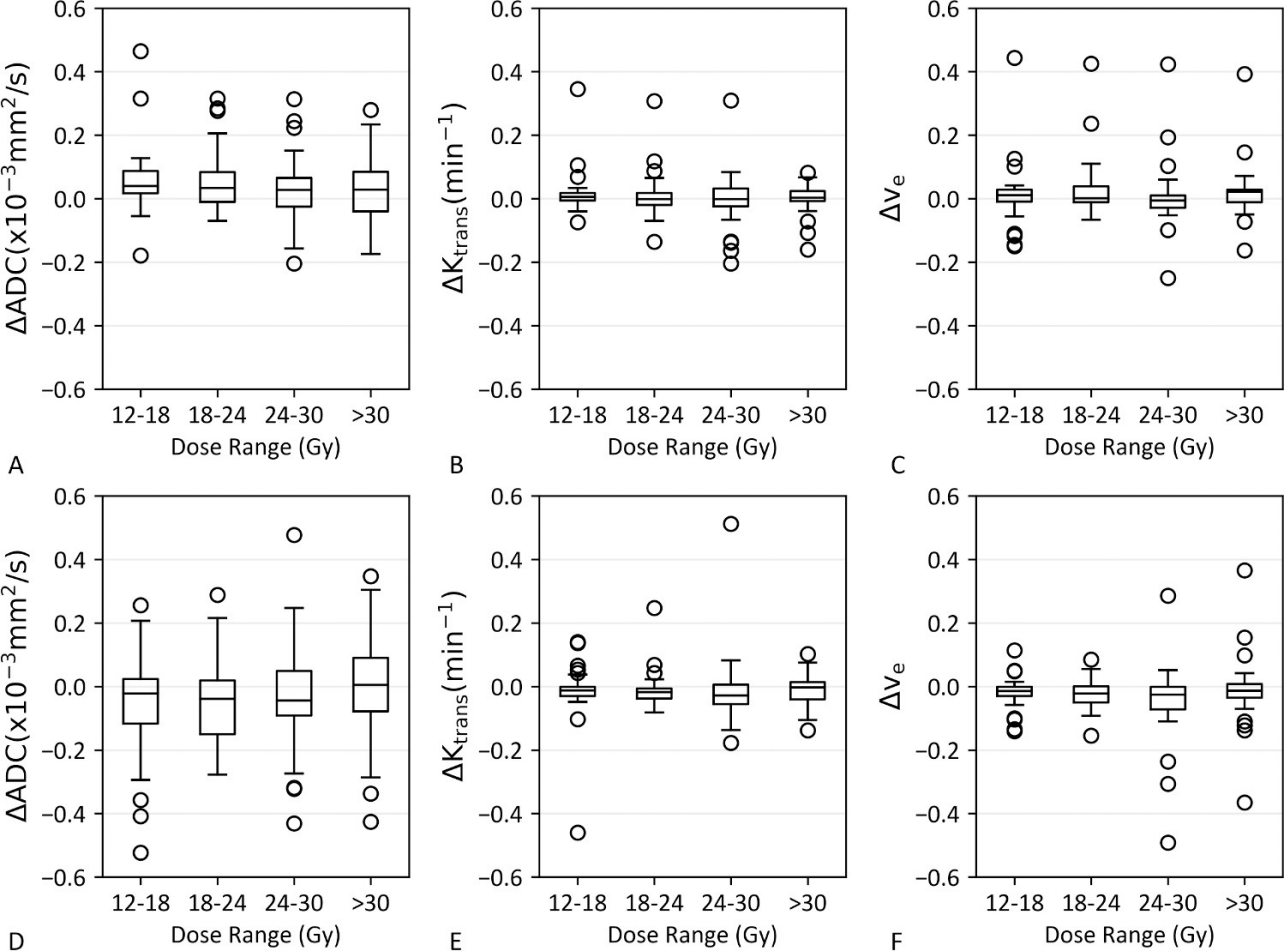
Mean ΔADC, ΔK^trans^ and Δv_e_ values for various dose ranges within the GTV at day 3 (A—C), and day 20 (D–F).

Table 1 provides characteristics of all 18 patients included in this study. To note, patients 14 and 15 had only one of the lesions within the slice range of the DWI and DCE-MRI acquisitions. Four patients had a single lesion with evidence of radionecrosis, and one patient had two lesions with radionecrosis, only one of which was within the imaging slice coverage. Day 3 imaging was not available for Patients 1 and 10. Both GTV contours and dose > 12 Gy regions corresponding to each of the metastases for a representative patient are illustrated in Fig 1. In Fig 1 (panel D), the T_1_ contrast-enhanced images as well as ADC, K^trans^ and v_e_ maps for the same representative patient are displayed. Note decreased contrast enhancement at day 20 on T_1_ post-contrast images corresponds to decreased K^trans^ and v_e_. In Fig 2, mean ADC, K^trans^ and v_e_ values are plotted for each time point for GTV and > 12 Gy dose regions. Histograms for each MR biomarker for each lesion are supplied in S1 Fig. The Kruskall-Wallis test was only significant for v_e_ within the GTV (p < 0.05). Post-hoc Kruskall-Wallis tests for v_e_ within the GTV showed a significant decrease in v_e_ at day 20 compared with day 0 (p <0.005) as well as day 20 compared with day 3 (p < 0.05). Spaghetti plots demonstrating changes between time points on a per metastasis basis are provided in S2 Fig. Comparing the ΔADC, ΔK^trans^, and Δv_e_ values between the four dose bins (12–18, 18–24, 24–30, and > 30 Gy) within the GTV revealed no significant differences between the dose bins at either day 3 or day 20 (Fig 3).

**Table 1:** Patient characteristics.

Patient	N of Mets treated	Sex (M/F)	Age at SRS (years)	Tumor histology	Steroid Dose (mg)	Steroid Duration (days)	Tumour response	WBRT (Y/N)	RN Occurrence (Y/N)
**1**	1	F	78	non-small cell lung	0	0	—	N	Y
**2**	2	F	43	cervix squamous cell	0	0	Partial	N	N
**3**	1	M	76	non-small cell lung	0	0	Progression	Y	N
**4**	2	F	50	breast	0	0	CR	N	N
**5**	4	M	74	renal cell carcinoma	8	14	PR	N	N
**6**	1	F	60	non-small cell lung	0	0	PR	Y	N
**7**	1	F	70	non-small cell lung	0	0	PR	N	N
**8**	1	F	51	head and neck	16	7	PR	Y	N
**9**	1	F	63	renal cell carcinoma	2	14	CR	N	N
**10**	1	F	71	non-small cell lung	0	0	—	N	Y
**11**	3	M	50	non-small cell lung	0	0	—	N	Y
**12**	4	F	66	breast	12	7	—	N	Y
**13**	3	M	62	melanoma	0	0	PR	N	N
**14**	2	M	59	head and neck	8	10	Stable	N	N
**15**	2	M	50	melanoma	16	14	Stable	N	N
**16**	2	F	46	breast	0	0	Stable	N	N
**17**	2	F	44	breast	0	0	—	N	Y
**18**	1	M	63	non-small cell lung	0	0	Progression	N	N

Abbreviations: N = number, Mets = metastases, F = female, L = left, M = male, RN = radionecrosis, WBRT = whole brain radiation therapy, PR = partial response. CR = complete response

### Global dose correlations

Global voxel-wise correlation of MR biomarkers and dose across all patients and all metastases exhibited numerous weak correlations (Table 2). Within the GTV, both ADC and v_e_ values were positively correlated with dose for all days, whereas K^trans^ only correlated with dose at day 0. In the > 12 Gy region, only K^trans^ exhibited correlations with dose at day 0. In the GTV, both ΔK^trans^ and Δv_e_ exhibited a negative correlation with dose at days 3 and 20 post-SRS, which suggests K^trans^ and v_e_ decreases post-SRS are greatest in regions receiving higher dose. Similarly, in the > 12 Gy region, ΔK^trans^ and Δv_e_ exhibited a negative correlation with dose, but only at day 20. No trends towards correlations existed for ΔADC.

**Table 2:** Linear regression results between dose and MR biomarkers for all voxels and all patients.

		GTV		Non-target region > 12 Gy	
Parameter	Day post-RT	Slope	Spearman’s rho	Slope	Spearman’s rho
ADC	0	0.0147	0.19	0.0030	0.02
	3	0.0135	0.19	0.0028	0.02
	20	0.0138	0.17	0.0004	0.00
K^trans^	0	0.0009	0.04	0.0013	0.03
	3	-0.0001	-0.00	0.0010	0.02
	20	0.0001	0.00	-0.0007	-0.02
v_e_	0	0.0050	0.16	-0.0001	-0.00
	3	0.0041	0.11	0.0004	0.01
	20	0.0012	0.05	-0.0004	-0.01
ΔADC	3	0.0010	0.02	0.0014	0.01
	20	-0.0006	-0.01	-0.0014	-0.01
ΔK^trans^	3	-0.0008	-0.03	-0.0007	-0.02
	20	-0.0014	-0.05	-0.0027	-0.07
Δv_e_	3	-0.0013	-0.04	0.0003	0.01
	20	-0.0057	-0.16	-0.0017	-0.04

All values are ± standard deviation. Slope units are ×10^−3^ mm^2^/s/Gy for ADC, min^-1^/Gy for K^trans^ and /Gy for v_e_.

### Dose correlations in radionecrotic lesions

Unlike the global correlations for all lesions, we found a very weak dose-correlated ΔADC increase at both day 3 and 20 in the GTV. Results for day 3 slope = 0.006×10^−3^ mm^2^/s/Gy with rho = 0.21 and day 20 slope = 0.0072×10^−3^ mm^2^/s/Gy with rho = 0.11. The only other significant correlation was for v_e_ at day 20, with slope = -0.0015 Gy^-1^ with rho = -0.12.

### Dose correlations per metastasis

For the relationship between dose and the MR biomarkers per individual metastasis, multiple correlations for ADC, K^trans^ and v_e_ existed, although considerable variability existed in slope direction. Mean results for all metastases exhibiting correlations in both GTV region >12 Gy dose regions are summarized in Table 3. As expected, we found that the general direction of the biomarker-to-dose relationship was consistent with the results found in the global analysis for biomarkers with significant global correlations. For example, the mean slope values for ΔK^trans^ and Δv_e_ in Table 3 for the GTV were negative at both days 3 and 20, which matches the corresponding values in Table 2.

**Table 3:** Linear regression results between dose and MR biomarkers averaged over metastases with significant correlations. rho = Spearman’s rho.

		GTV		Non-target region > 12 Gy	
Parameter	Day post-RT	Mean Slope	Mean rho	Mean Slope	Mean rho
ADC	0	0.0074 ± 0.0351	0.01 ± 0.44	0.0042 ± 0.0166	0.09 ± 0.17
	3	0.0018 ± 0.0294	-0.05 ± 0.44	0.0053 ± 0.0142	0.06 ± 0.14
	20	0.0148 ± 0.0309	0.16 ± 0.32	0.0056 ± 0.0114	0.05 ± 0.09
K^trans^	0	0.0026 ± 0.0068	0.13 ± 0.30	0.0026 ± 0.0041	0.17 ± 0.15
	3	0.0005 ± 0.0061	0.17 ± 0.30	0.0033 ± 0.0044	0.16 ± 0.14
	20	0.0045 ± 0.0067	0.24 ± 0.23	-0.0017 ± 0.0090	0.06 ± 0.17
v_e_	0	0.0059 ± 0.0093	0.33 ± 0.23	0.0028 ± 0.0037	0.24 ± 0.14
	3	0.0045 ± 0.0096	0.34 ± 0.33	0.0066 ± 0.0155	0.27 ± 0.23
	20	0.0042 ± 0.0049	0.36 ± 0.23	0.0029 ± 0.0057	0.16 ± 0.17
ΔADC	3	-0.0021 ± 0.0100	-0.04 ± 0.32	0.0018 ± 0.0190	-0.01 ± 0.19
	20	0.0080 ± 0.0174	0.14 ± 0.30	-0.0007 ± 0.0214	-0.06 ± 0.13
ΔK^trans^	3	-0.0042 ± 0.0068	-0.06 ± 0.27	0.0000 ± 0.0059	0.05 ± 0.13
	20	0.0005 ± 0.0105	-0.02 ± 0.34	-0.0062 ± 0.0077	-0.12 ± 0.11
Δv_e_	3	-0.0030 ± 0.0192	-0.06 ± 0.36	0.0141 ± 0.0475	0.07 ± 0.31
	20	-0.0051 ± 0.0127	-0.25 ± 0.35	-0.0025 ± 0.0058	-0.18 ± 0.14

### Impact of edema

**Fig 4:**
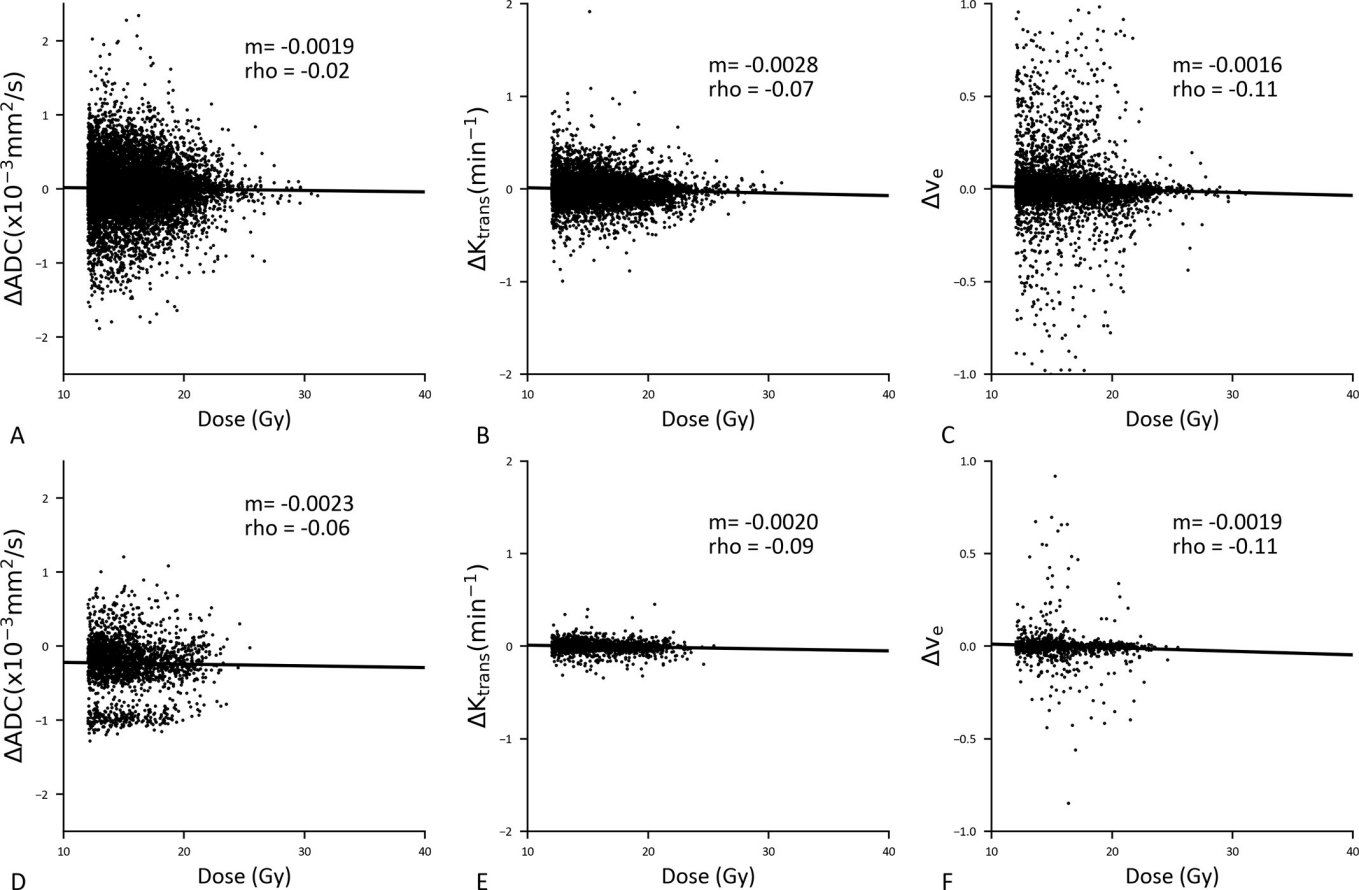
Relationship between dose and ΔADC, ΔK^trans^, and Δv_e_ for the > 12 Gy dose region without edema (A–C) and with edema (D-F) at day 20 post-SRS. m = slope, rho = Spearman’s Rho, p = p value.

Correlation results for ΔADC, ΔK^trans^ and Δv_e_ at day 20 within and outside regions of suspected edema in the dose > 12 Gy region are displayed in Fig 4. No correlations between ΔADC and dose existed within the region of edema. However, for ΔK^trans^, correlations were exhibited with dose both within and outside regions of suspected edema, with similar magnitude of slope. Negative correlations were observed for Δv_e_ at day 20 in both regions with and without suspected edema, with similar slope values as well.

### Control ROI verification

**Fig 5:**
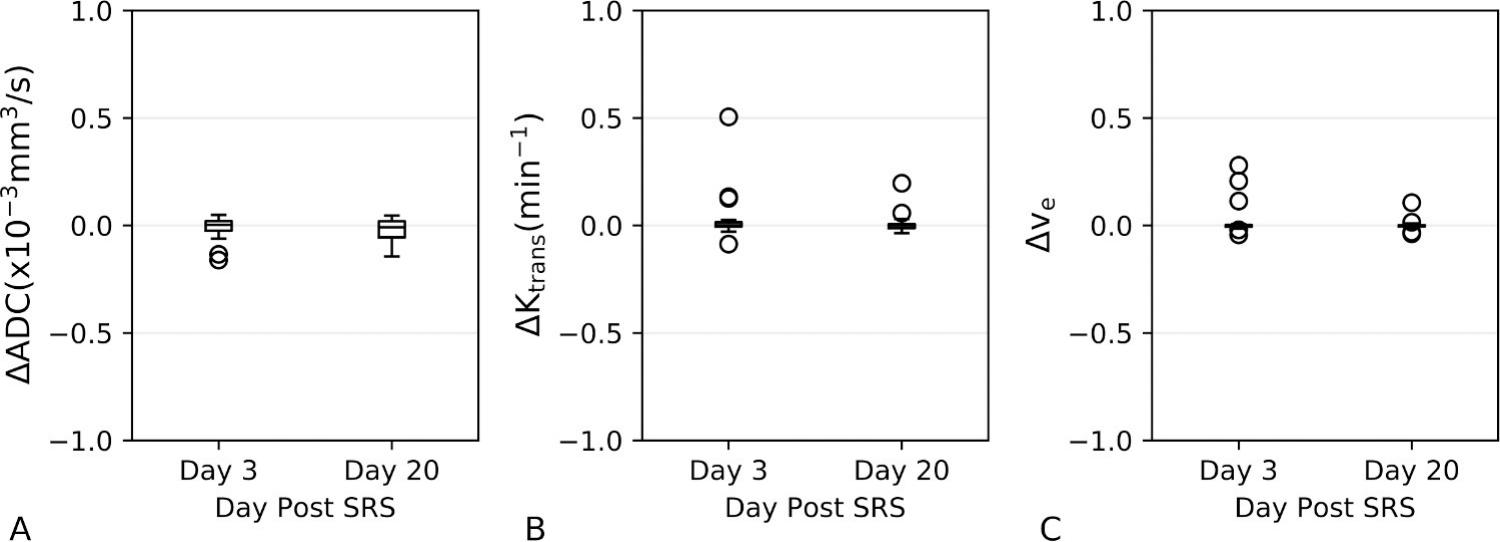
Mean ΔADC (A), ΔK^trans^ (B) and Δv_e_ (C) values for various dose ranges within the control ROIs used to assess potential systematic errors related to the image registration.

Results of the control ROI analysis for ΔADC, ΔK^trans^, Δv_e_ revealed no significant changes from the null hypothesis of 0 for both day 3 and 20 (Fig 5).

## Discussion

Imaging biomarkers hold promise for the non-invasive interrogation of physiological changes within brain metastases as well as the surrounding tissue at early time points following SRS with the goal of identifying surrogates of radionecrosis caused by radiation-induced vascular injury and necrosis. Magnetic resonance imaging offers the ability to interrogate the early changes in vascular impairment using DCE-MRI and microstructural cellular changes using diffusion imaging. Our primary goal was to assess detectability of dose-correlated MR biomarker changes within and surrounding brain metastases to improve our understanding of how functional changes can be characterized. Here we provide the results from the first combined report of early voxel-wise changes in diffusion and DCE-MRI within the GTV and surrounding high dose region following GammKnife SRS, including dose correlations and potential impact of edema.

Our results indicate that in general early changes in K^trans^ and v_e_ are very weakly dependent on dose, with the most important observation being dose-correlated decreases in K^trans^ and v_e_ within the GTV and > 12 Gy region at day 20. This is potentially related to the fact that delivery of ablative SRS doses to brain metastases drives vascular injury and anti-angiogenic response. Previous work showed that a DCE-MRI permeability decrease within brain metastases at 1 week post-SRS was predictive of treatment response [7], although differences in permeability quantification make direct comparisons with the cited study challenging. Another study, investigating predictive value of DCE-MRI metrics < 12 weeks post-SRS, demonstrated decreased K^trans^ and v_e_ measures post-SRS in non-progressing metastases, although their key result was demonstrating predictive value of post-contrast K^trans^ standard deviation for distinguishing progressing versus non-progressing metastases [20]. Both increases [21] and decreases [7] in DSC measures of rCBV have been observed in progressive disease versus non-progressive disease after 4–6 weeks post-RT. Given differences in timing and an unclear relationship between v_e_ and rCBV, once again direct comparison of our results is not possible.

Our results showed ADC correlations with dose within the GTV, but we did not observe differences at days 3 and 20 post-SRS time. Following SRS, we expect cytotoxic edema at early time point to restrict water diffusion with later ADC increases resulting from cellular membrane disruption and necrosis. Our lack of ADC changes agrees with one previous study that did not observe changes in responders at one week post-SRS [12] (given that most metastases in our study responded to SRS). Our results also agree with a recent study from Chen *et al*. that failed to show mean ADC changes until 6 months post-SRS [8]. Only one study has shown significant ADC increases at 7 days post-SRS, with a trend for increasing ADC out to 20 months [10]. Despite a limited number of 5 lesions with presence of radionecrosis, our result suggests ADC could identify lesions that will develop radionecrosis.

Magnetic resonance biomarkers may offer means to interrogate established biological processes in the normal tissues surrounding cerebral neoplasms, including endothelial apoptosis, vascular impairment and later vascular renormalization. Previously Jakubovi et al. investigated changes in the ratio of rCBF and rCBV in high dose regions surrounding brain metastasises relative to the contralateral brain region and found significantly increased rCBV in all dose regions > 2 Gy and significantly increased rCBF in the dose region > 10 Gy [6]. We report dose correlations with absolute DCE-MRI parameter changes on a voxel-wise basis to better understand longitudinal changes within each normal tissue voxel. Our results indicate a dose-dependent decrease in K^trans^ and v_e_ at 20 days post-SRS (Table 2). These results are generally supported by a previous investigation of dose-related reductions in rCBV and rCBF in normal tissue within the high-dose region surrounding brain gliomas following conventional RT [22]. However, recent work by Constanzo *et al*. showed that DCE-MRI changes were not significantly different at 54 days post-irradiation of the primary motor cortex of the rat brain following Gamma Knife SRS [23]. The peritumoral normal tissue has also been investigated previously with ADC, specifically investigating the sharpness of the ADC transition from the metastasis to peritumoral tissue[24]. In our study we did not observe significant dose-correlated ADC changes in the > 12 Gy region.

A critical aspect of our analysis was the use of voxel-wise analysis of dose correlations, which spatially links the biomarker changes to the dose delivered to each individual voxel. We observed several dose-correlated changes using linear regression, however we failed to observe significant differences between dose bins within the GTV, possibly attributable to heterogeneity of tumour type and size, which points to the importance of directly tracking MR biomarker changes on a voxel-wise basis. This analysis approach presented challenges in the image registration and involved co-registration of DCE and diffusion volumes to structural T_1_ images as well as registration between time points. We employed deformable image registration to correct moderate diffusion MRI geometric distortions and verified that any residual distortions were away from the region of analysis. We purposely avoided complex image registration tools, particularly those requiring manual intervention, in favour of a robust automated pipeline that could be easily applied clinically.

Our report is one of the first studies to investigate early DCE-MRI imaging parameters changes following RT of brain metastases using well-established modified Tofts model [17] rather than DSC-MRI perfusion. DCE-MRI is particularly well-suited to quantitatively assess permeability of the microvasculature and blood-brain barrier but lack of robust and automated analysis tools limits clinical use. In this study, we employed purpose-built DCE-MRI analysis software using the TDA approach to improve the robustness of DCE-MRI parameter maps by exploiting characteristic temporal dynamics of each tissue type with the brain to improve overall parameter fit quality [15,16]. With improved standardization of protocols, including T1 mapping, and availability of analysis tools DCE-MRI may become a more widely used clinical tool for oncologic imaging and response assessment.

A key objective for MR biomarkers in the assessment of brain metastases is to provide early indication of radionecrosis events. This was not a primary aim for the current study as there were not enough patients and events to provide the statistical power to assess potential value of MR biomarkers in identifying radionecrosis. However, we did find significant direct dose correlations with ΔADC that should be investigated in future with a larger patient group.

A key limitation of this study was the range of metastasis sizes, volume receiving > 12 Gy, as well as primary metastases histology. We know from previous work that diffusion characteristics vary with histology and cellularity, e.g., well-differentiated adenocarcinoma tend to exhibit greater hypo-intensity on diffusion-weighted images [25]. In terms of methodology, another potential limitation was that the superior-inferior position of the metastases varied considerably so it was not possible to consistently capture the vascular input function within the DCE-MRI imaging range to generate a patient-specific AIF. Instead we employed a population-based AIF, which provides a robust and consistent AIF for each patient that is not dependent on slice positioning and subjective extraction of AIF voxels. Although it is possible to employ pre-bolus injection approaches [26] to acquire high-quality patient-specific AIFs, it was not possible to implement due to our limits on overall scan time. Standardization of DCE-MRI and diffusion protocols and analysis tools both within and between institutions is critical to improve our ability to interrogate these parameters as potential clinical biomarkers. Another limitation of the dose correlation results is that in SRS treatments dose is ostensibly falling off as a function of distance from the GTV centroid, so it is difficult to resolve whether dose-correlations are directly related to dose or distance from the center of the lesion.

## Conclusions

Currently there is considerable interest in developing non-invasive tools capable of resolving radionecrosis following SRS. Our salient result was the observed decreases in K^trans^ and v_e_ at day 20 post-SRS, which were correlated with dose both within the GTV and in the surrounding high dose region. We also found that presence of edema had little impact on these correlations in the high dose region. Our results will inform future development and investigation of MR biomarkers for brain metastasis response to radiotherapy with the goal of identifying radiation necrosis. We also present a general framework for investigating voxel-wise MR biomarker changes, with the ability to link these changes with dose. We plan to provide further validation of the efficacy of this framework in terms of registration accuracy for future studies.

## Supporting information

S1 Fig Histogram plots for each MR biomarker and for each metastasis. (DOCX)

S2 Fig Spaghetti plots for MR biomarker changes between Day 0, Day 3 and Day 20. Mean ADC, Ktrans and ve value changes per metastasis at each time point for all patients in the GTV (A—C) and non-target region > 12 Gy (D–F).
(TIF)
