# Supplementary material for: Detectability of radiation-induced changes in magnetic resonance biomarkers following stereotactic radiosurgery: A pilot study
Source: PLoS One. 2018 Nov 26;13(11):e0207933. doi: 10.1371/journal.pone.0207933 (PMC6258119; doi:10.1371/journal.pone.0207933)
Supplement: S1 Fig — (DOCX) [file pone.0207933.s001.docx]

**Patient 1: Metastasis 1
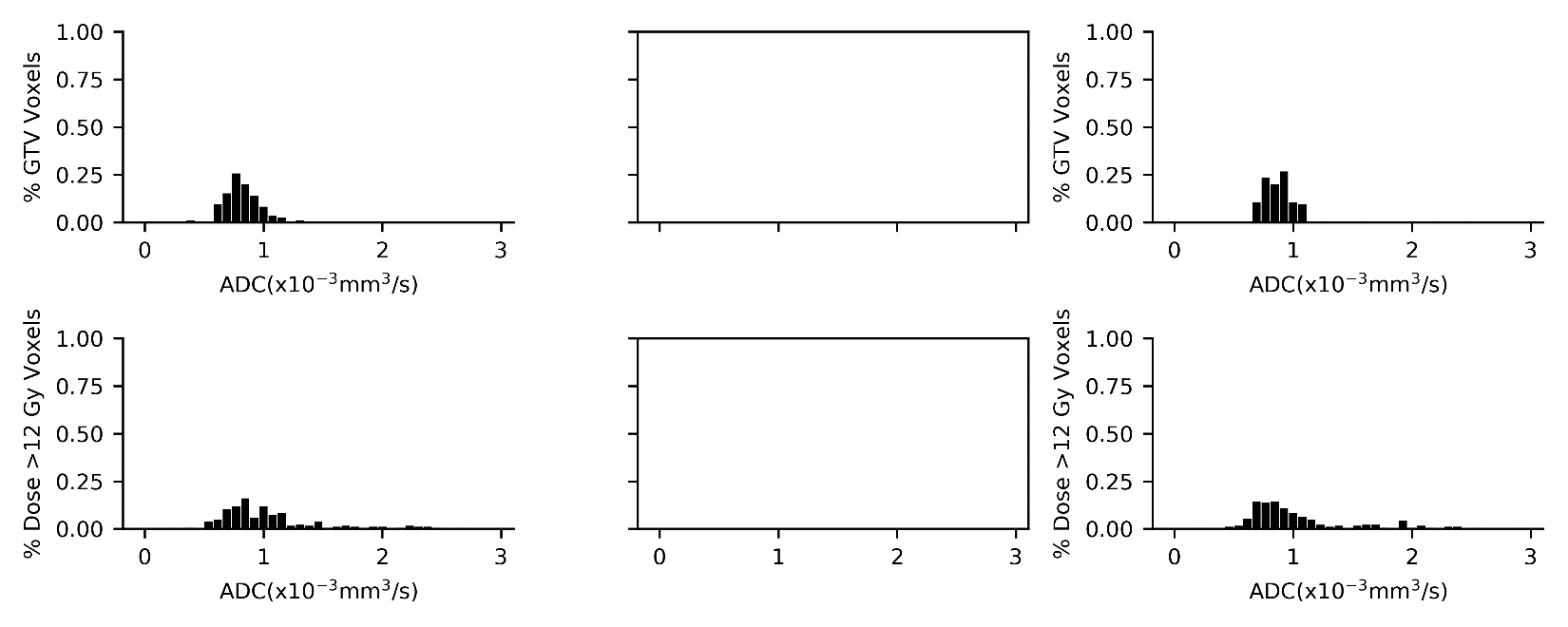
**

**
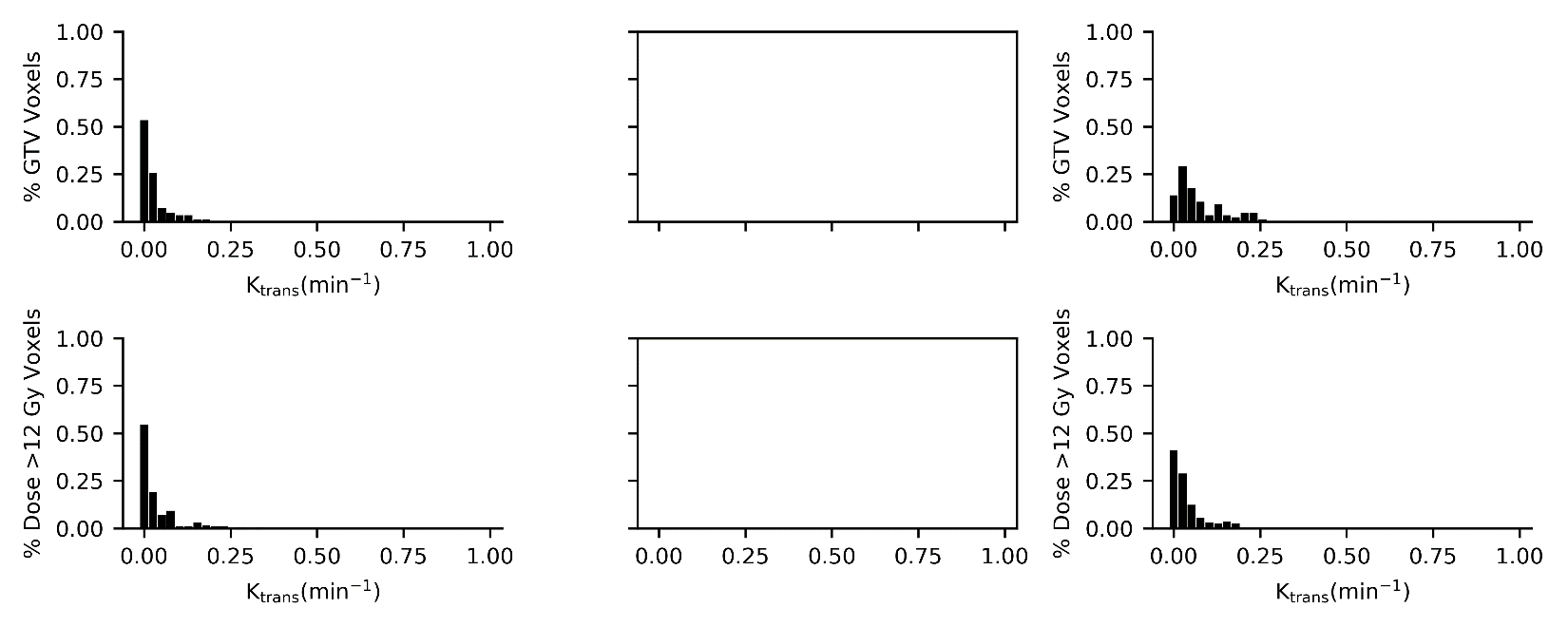
**

**
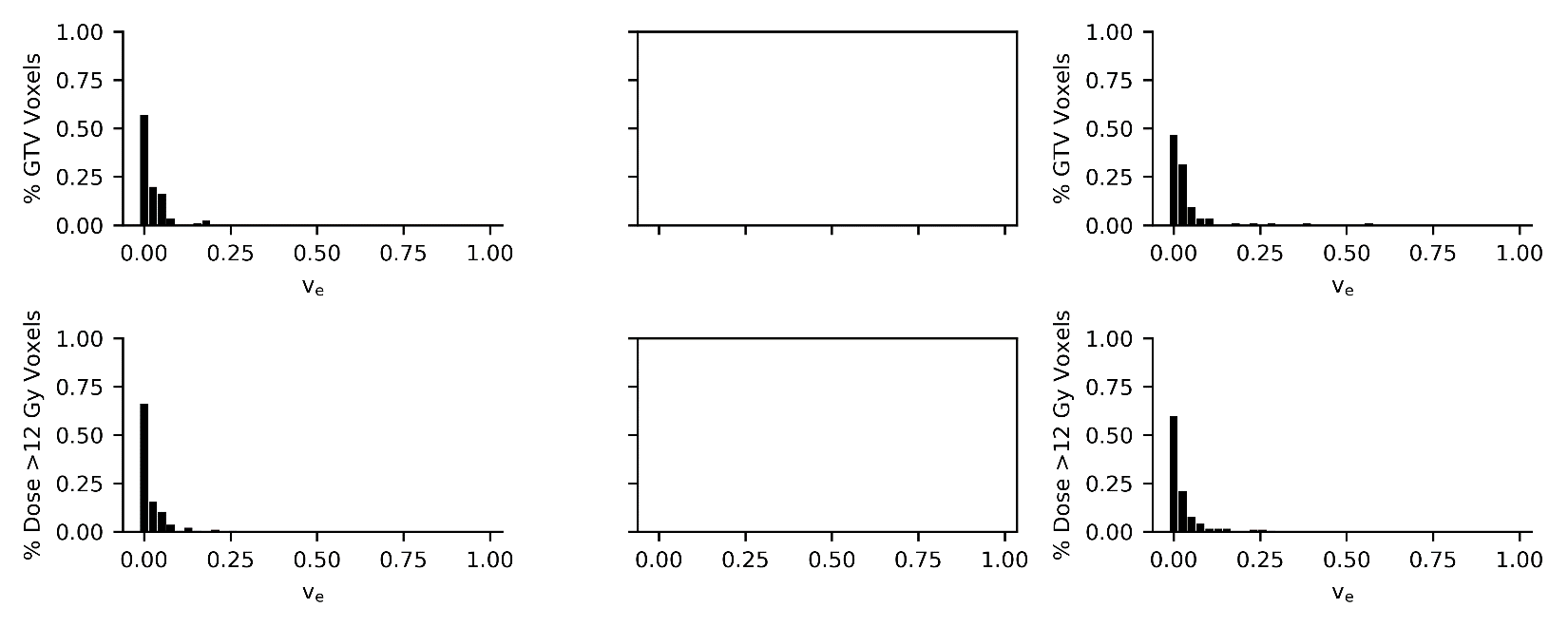
**

**Day 0 Day 3 Day 20**

**Patient 2: Metastasis 1
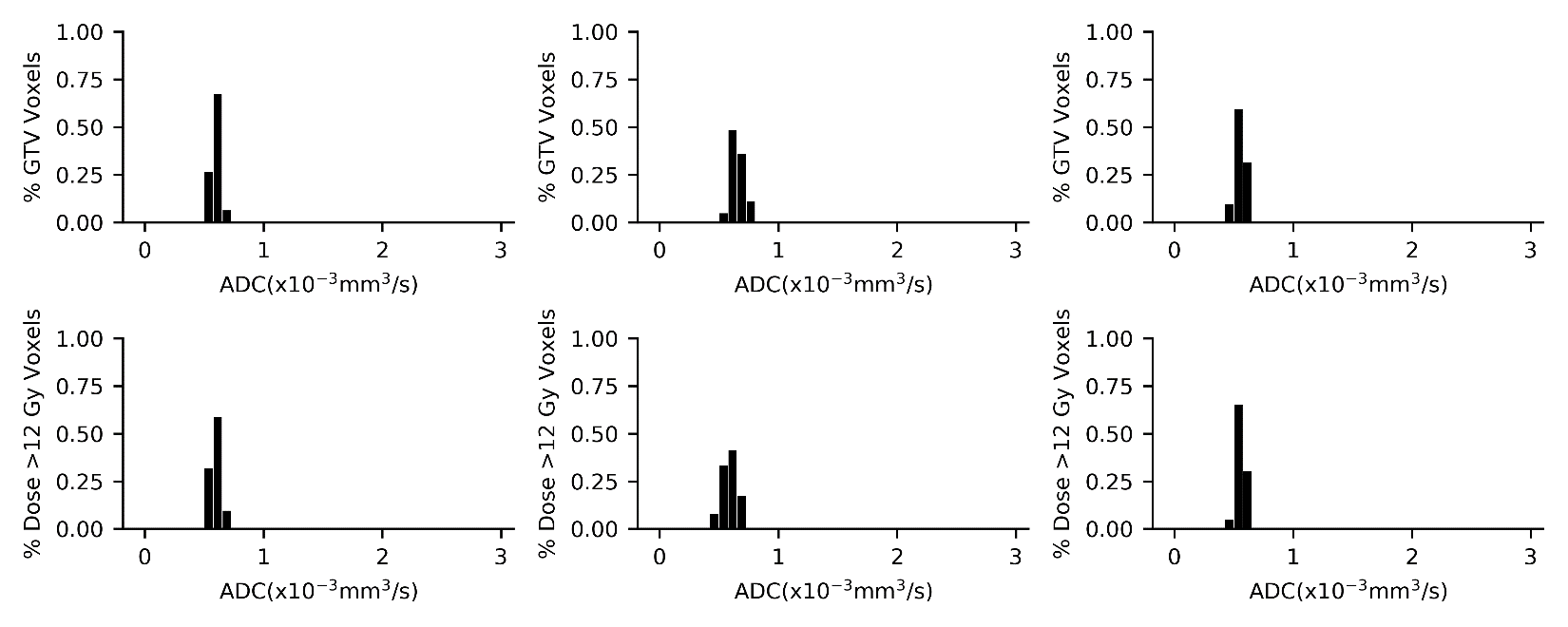
**

**
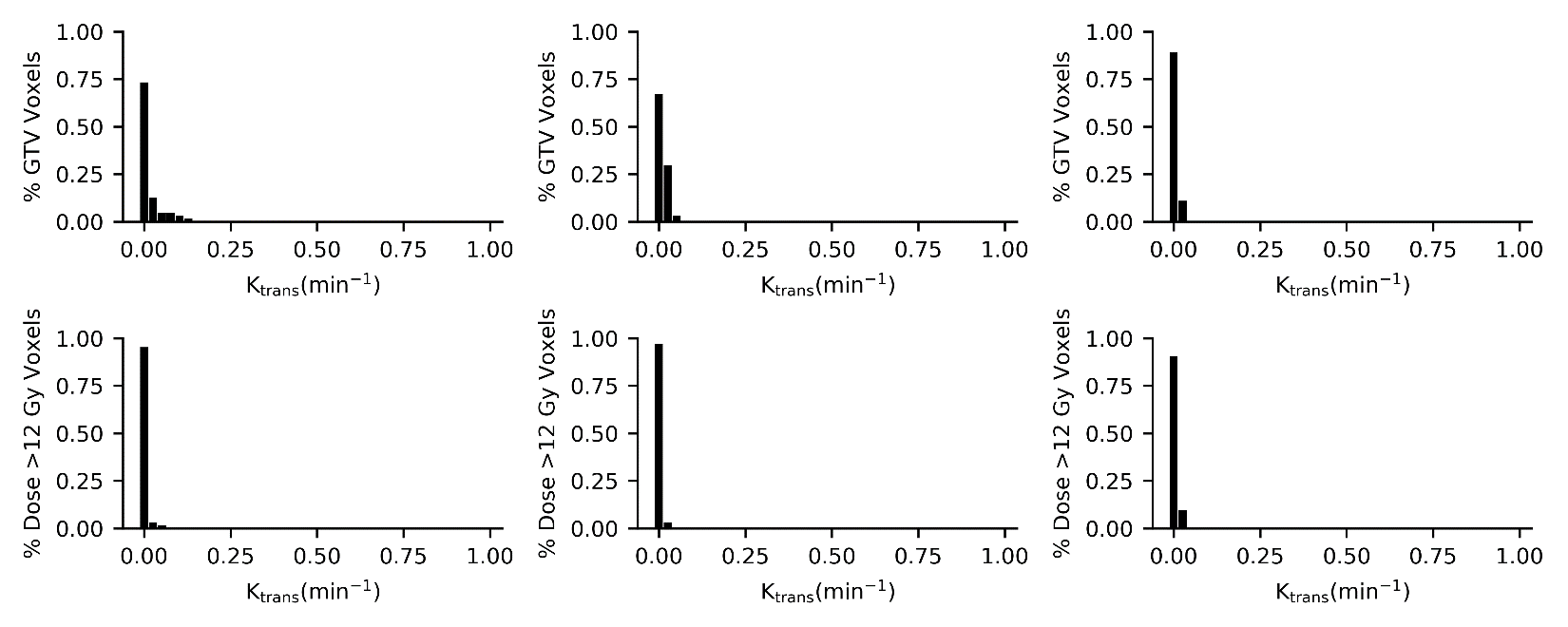
**

**
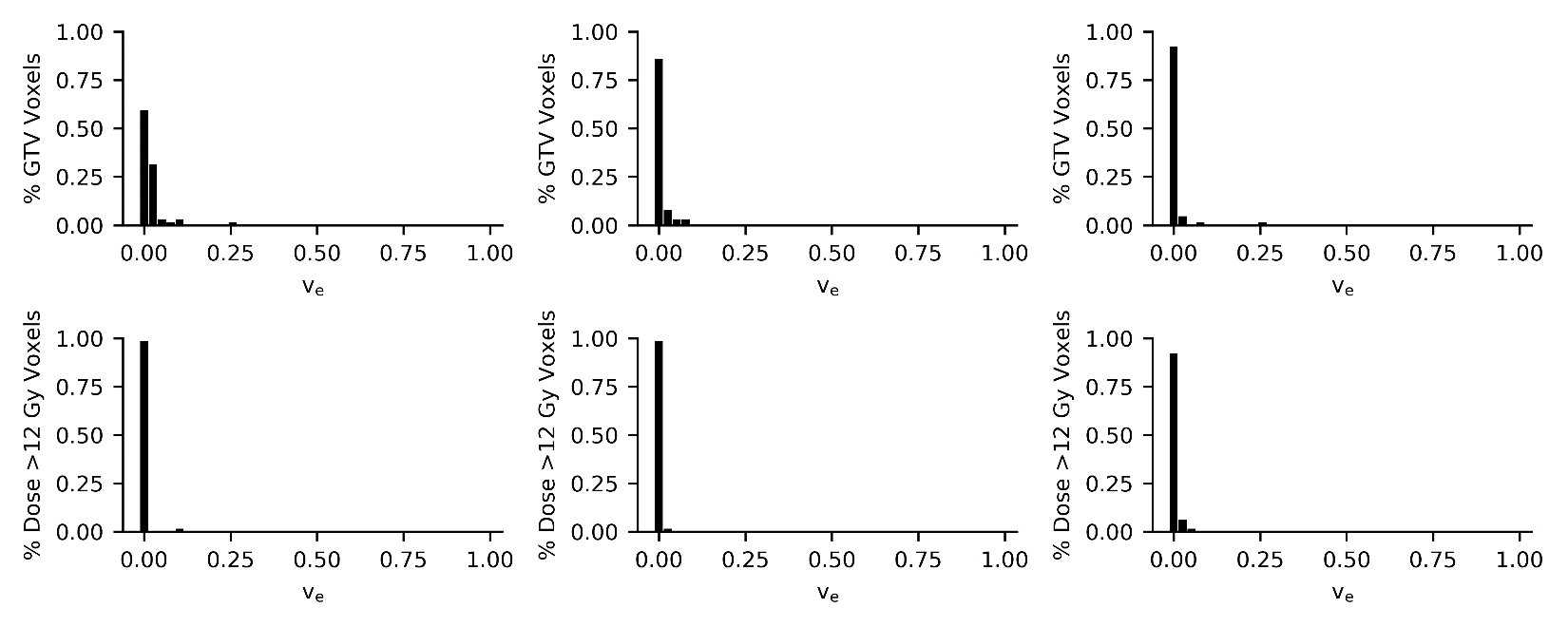
**

**Day 0 Day 3 Day 20**

**Patient 2: Metastasis 2**

**
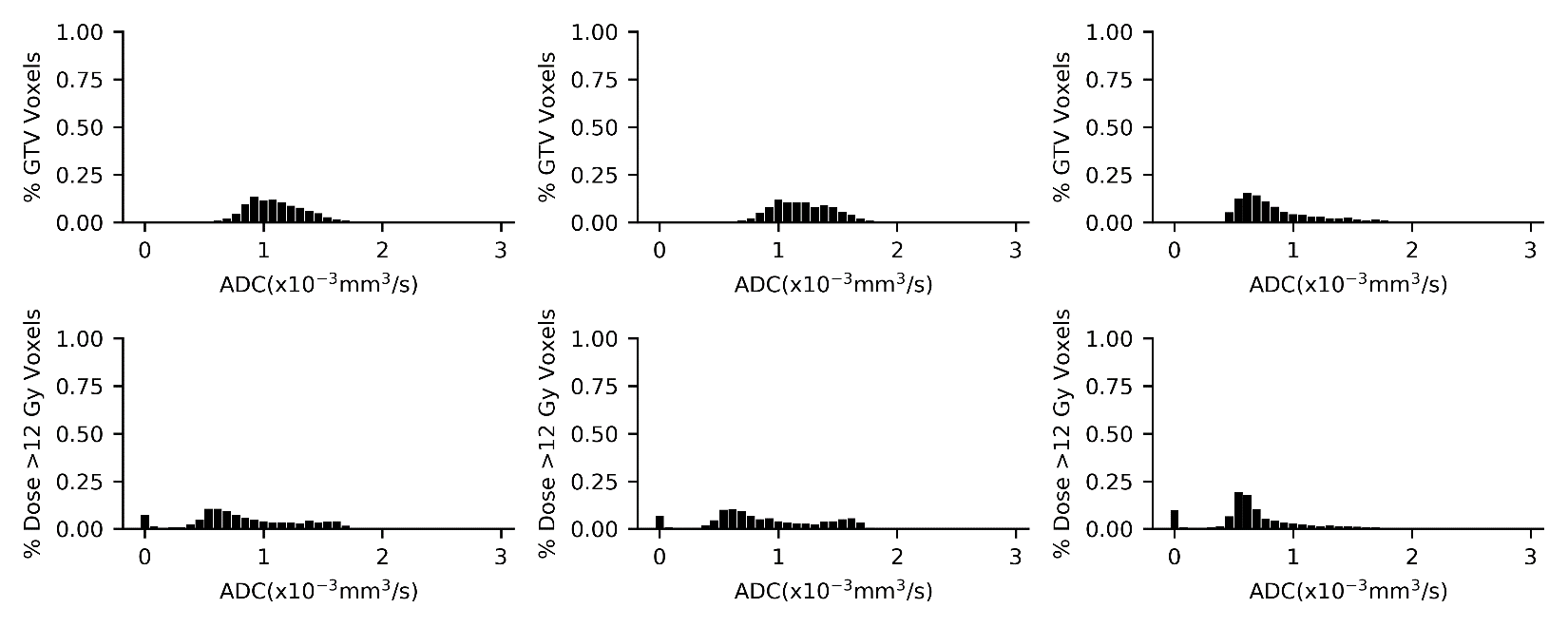

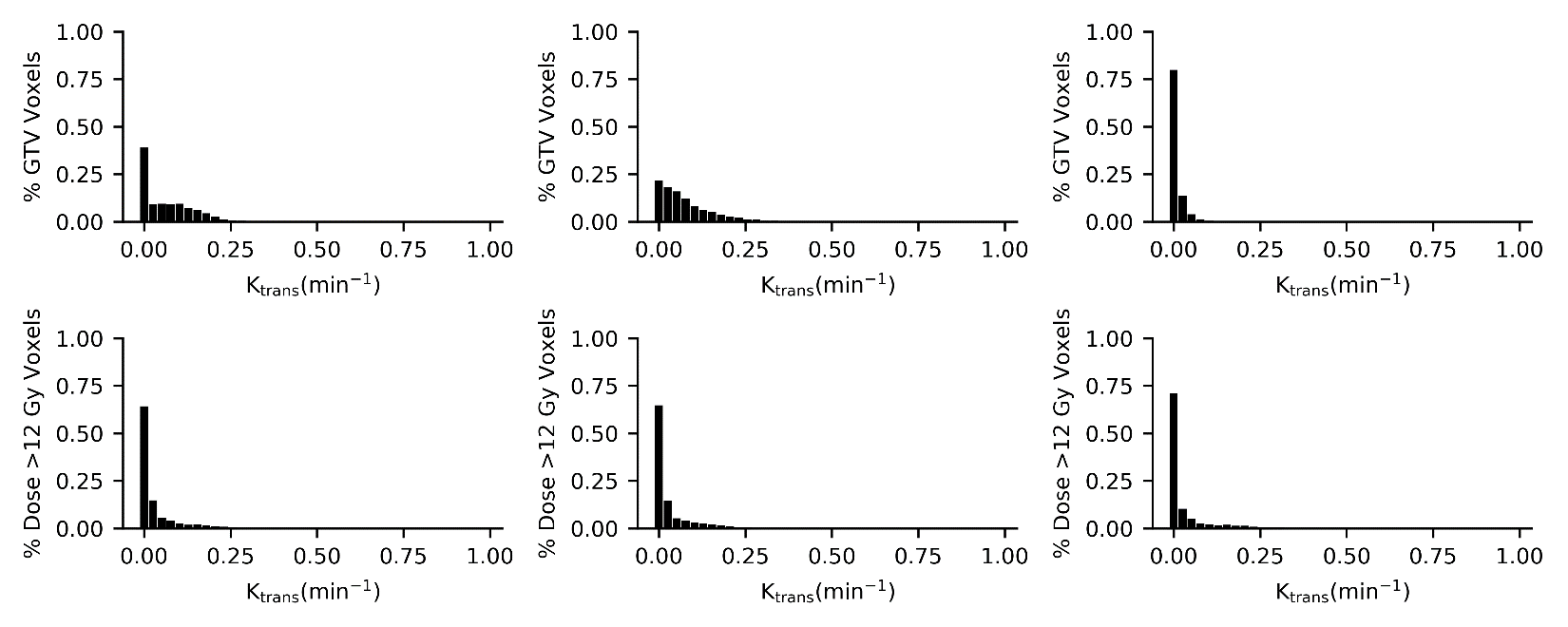
**

**
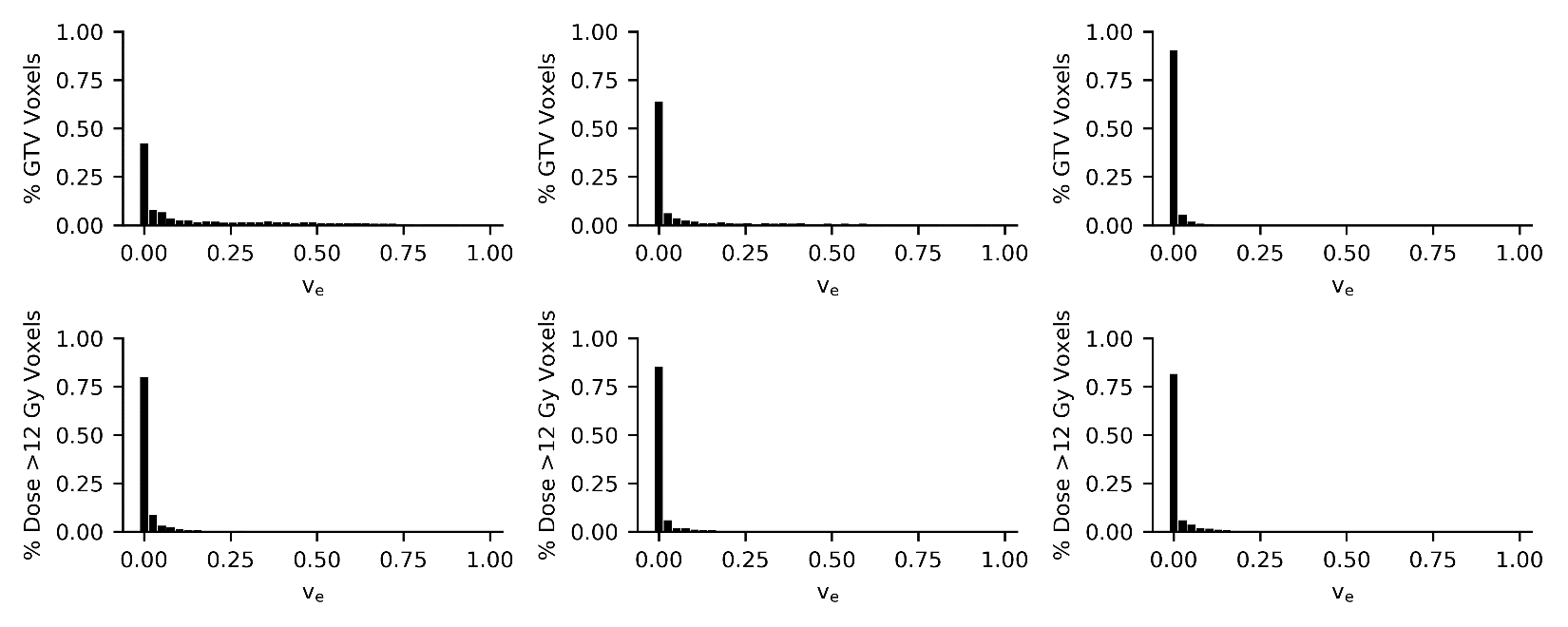
**

**Day 0 Day 3 Day 20**

**Patient 3: Metastasis 1
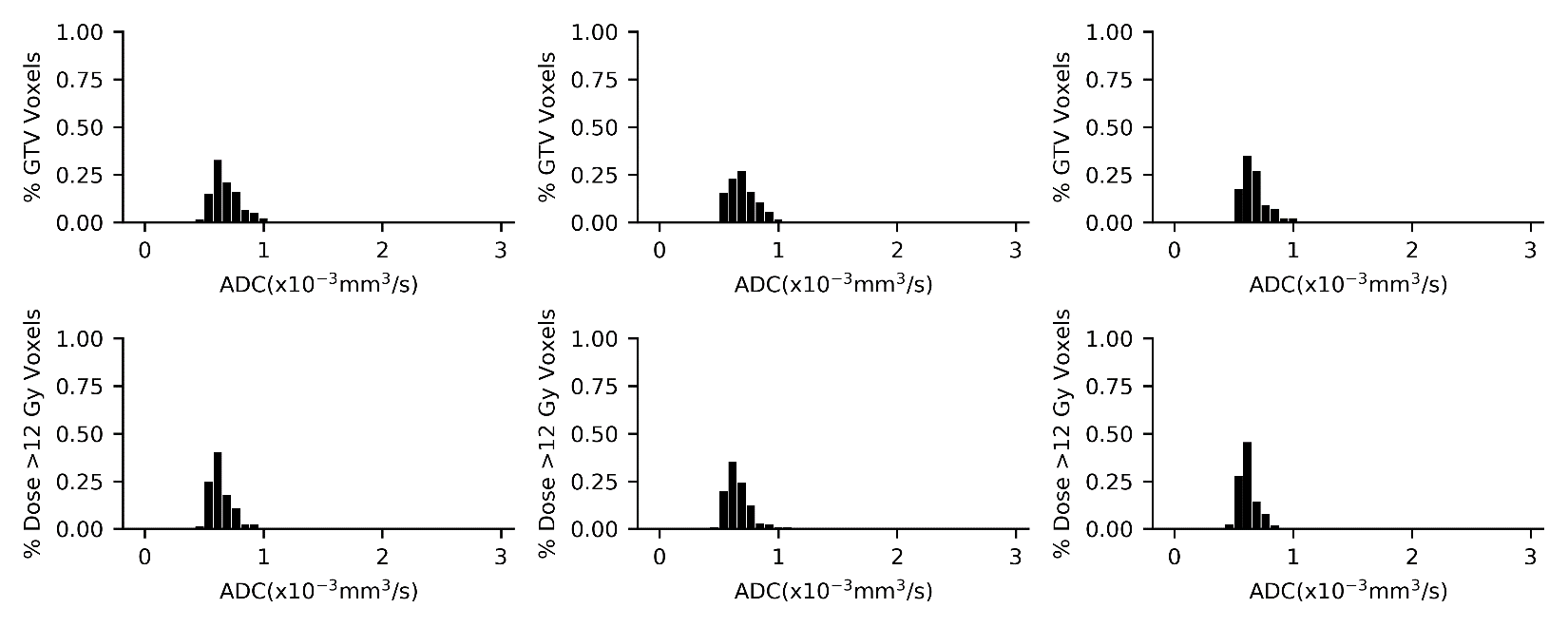

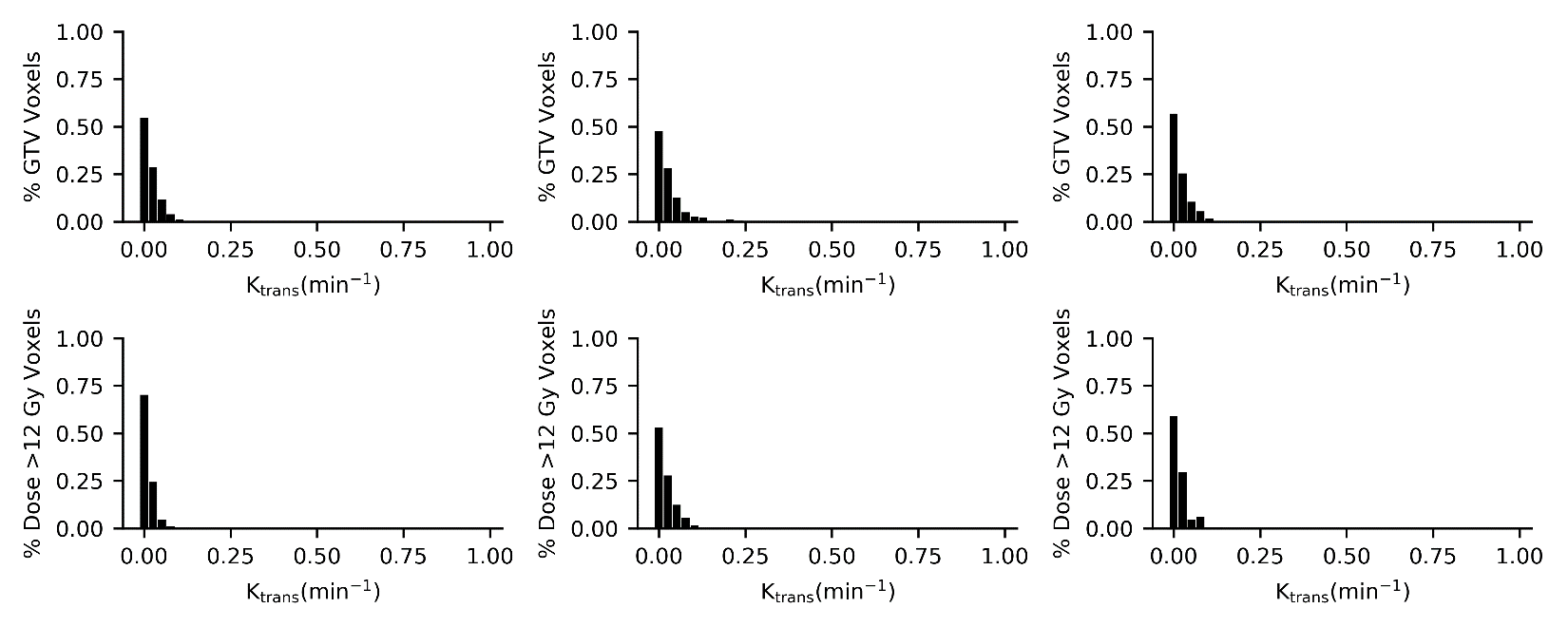
**

**
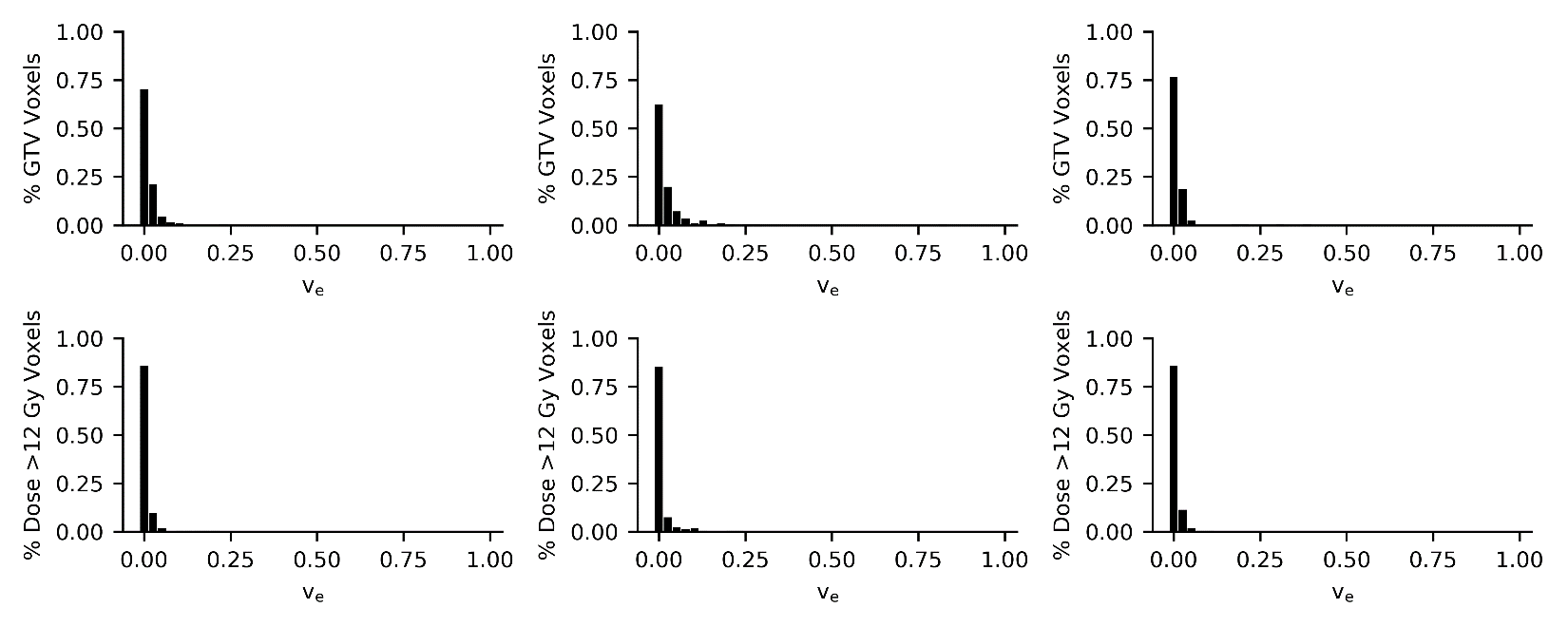
**

**Day 0 Day 3 Day 20**

**Patient 4: Metastasis 1
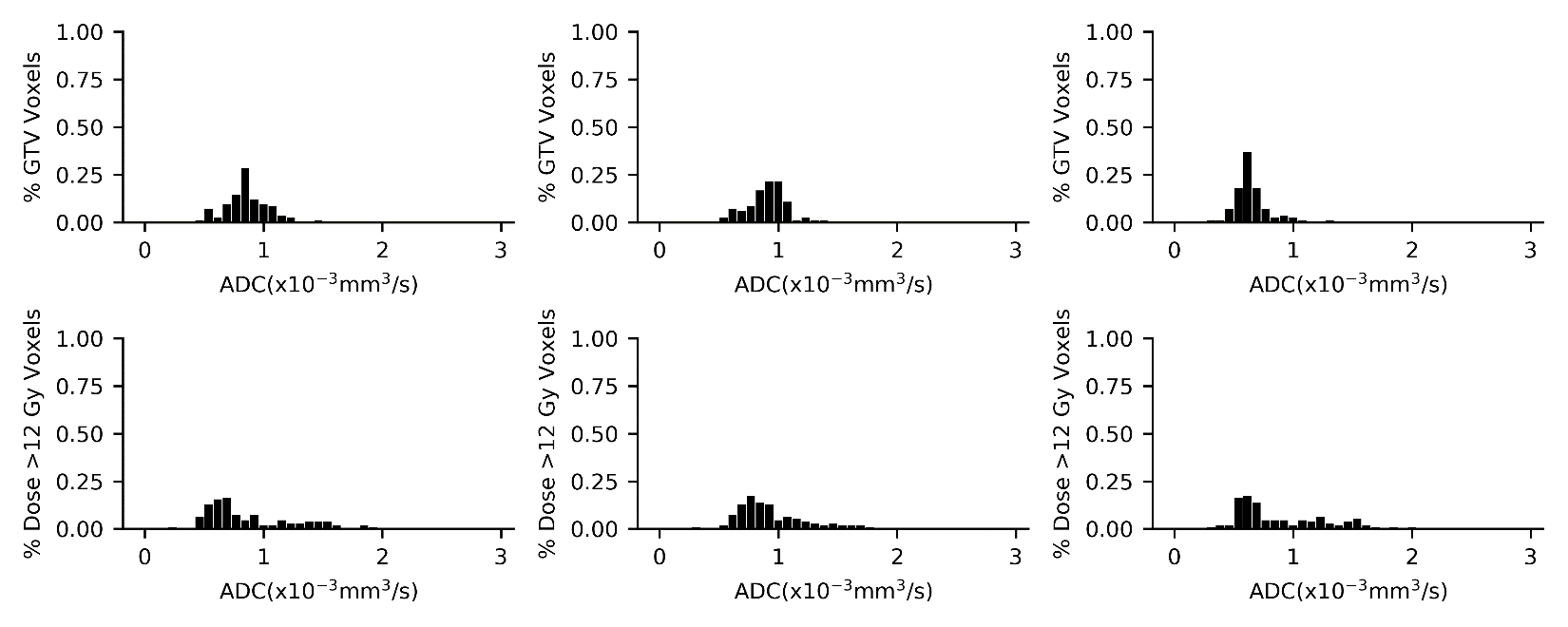

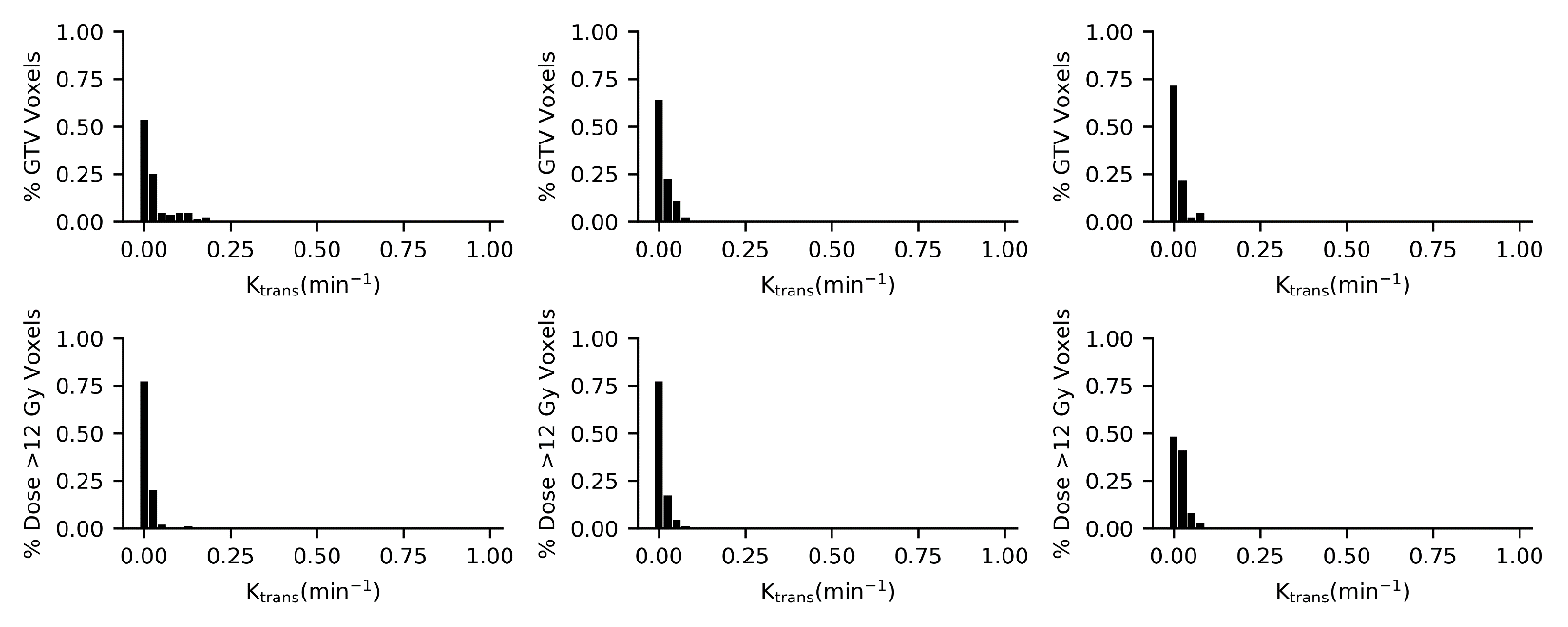

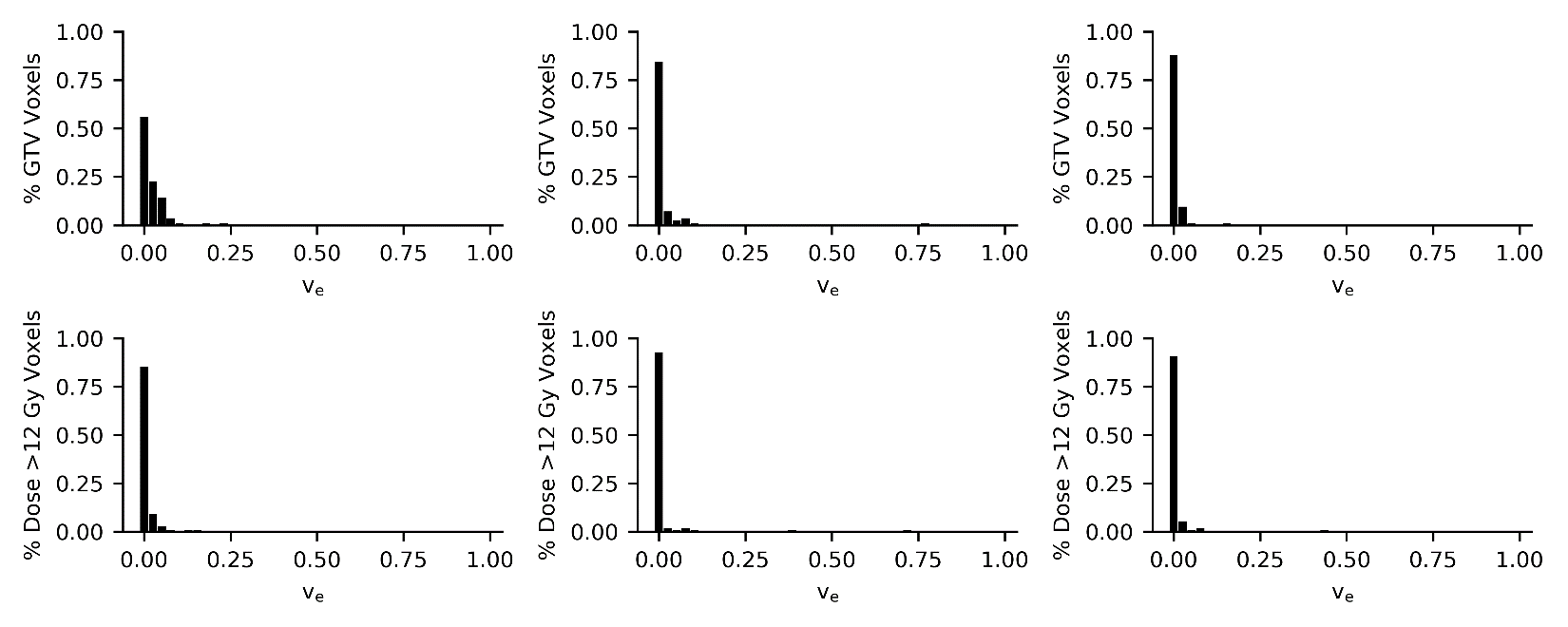
**

**Day 0 Day 3 Day 20**

**Patient 4: Metastasis 2
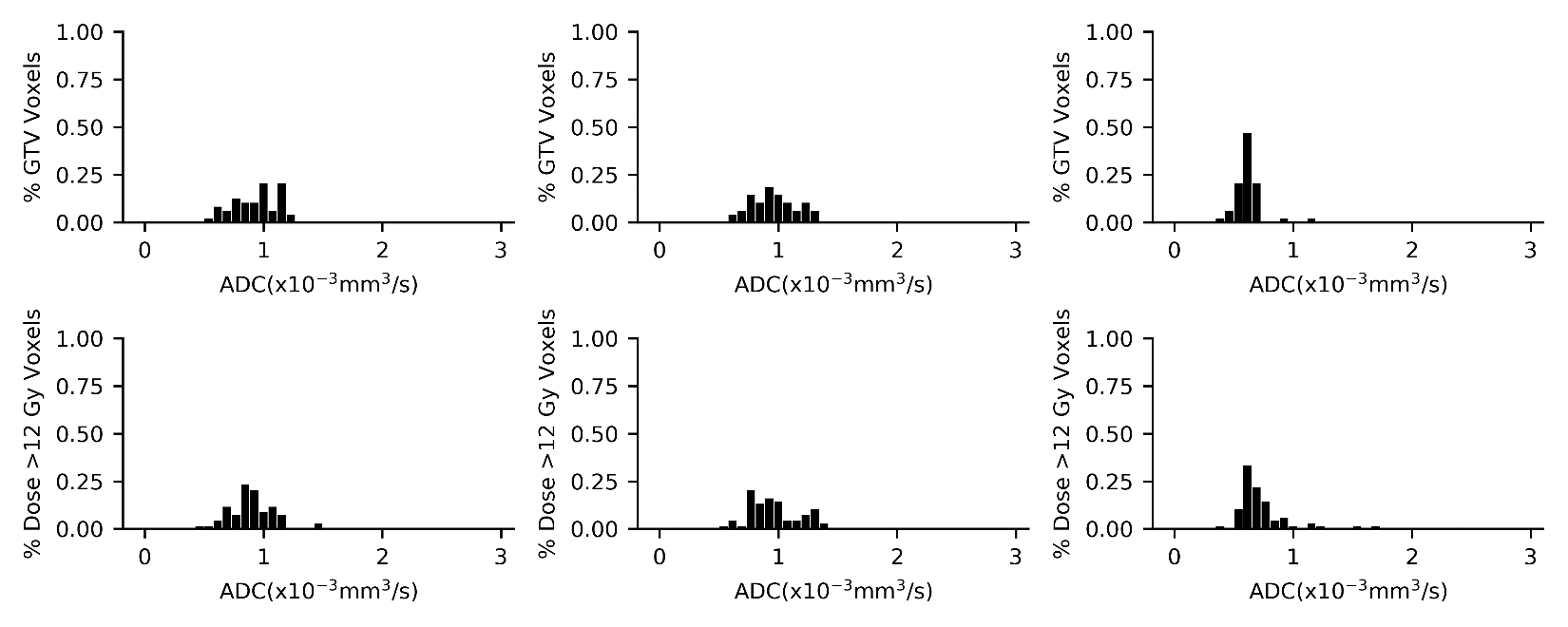

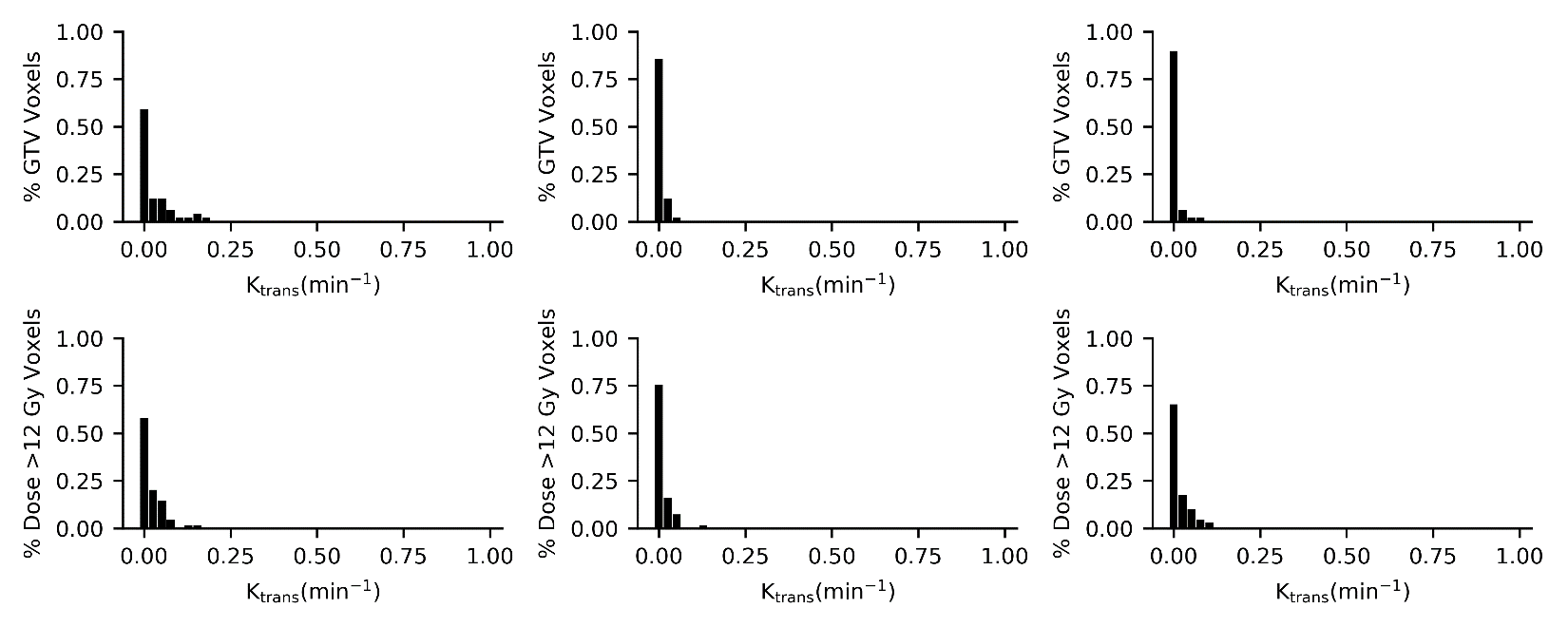
**

**
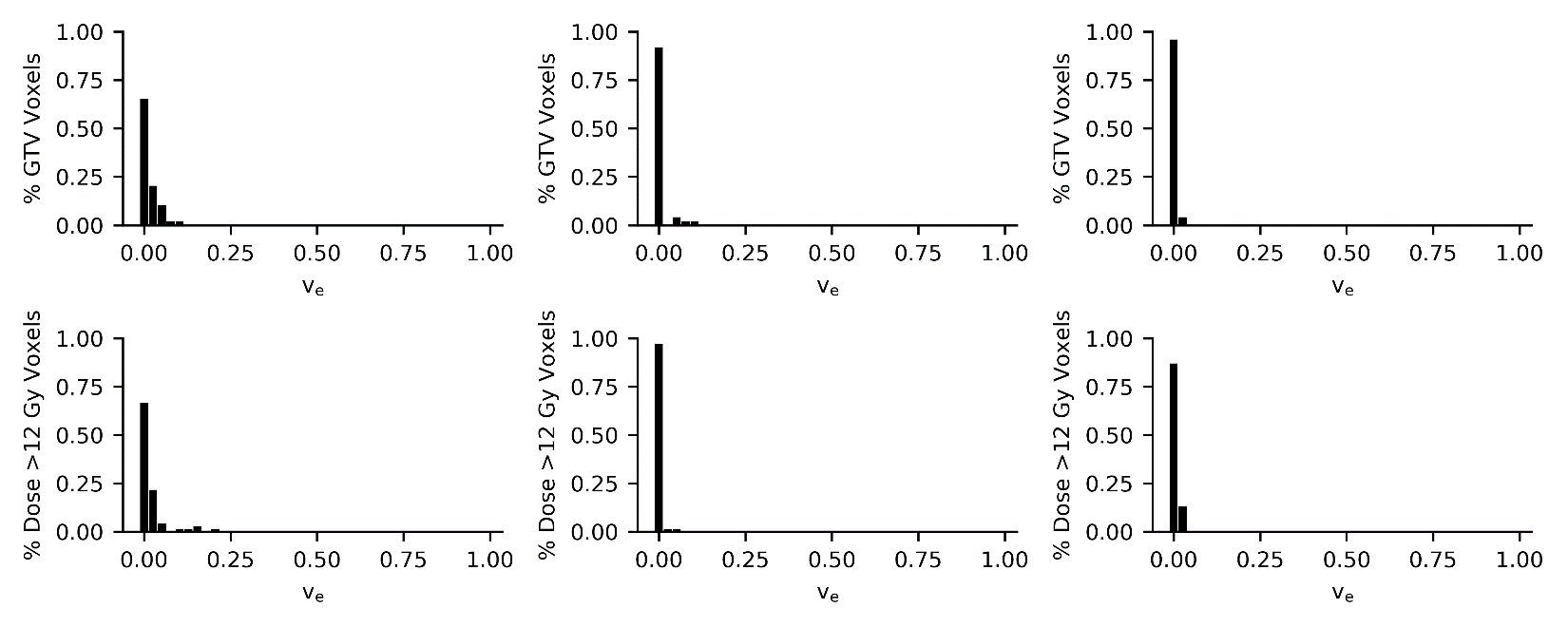
**

**Day 0 Day 3 Day 20**

**Patient 5: Metastasis 1
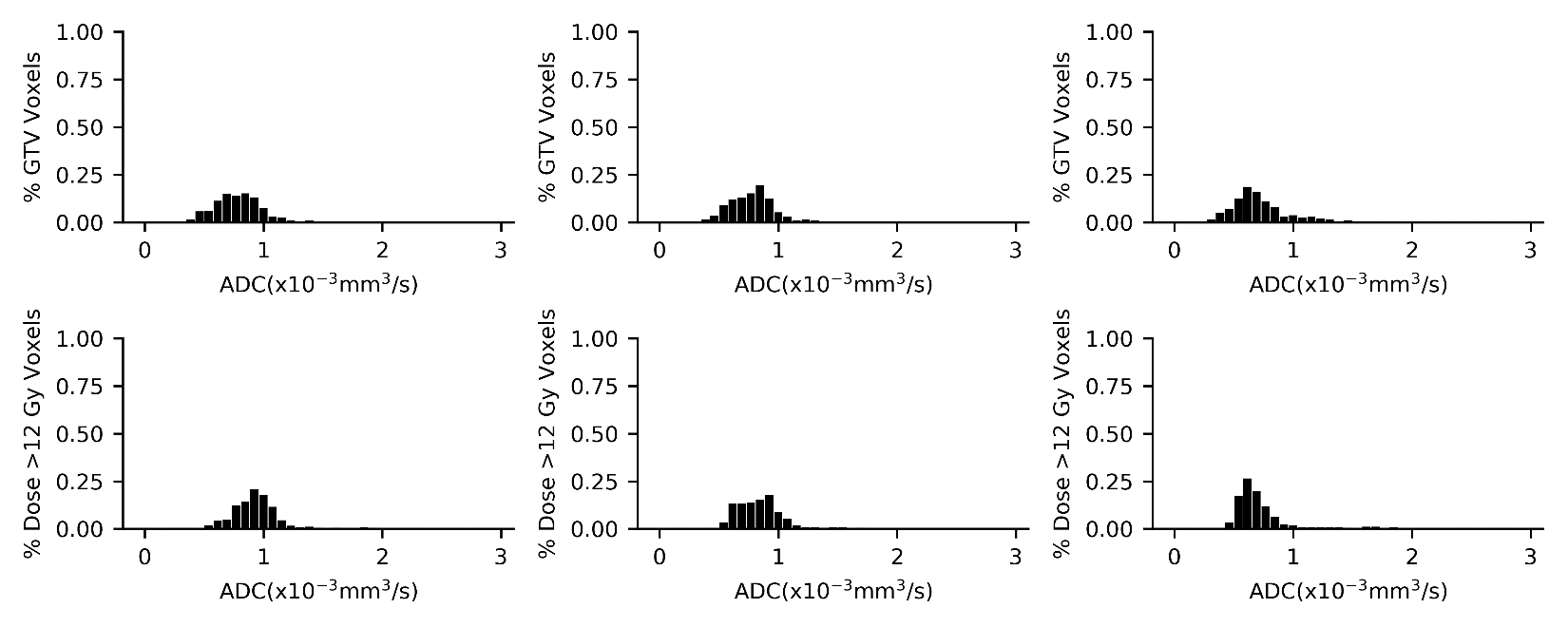
**


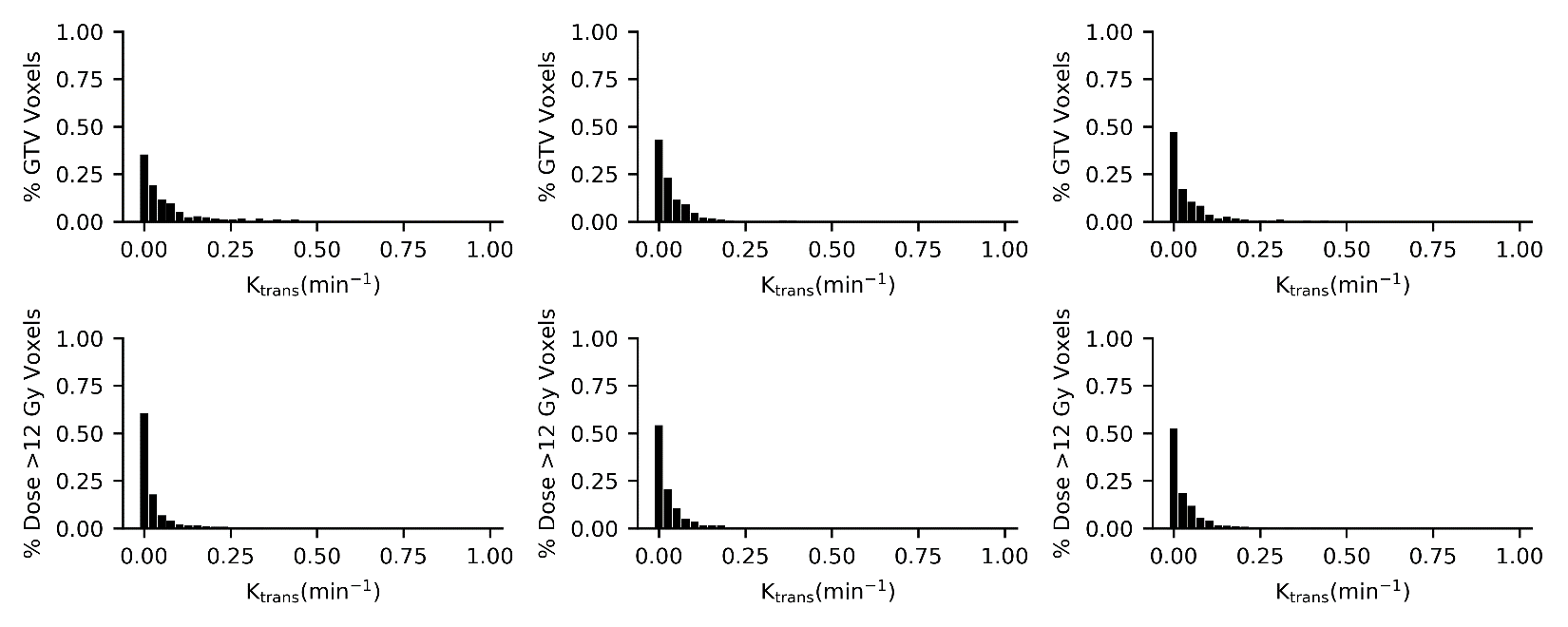


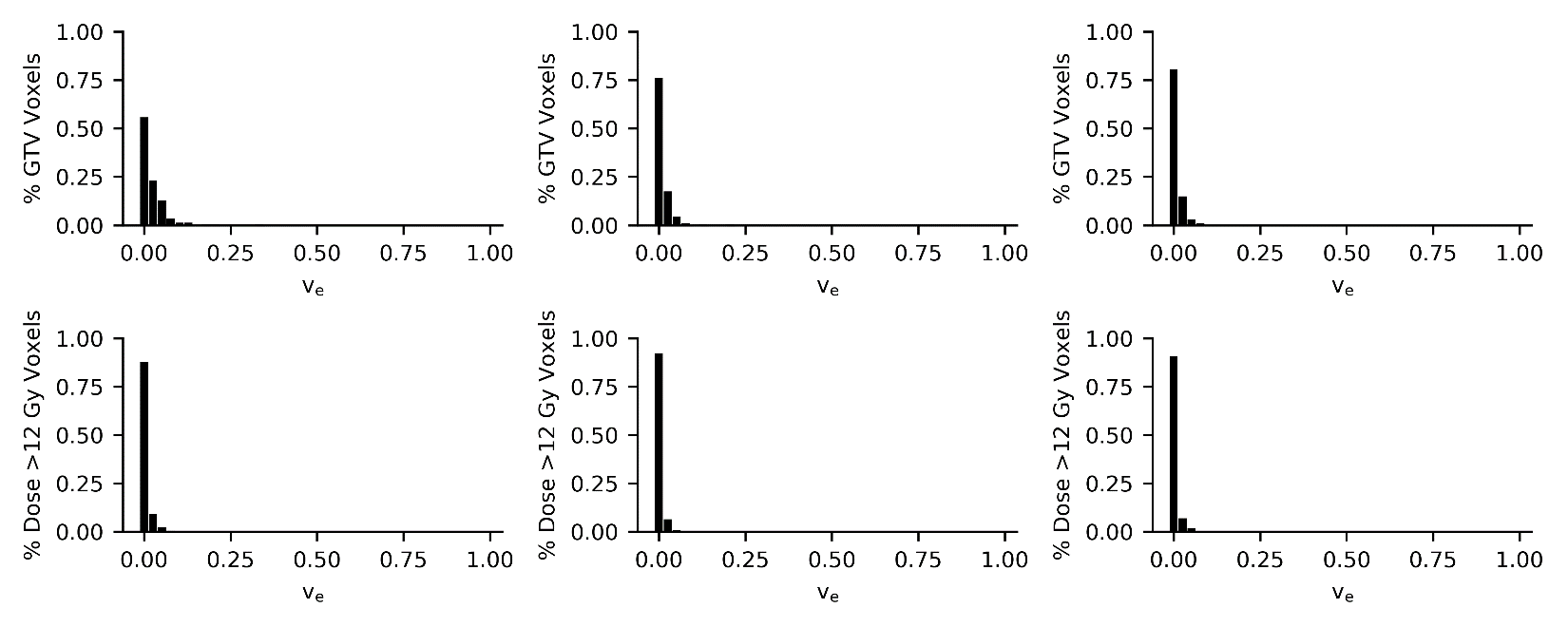


**Day 0 Day 3 Day 20**

**Patient 5: Metastasis 2
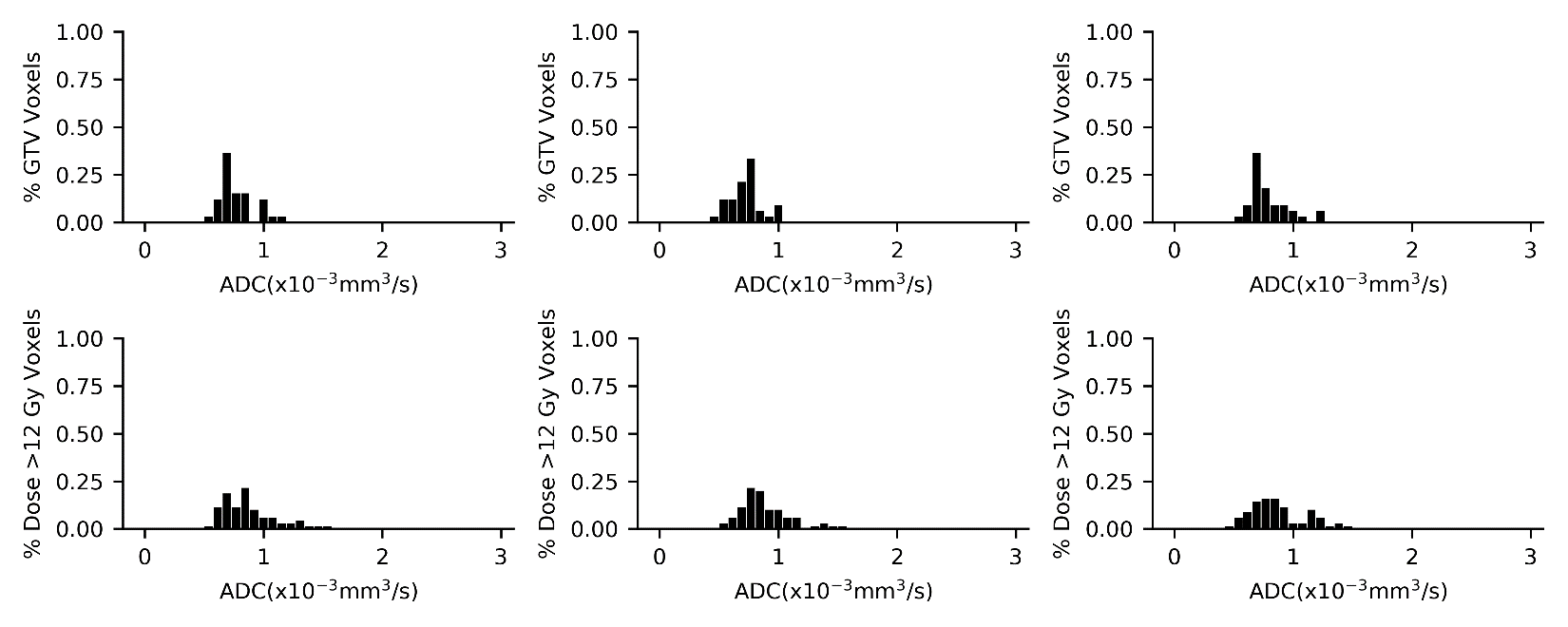
**

**
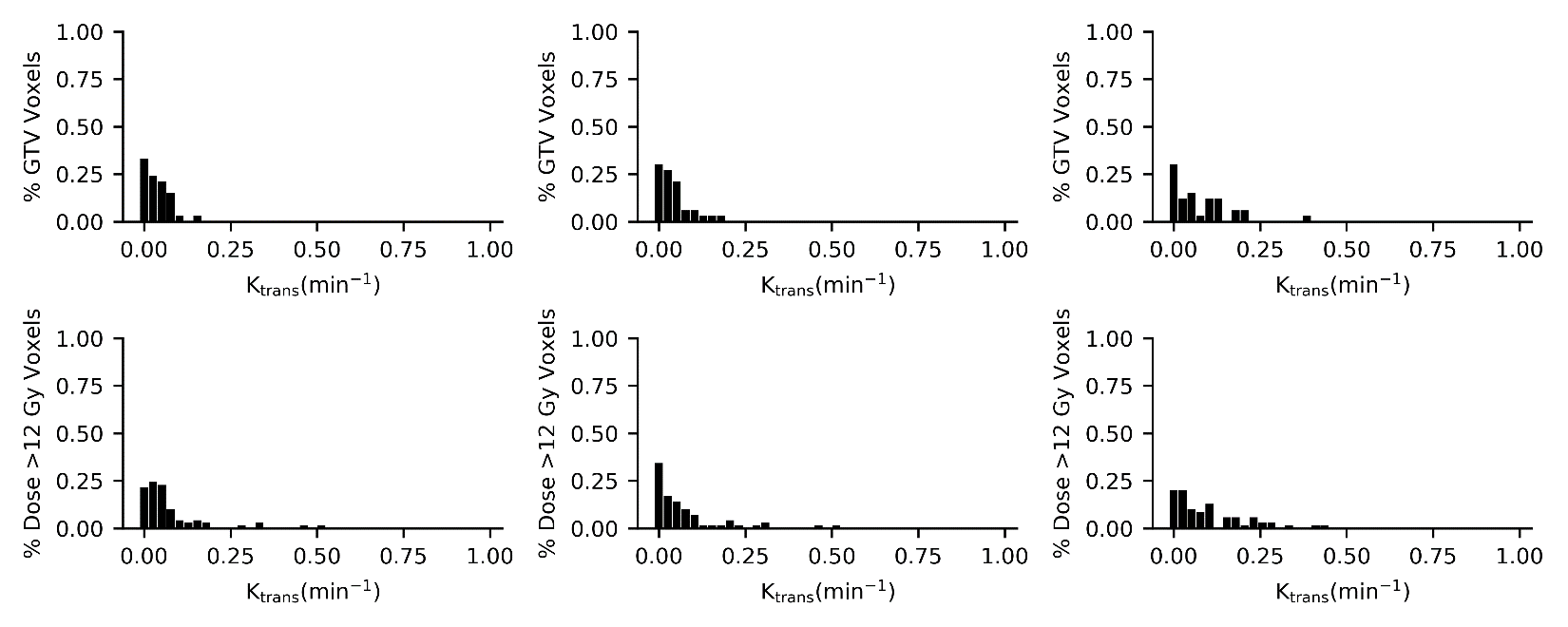
**

**
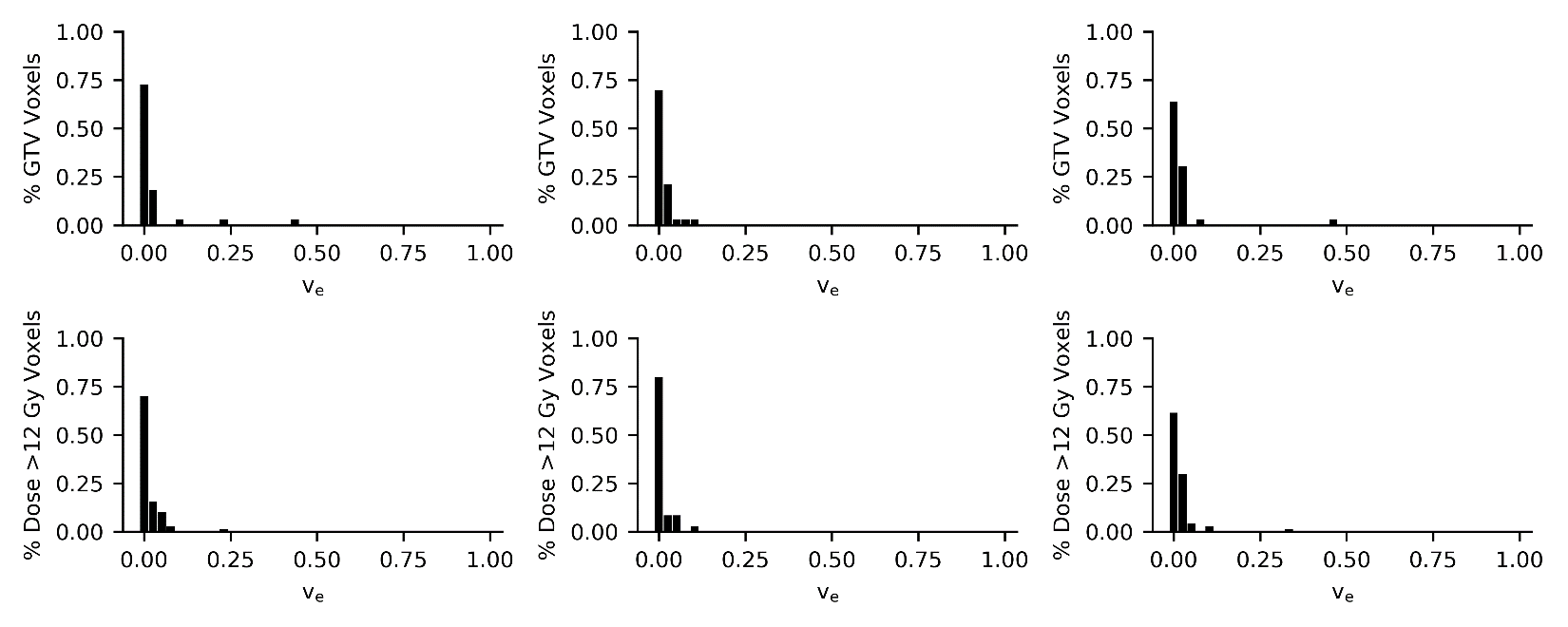
**

**Day 0 Day 3 Day 20**

**Patient 5: Metastasis 3
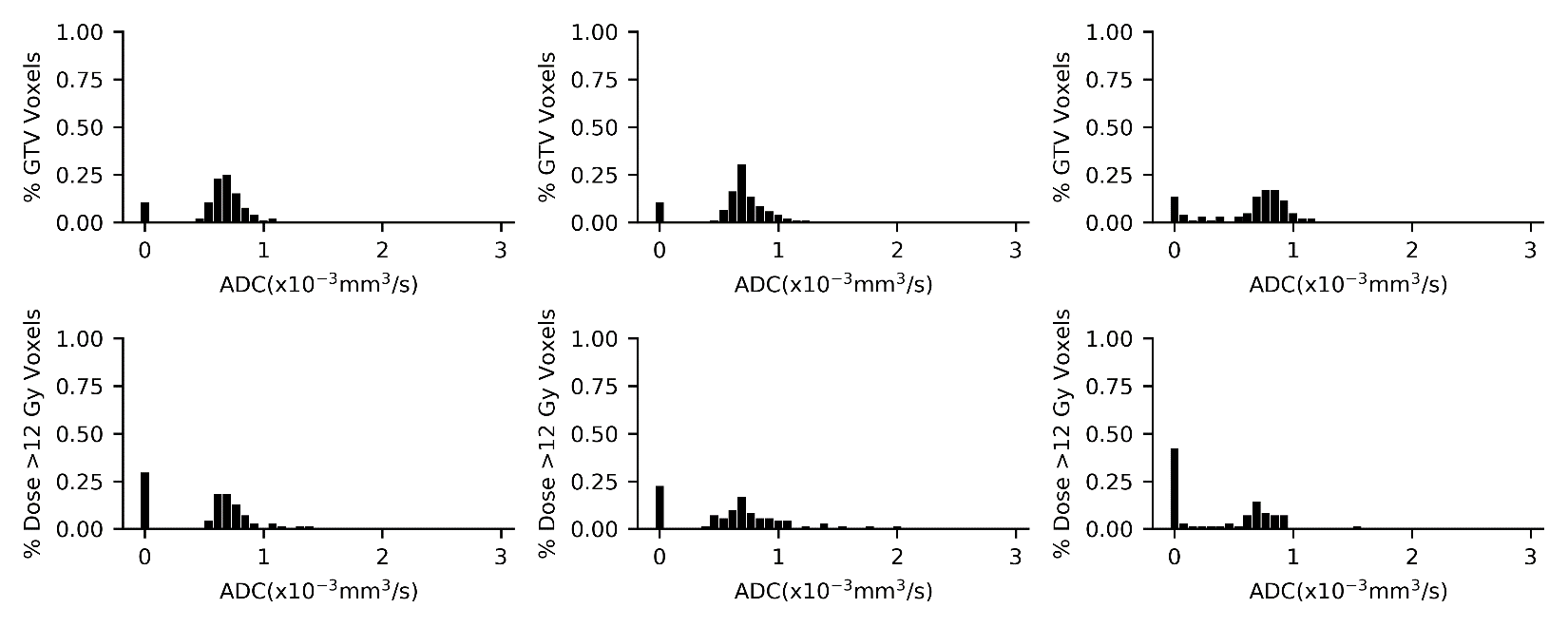

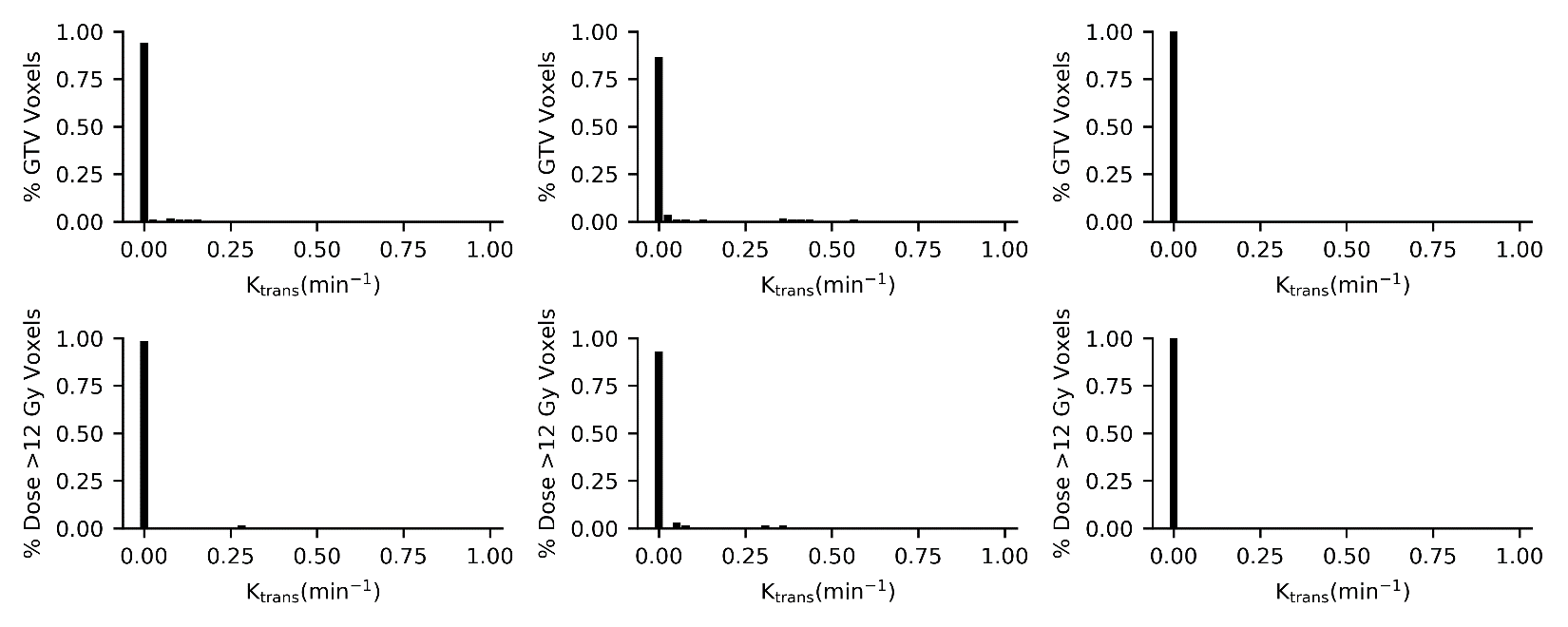

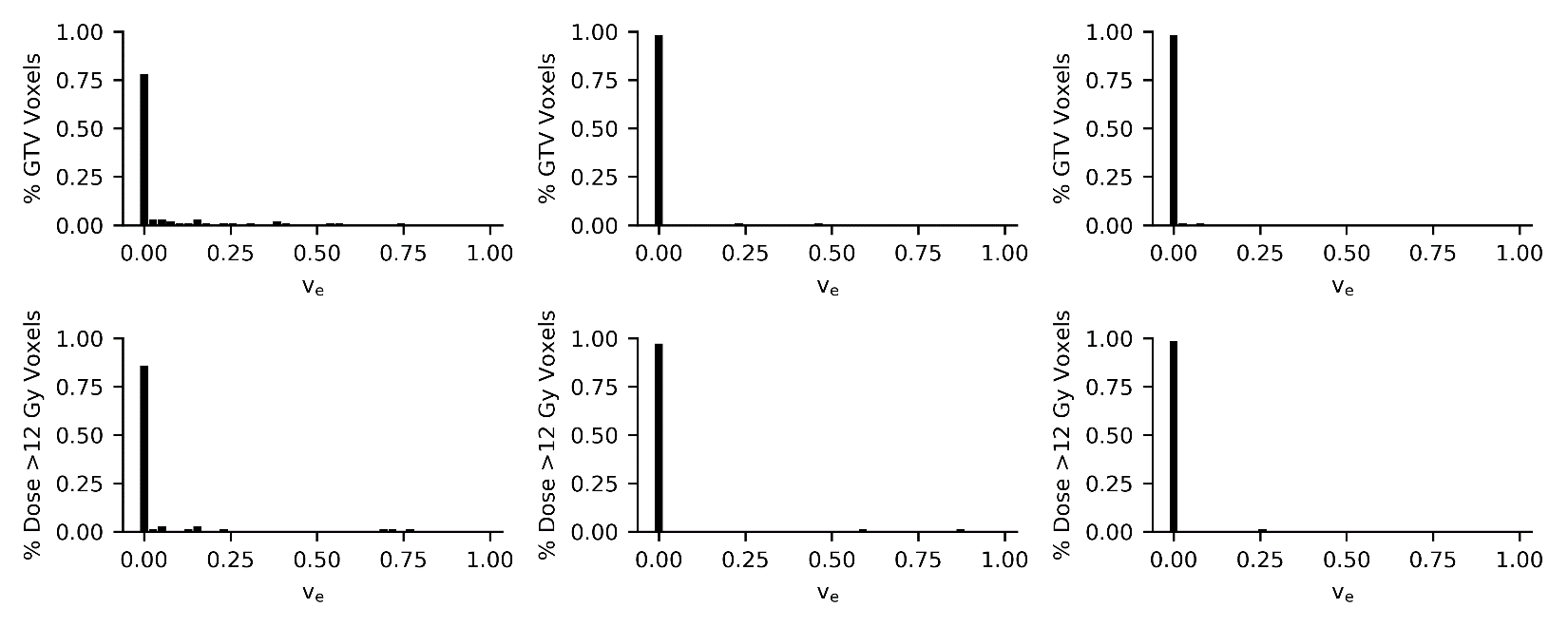
**

**Day 0 Day 3 Day 20**

**Patient 6: Metastasis 1
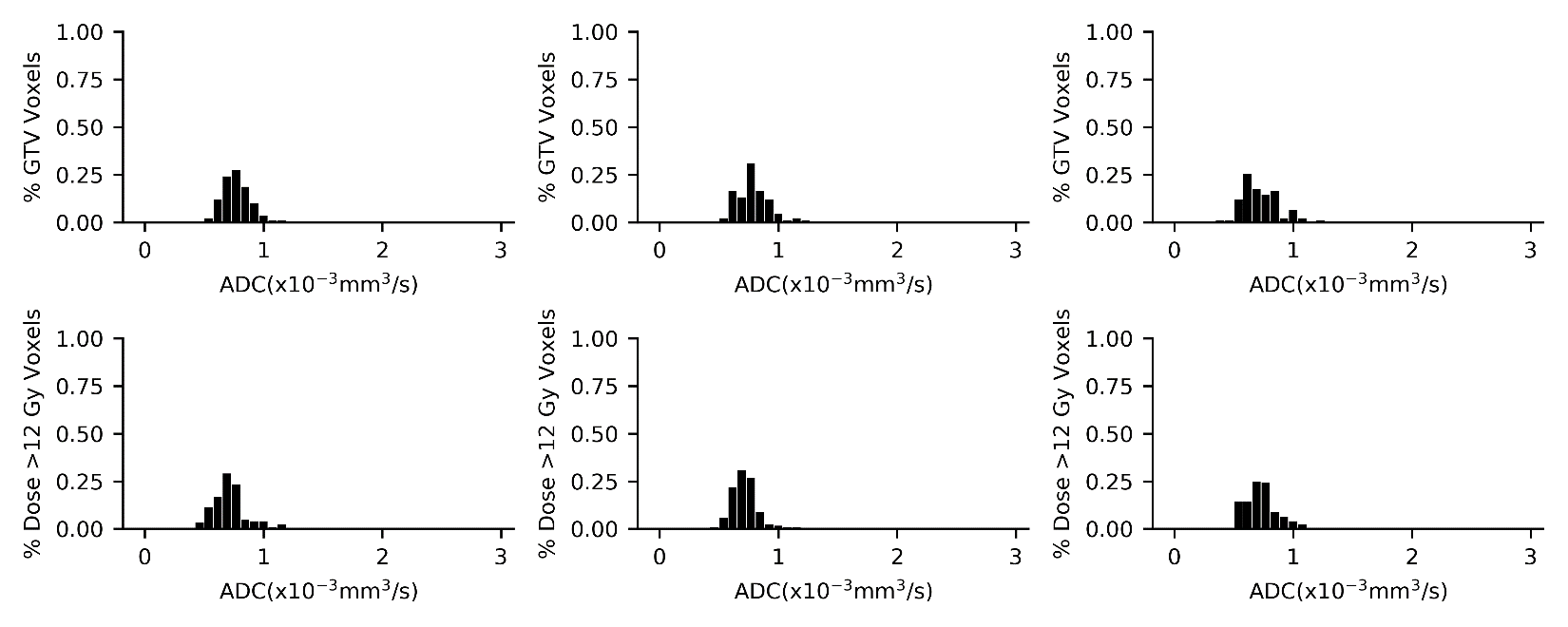
**
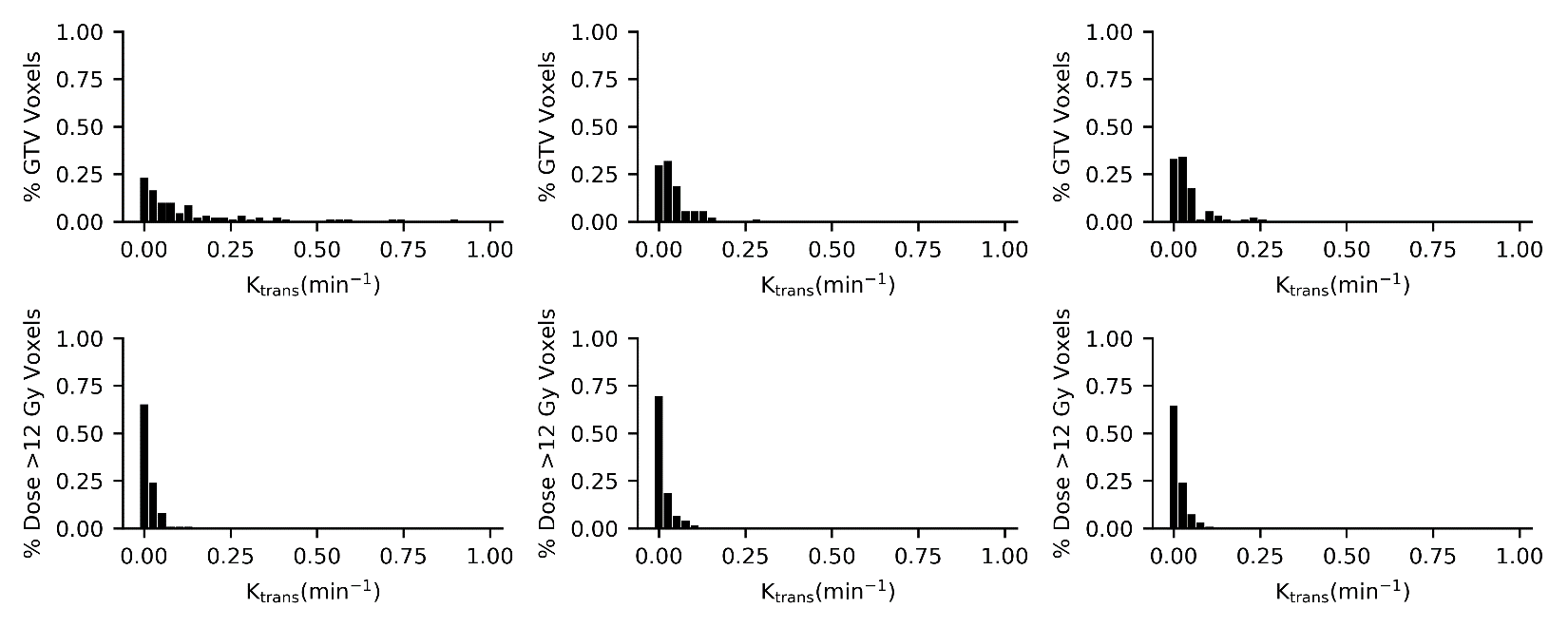


**
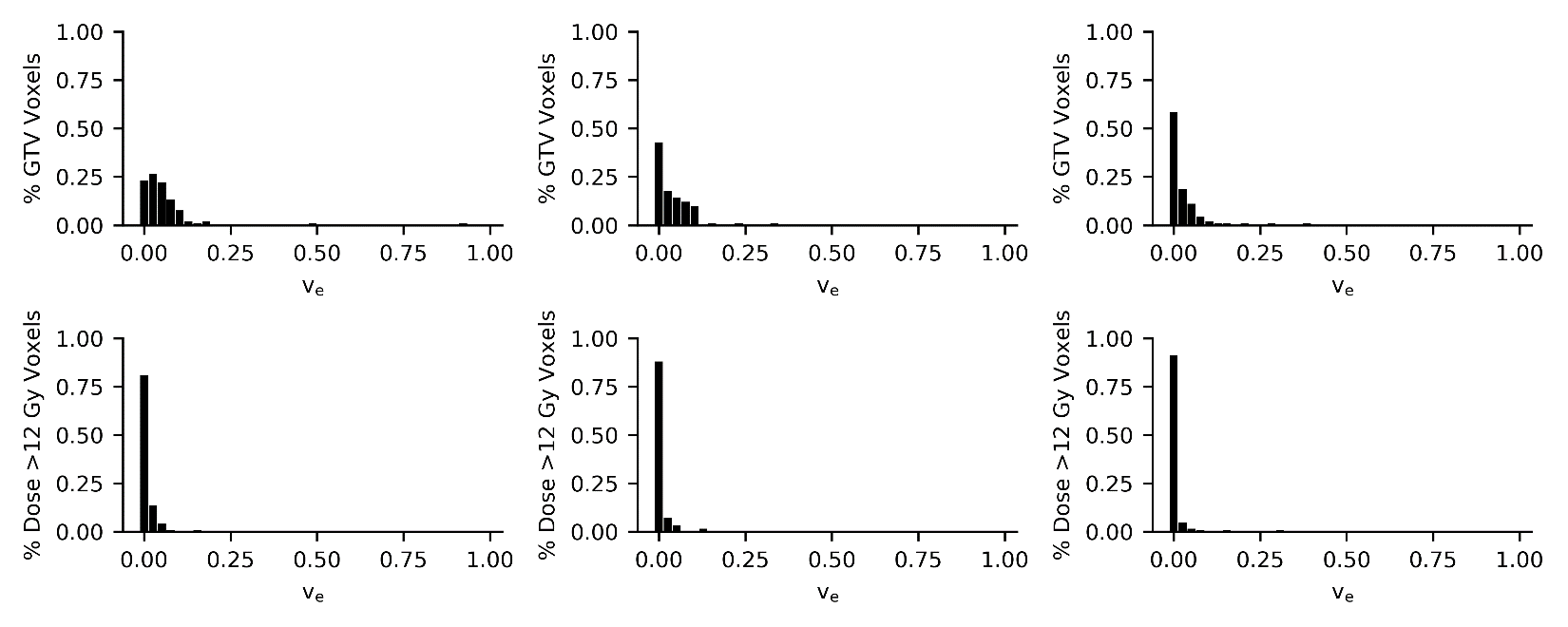
**

**Day 0 Day 3 Day 20**

**Patient 7: Metastasis 1
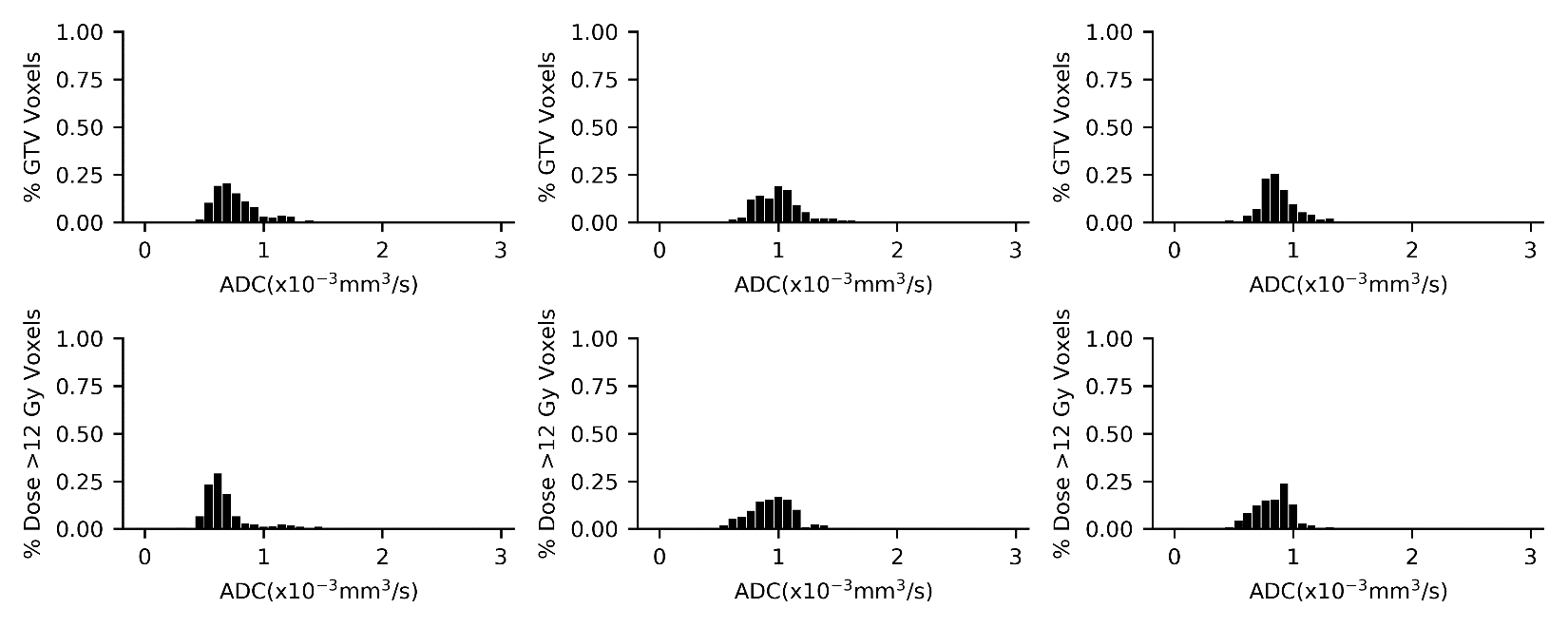
**
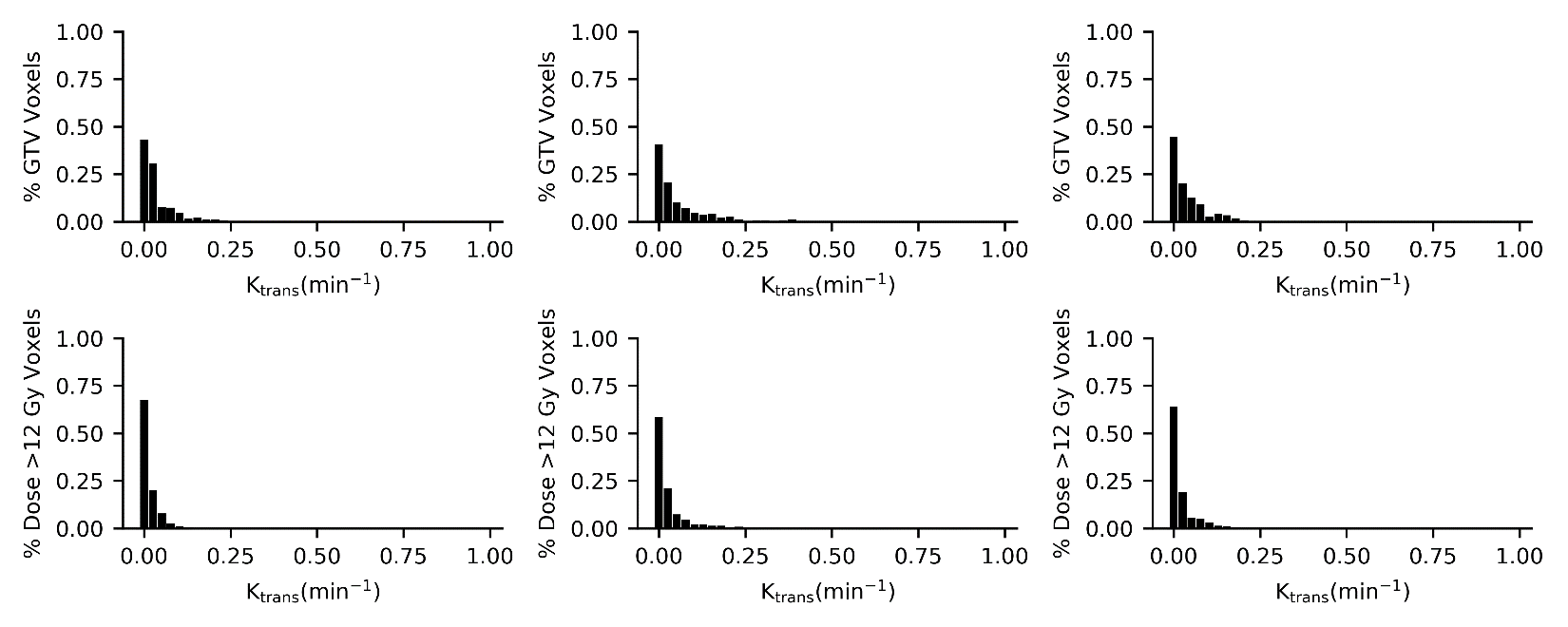


**
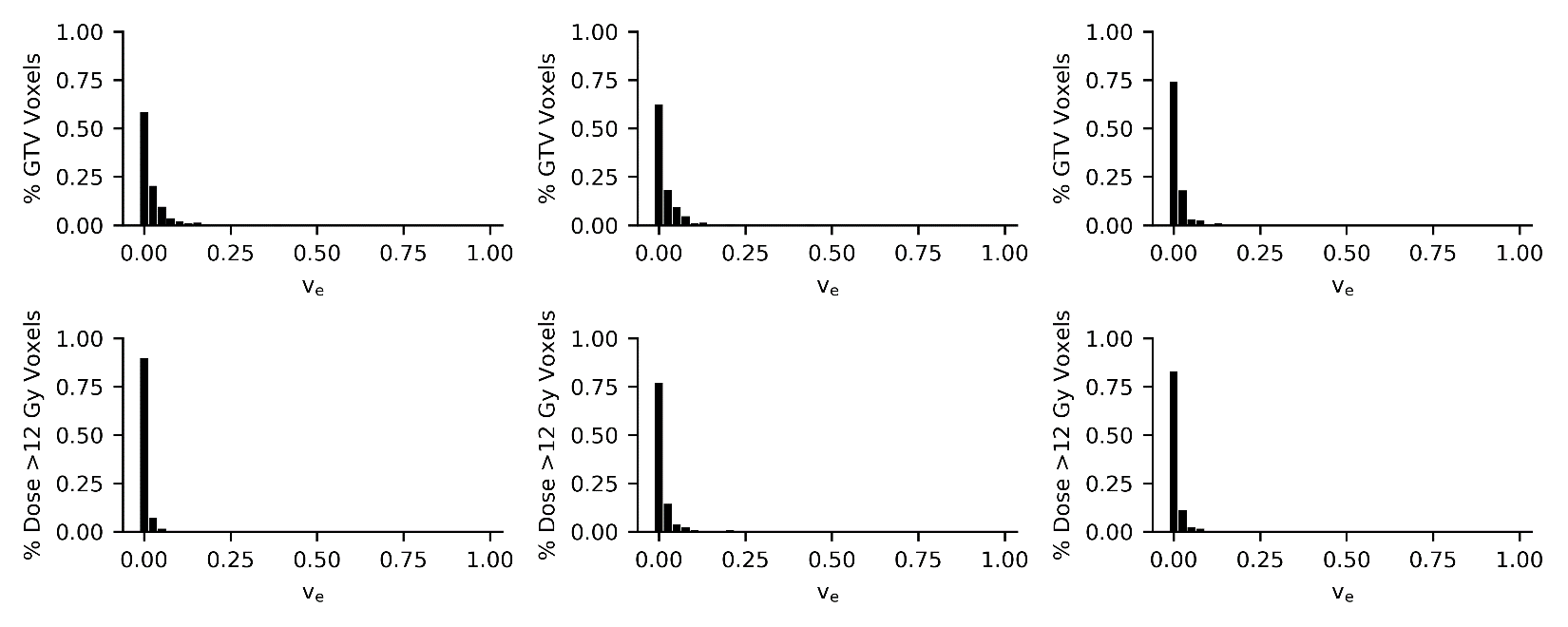
**

**Day 0 Day 3 Day 20**

**Patient 8: Metastasis 1
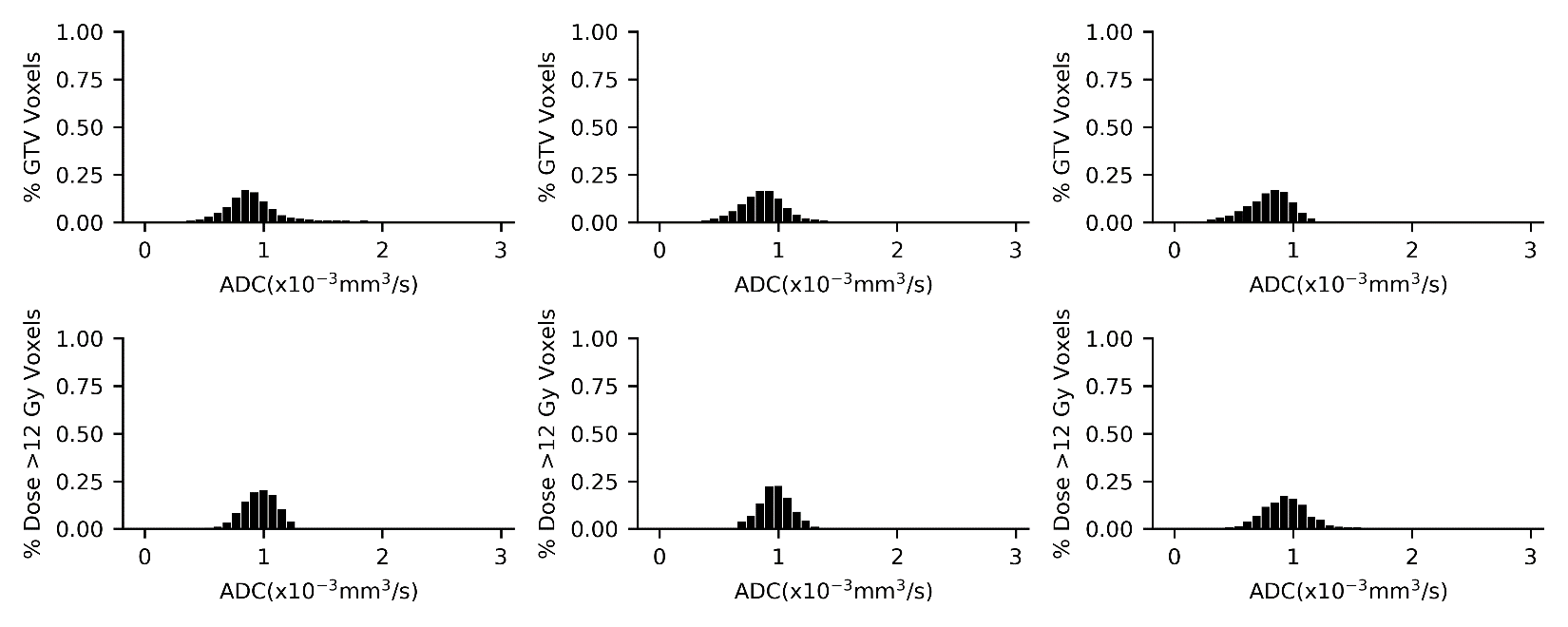
**

**
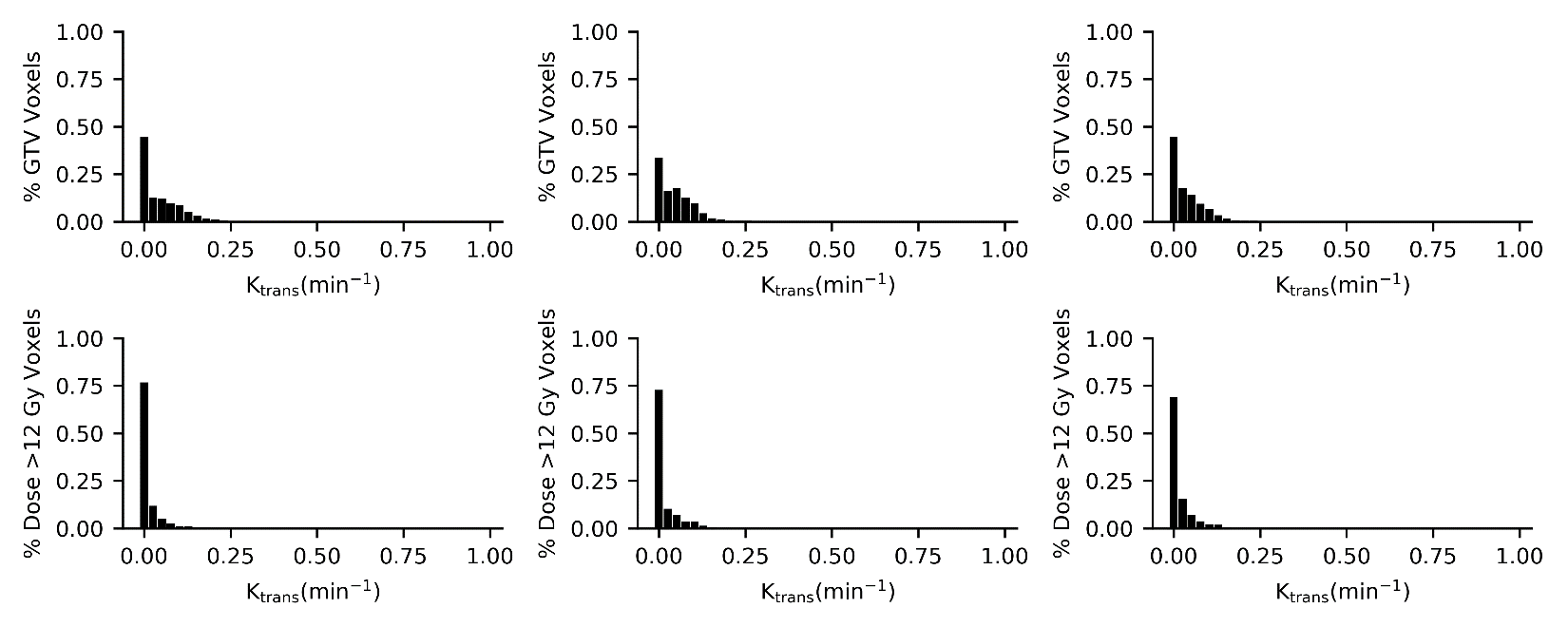
**

**
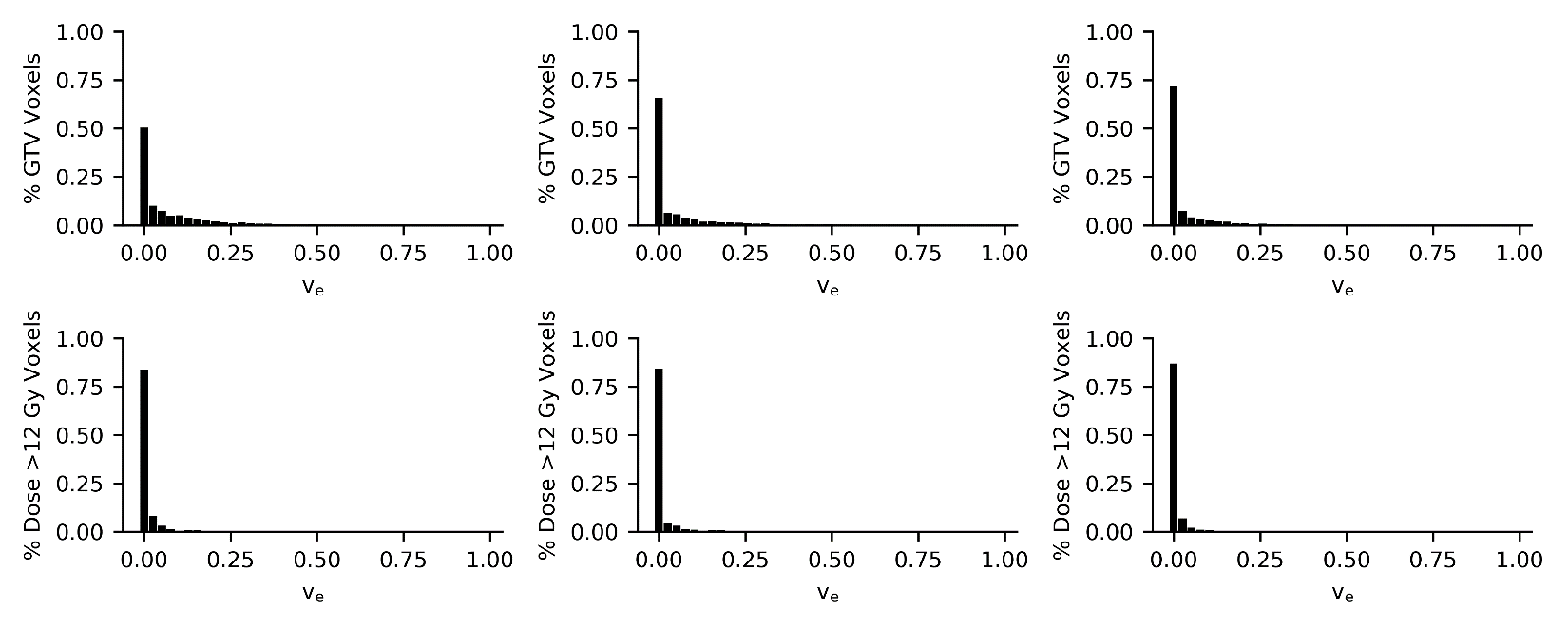
**

**Day 0 Day 3 Day 20**

**Patient 9: Metastasis 1
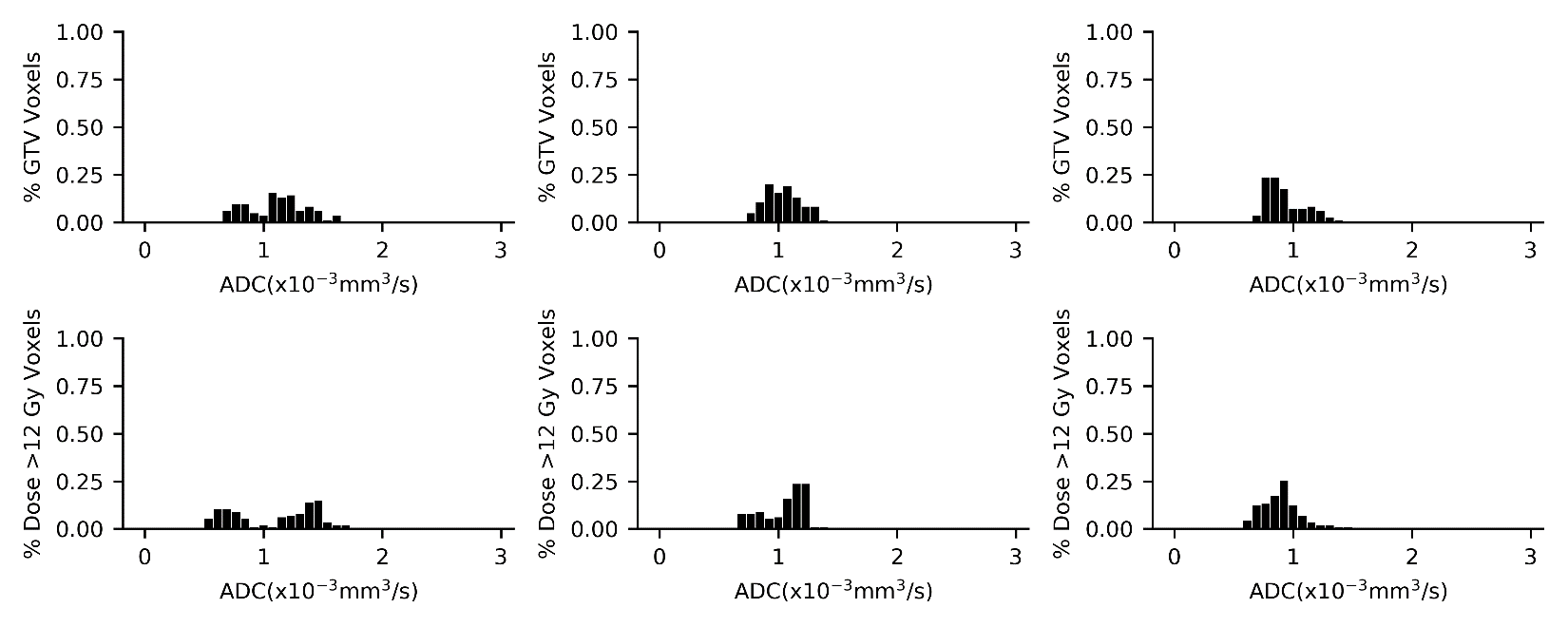
**


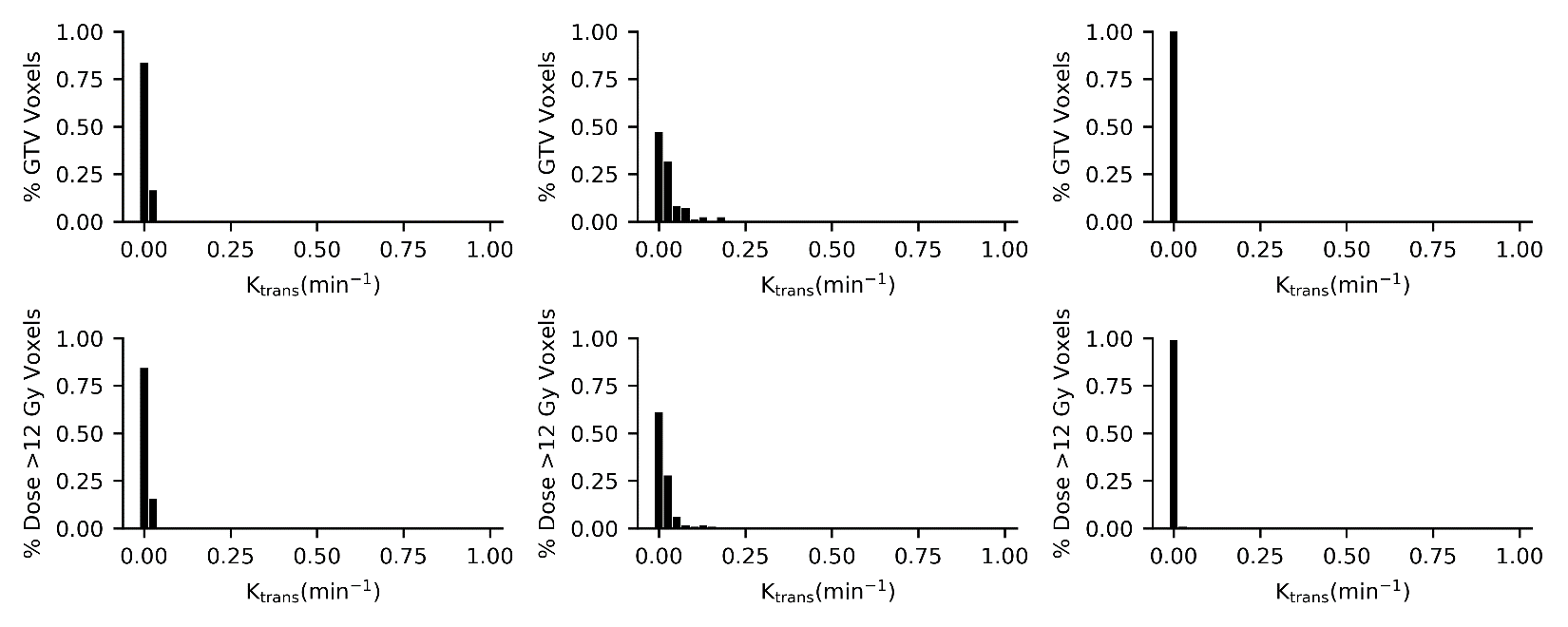


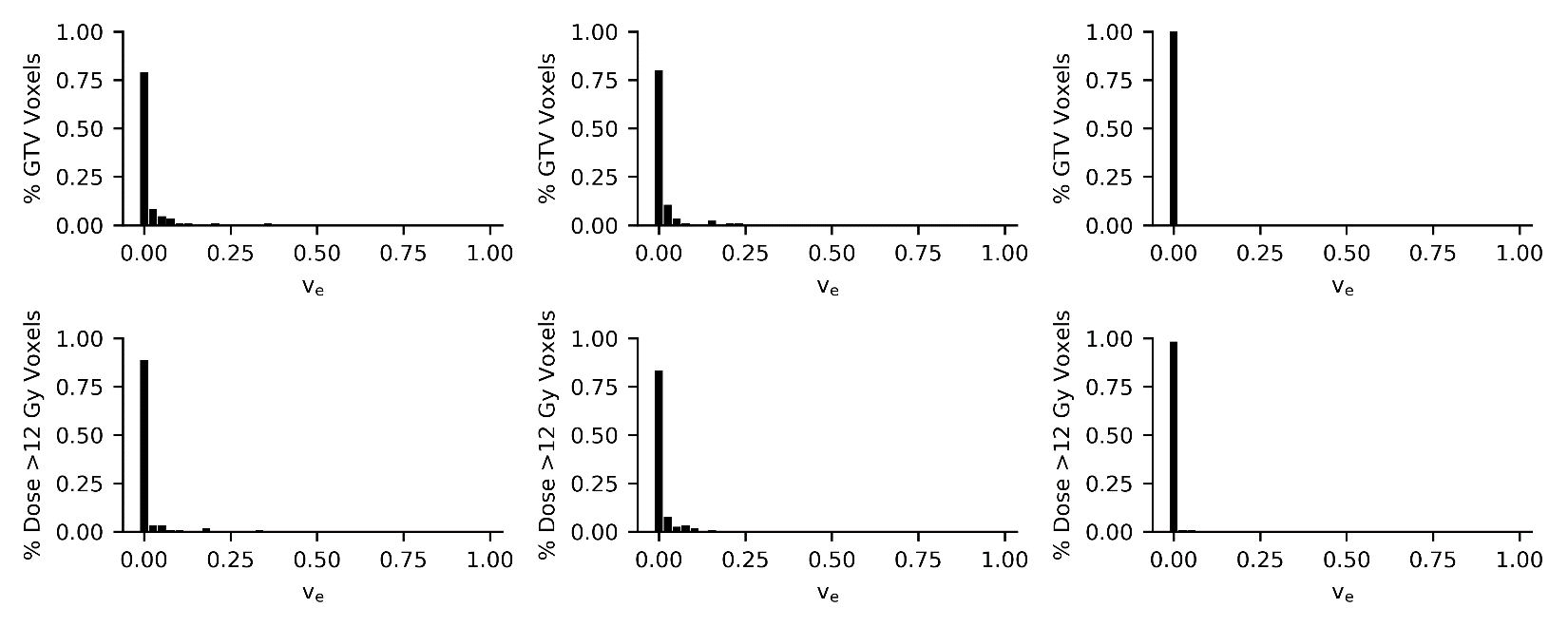


**Day 0 Day 3 Day 20**

**Patient 10: Metastasis 1
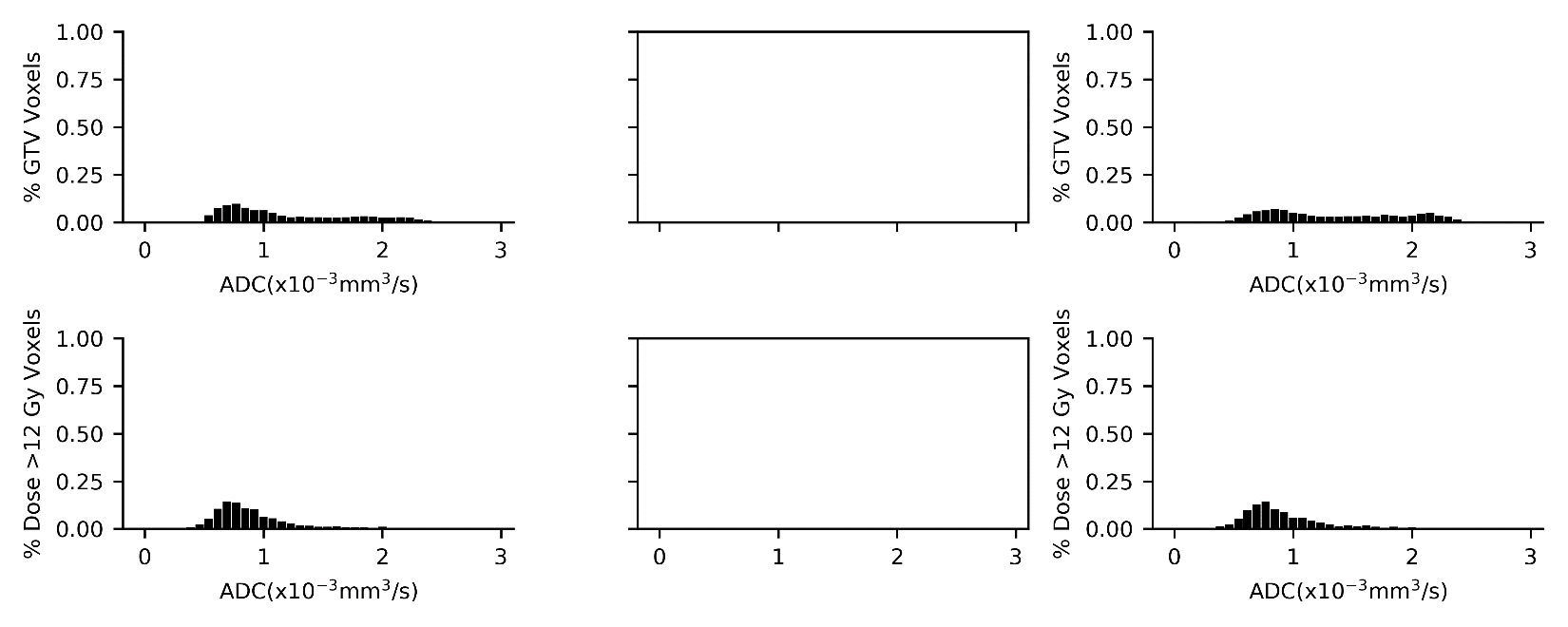
**

**
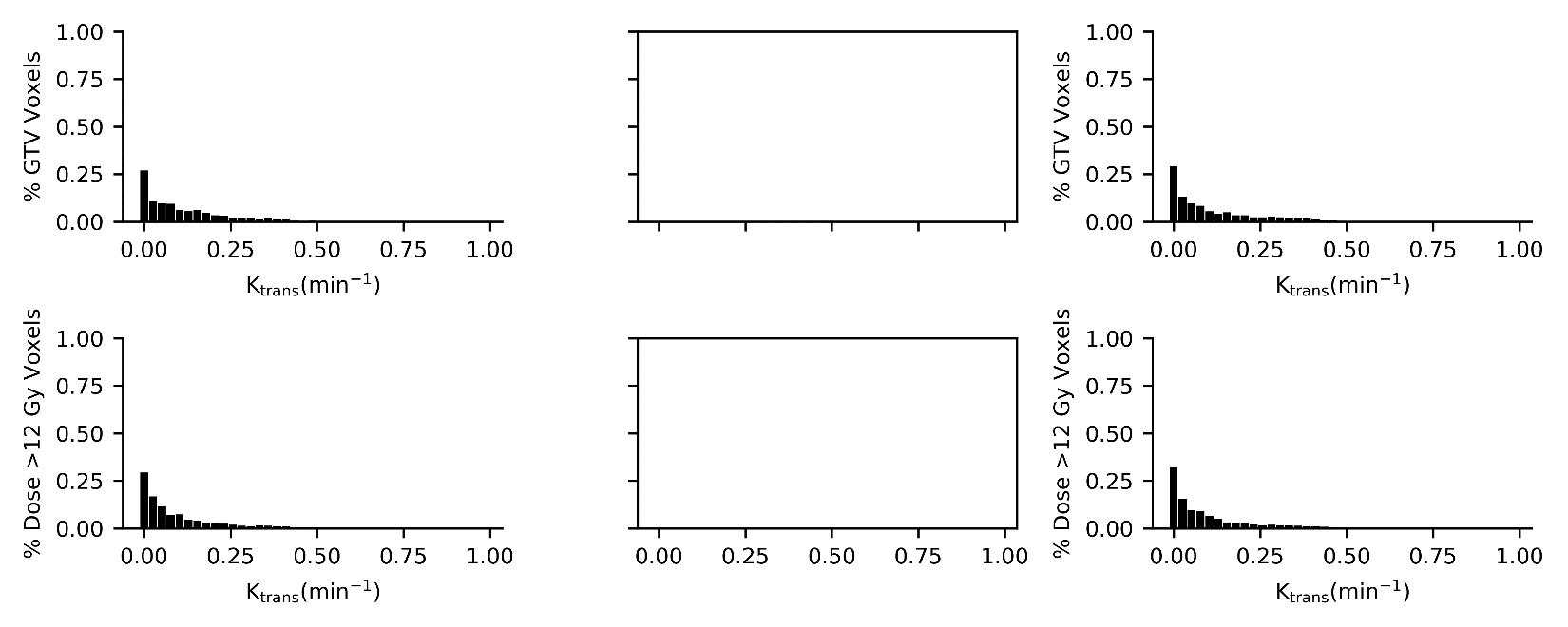
**

**
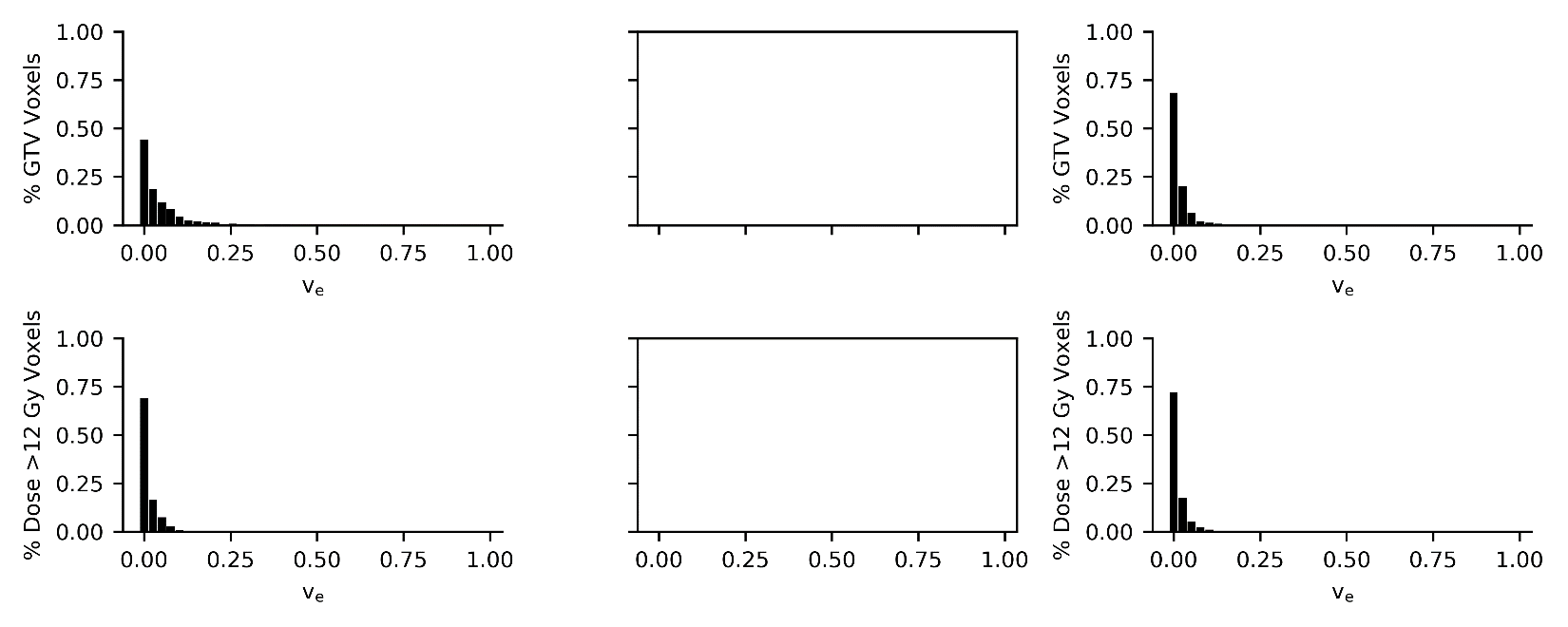
**

**Day 0 Day 3 Day 20**

**Patient 11: Metastasis 1
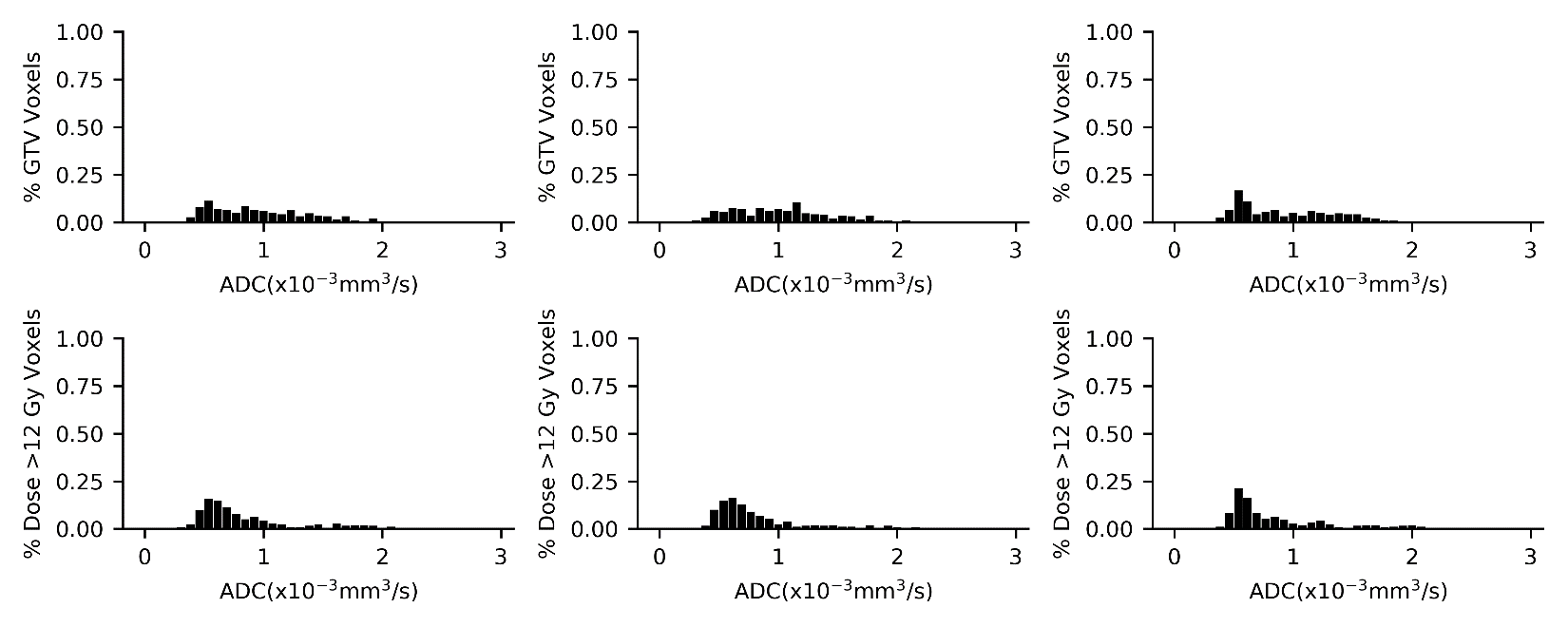
**

**
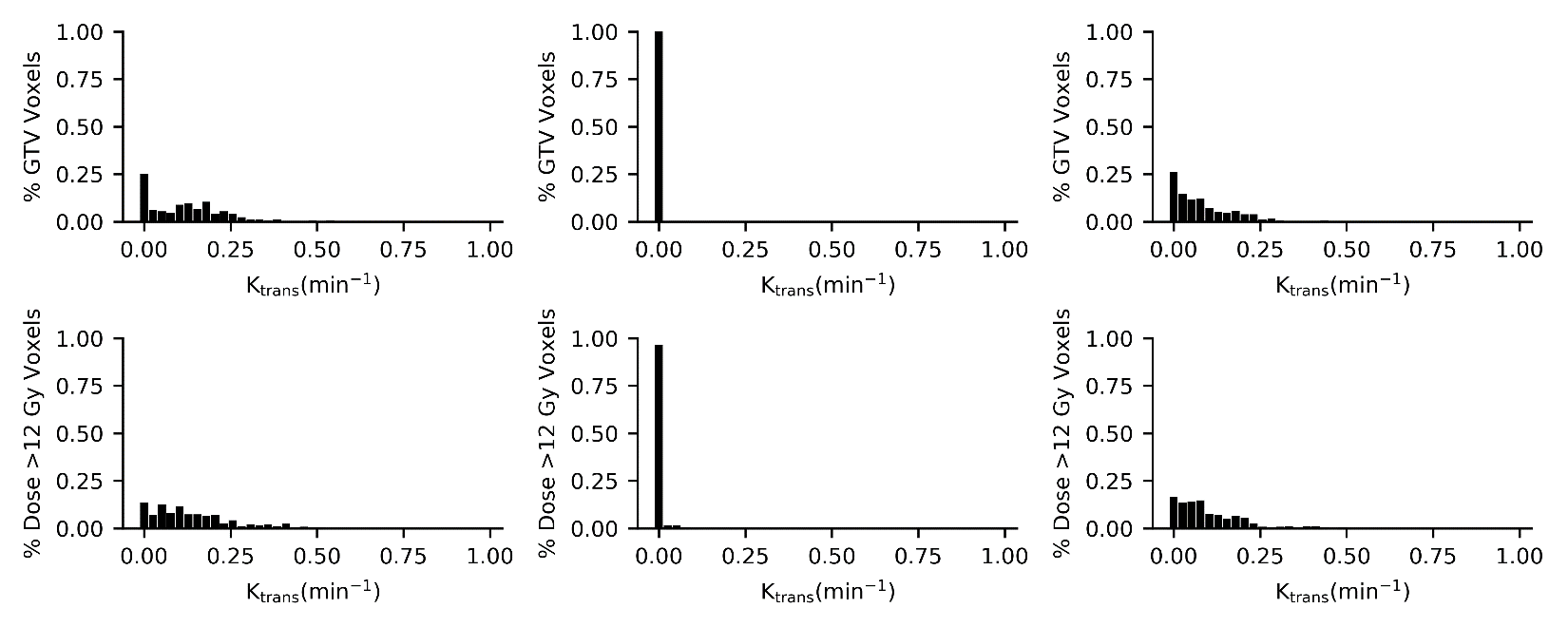
**

**
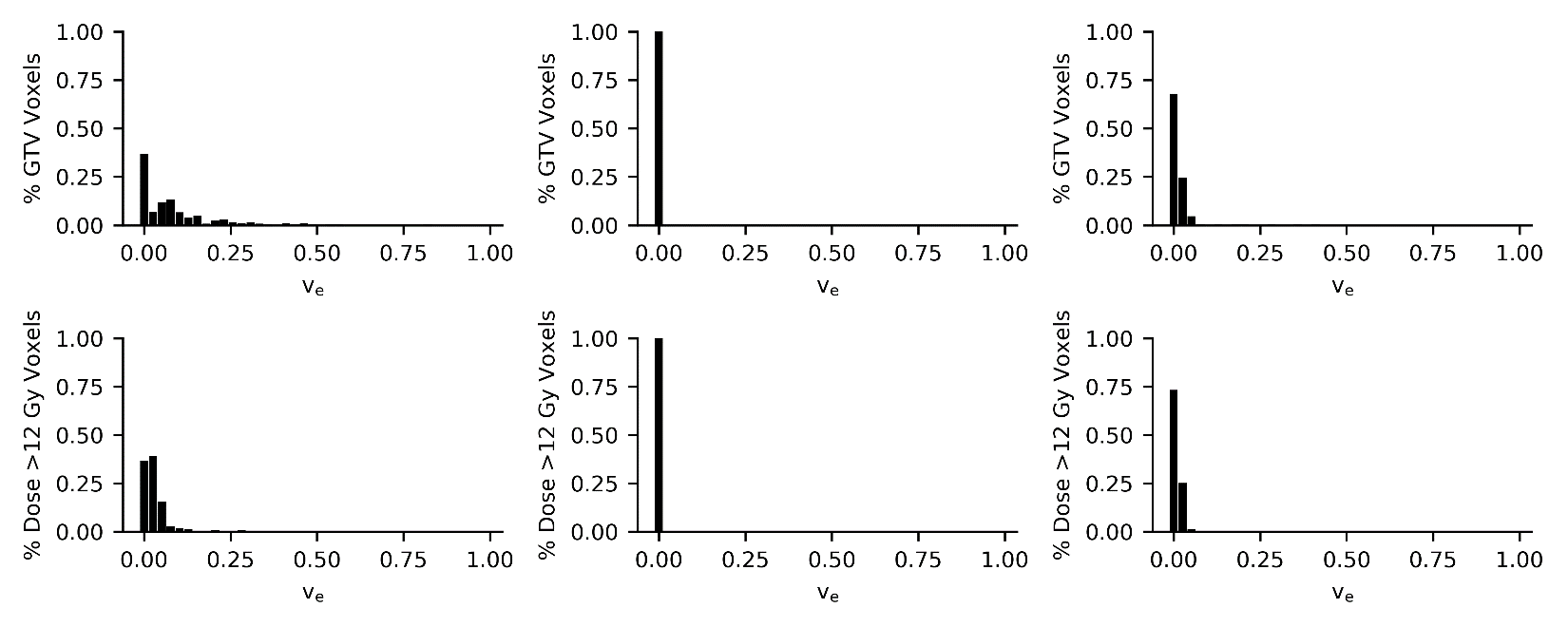
**

**Day 0 Day 3 Day 20**

**Patient 11: Metastasis 2
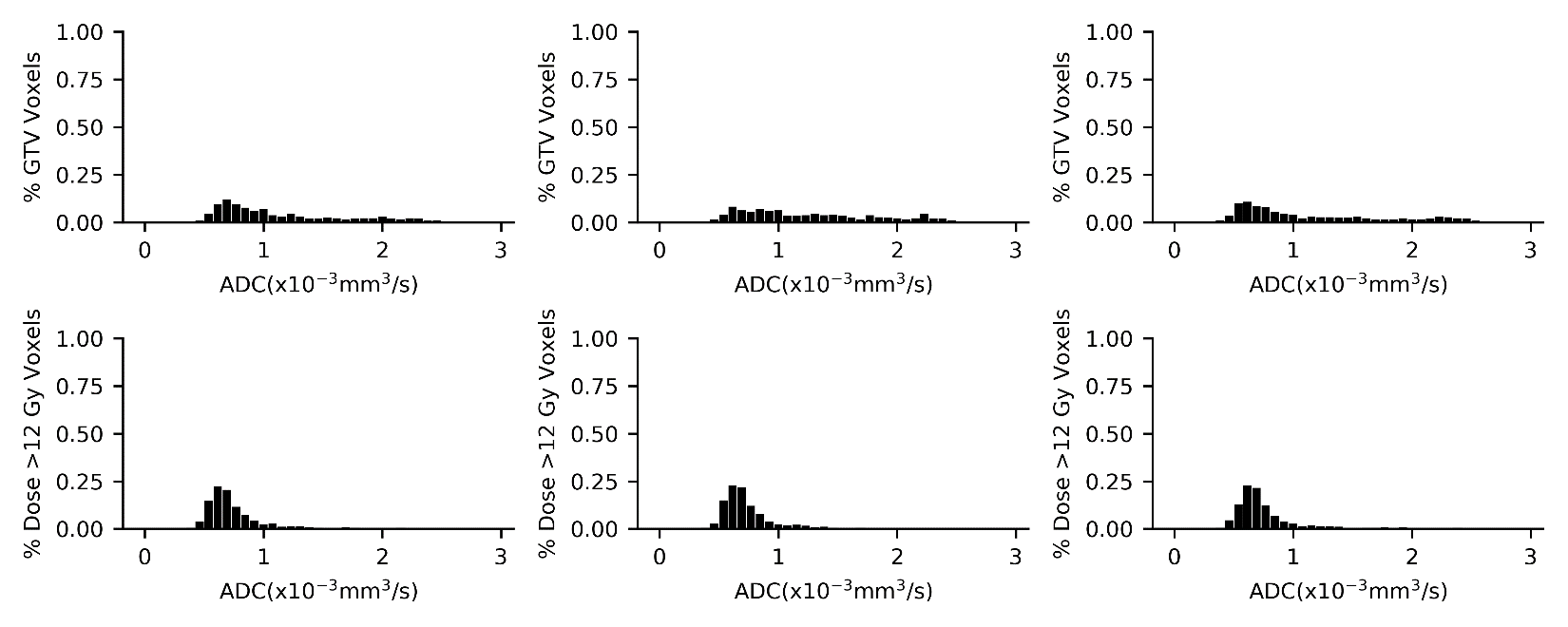
**
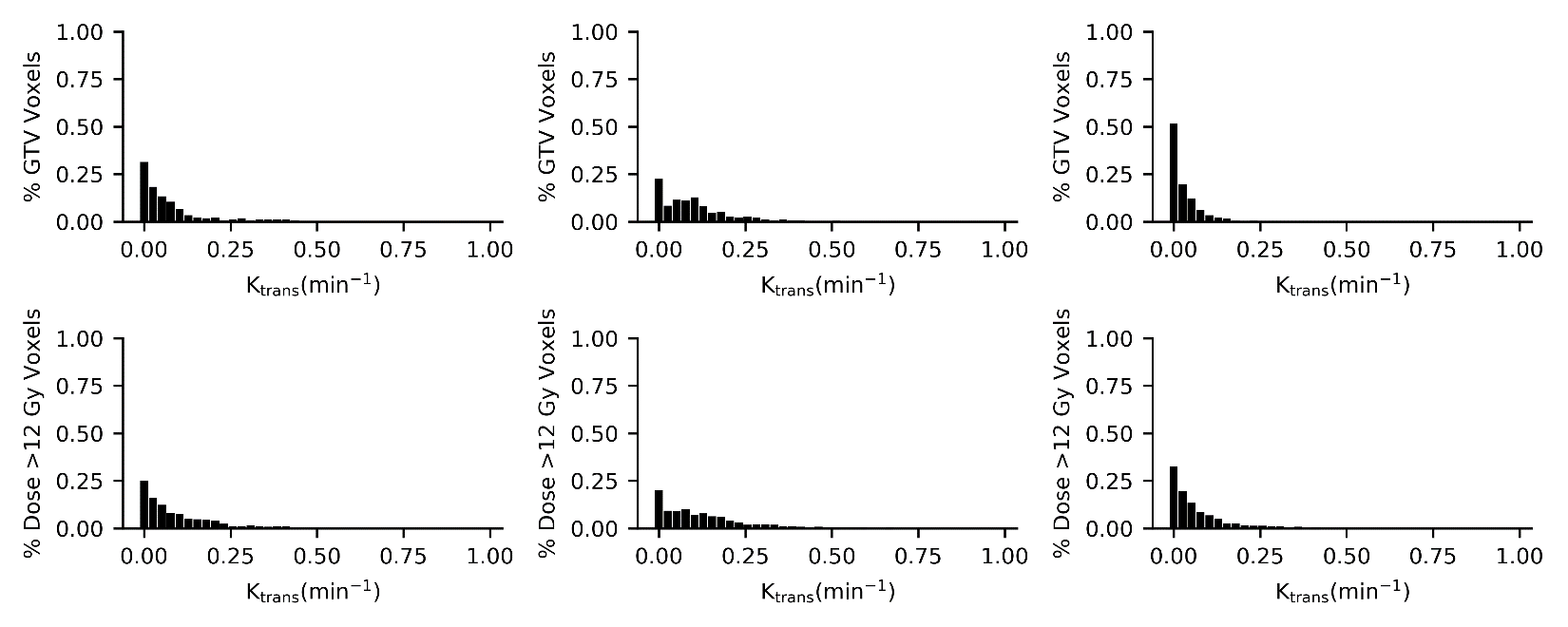

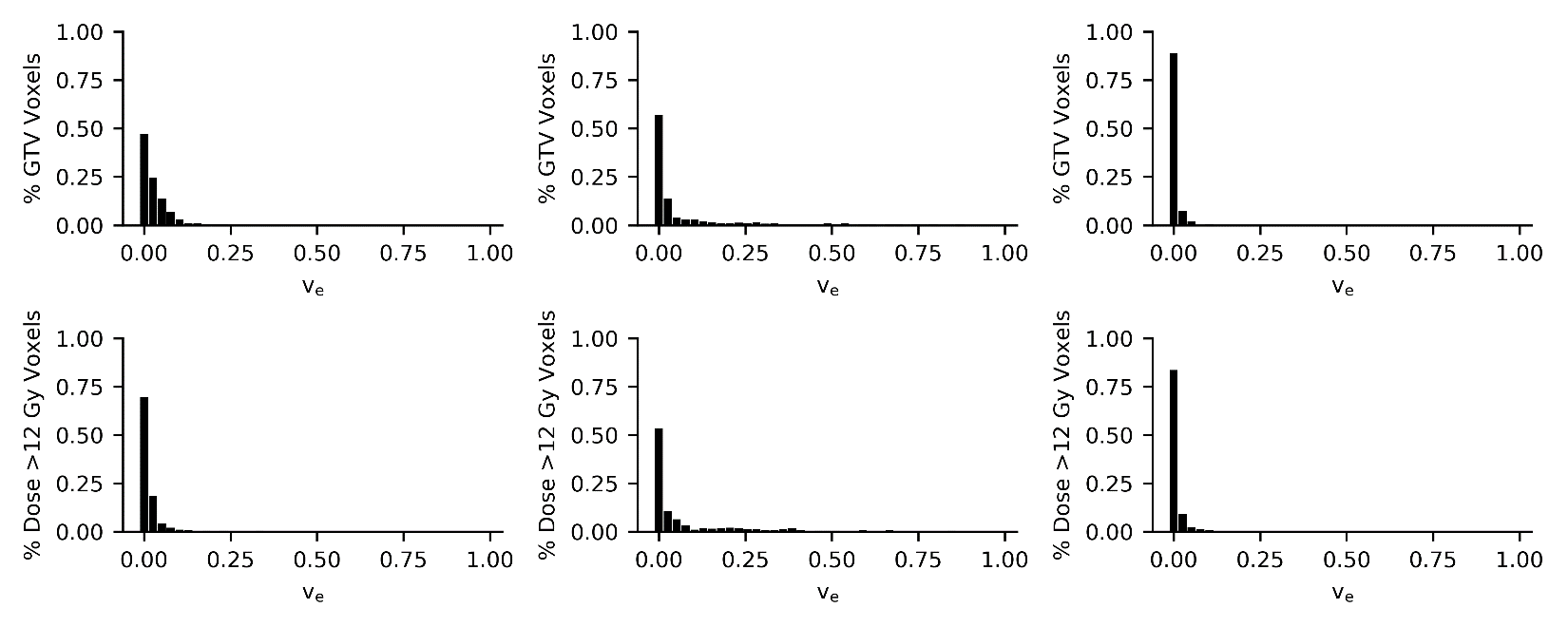


**Day 0 Day 3 Day 20**

**Patient 12: Metastasis 1
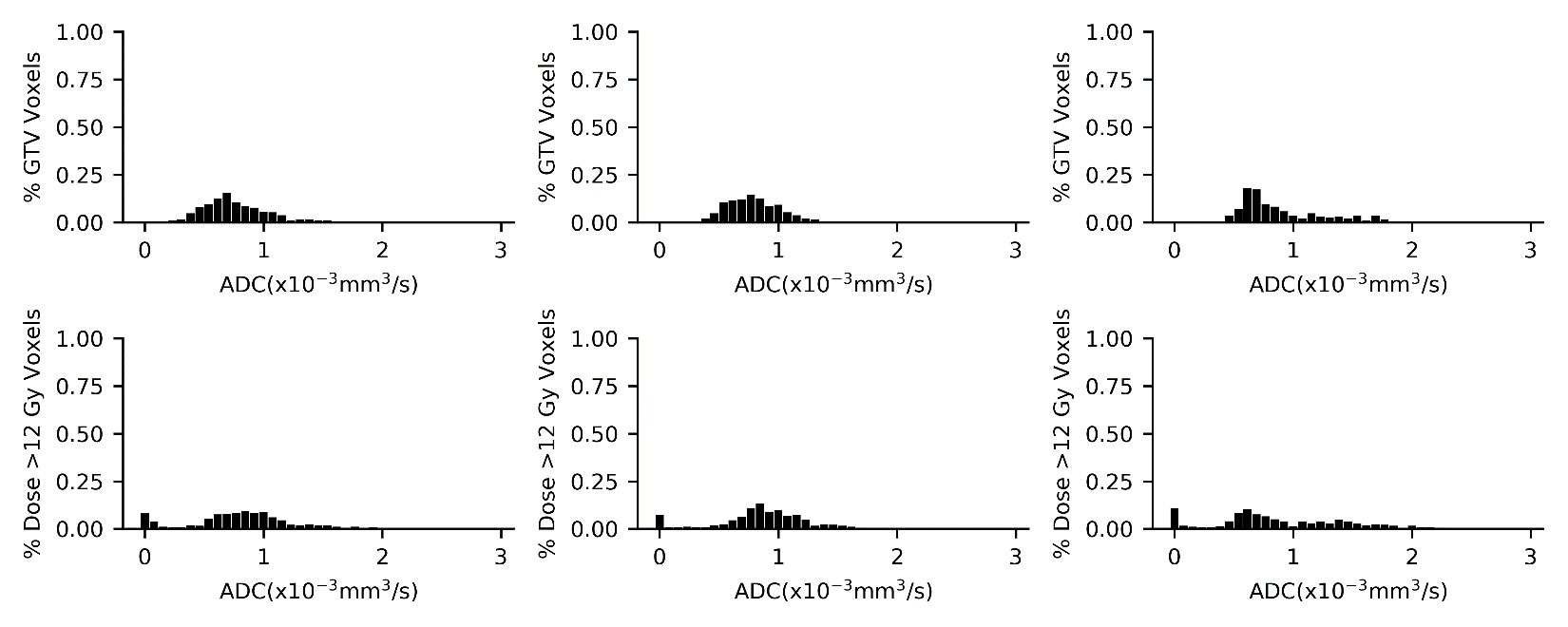
**

**
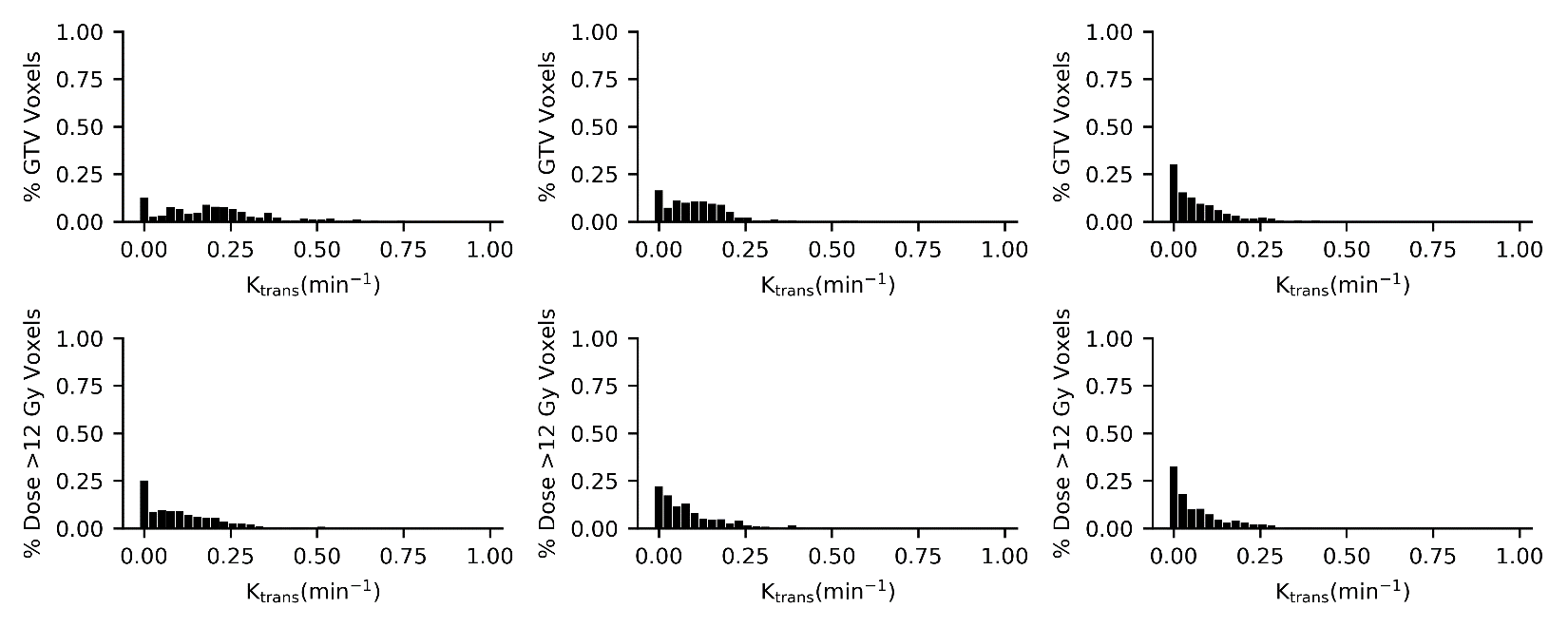
**

**
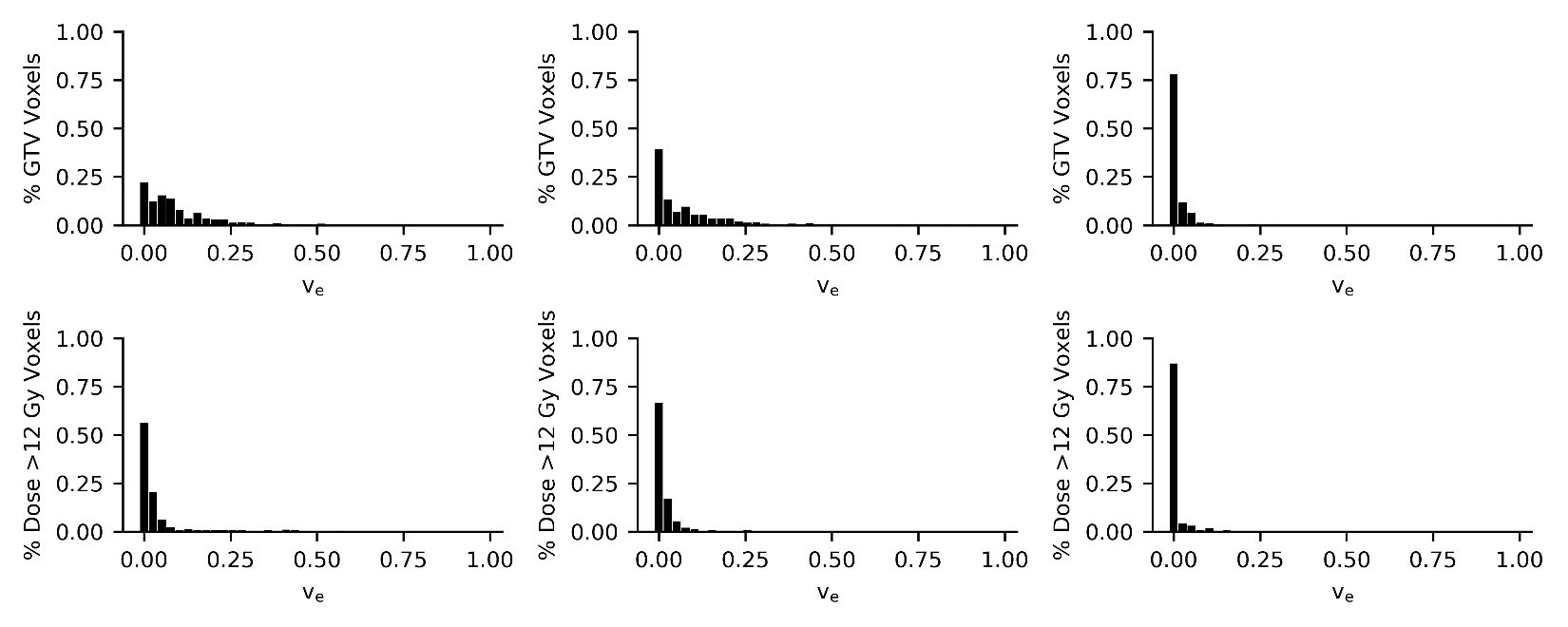
**

**Day 0 Day 3 Day 20**

**Patient 13: Metastasis 1
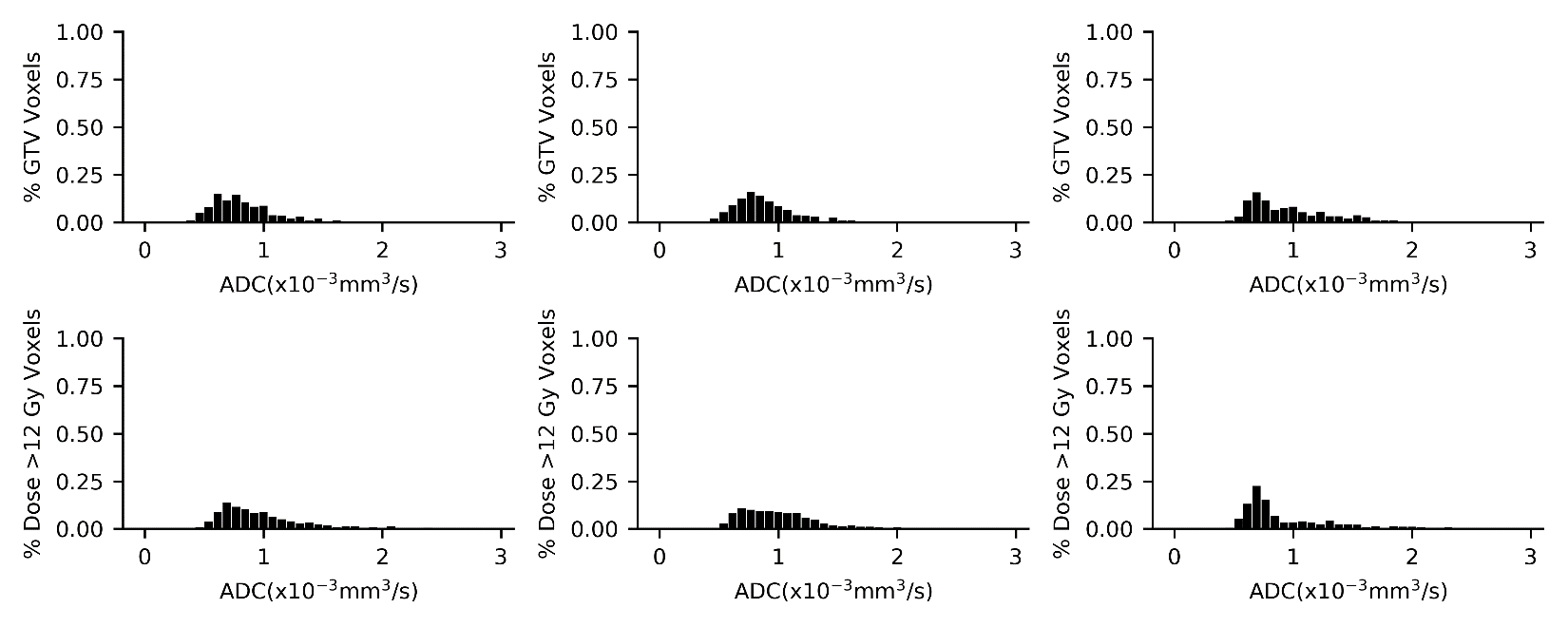
**

**
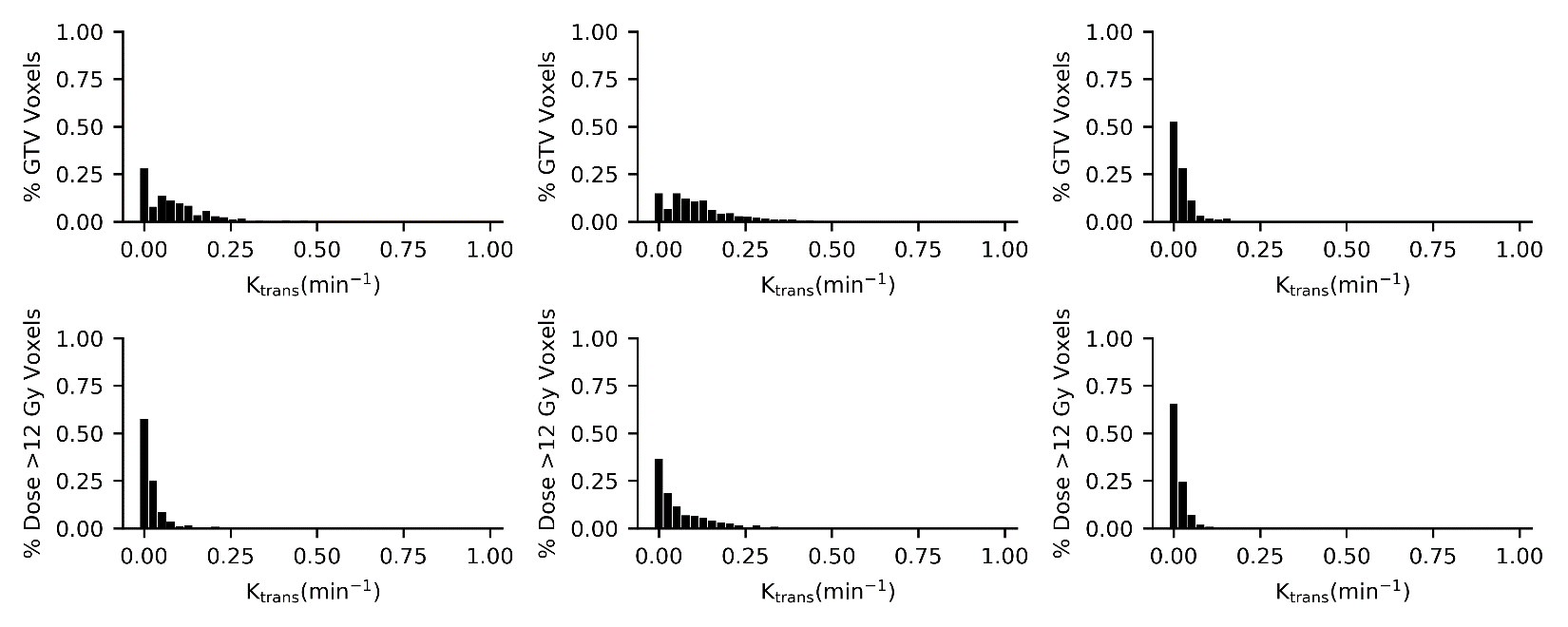
**

**
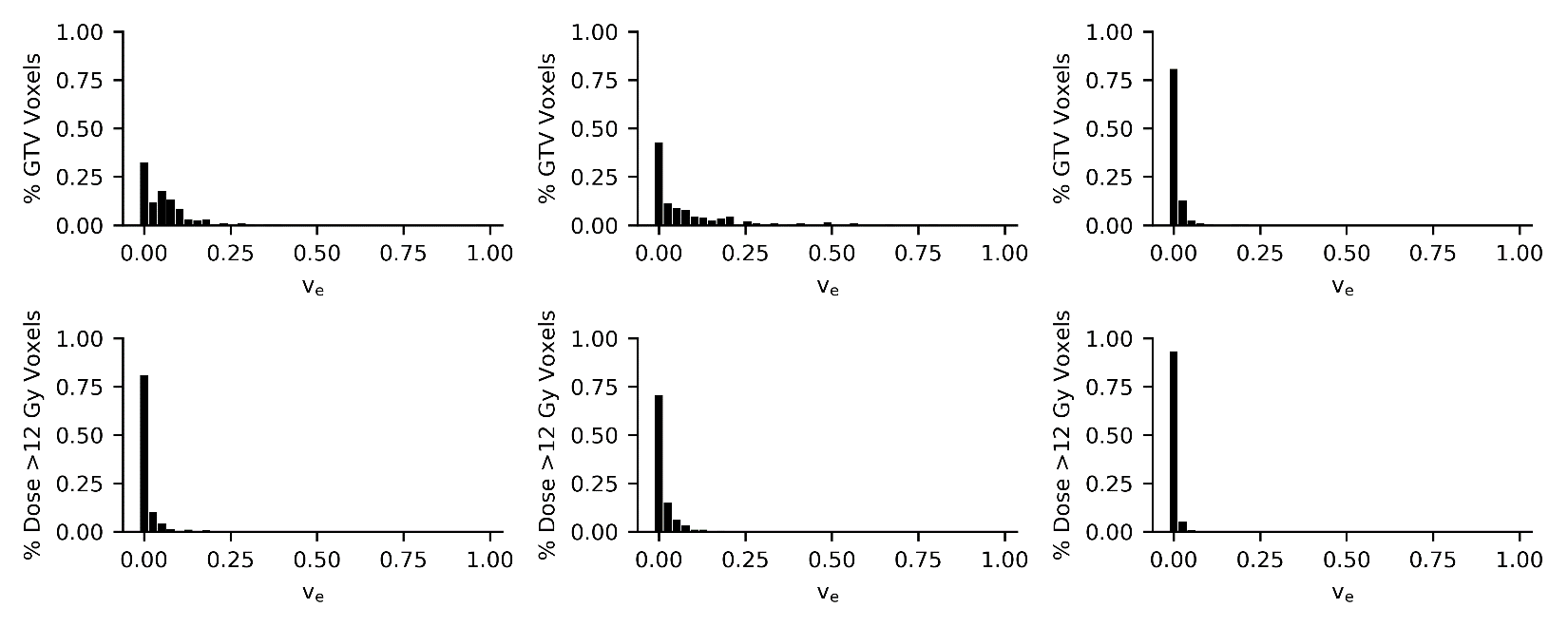
**

**Day 0 Day 3 Day 20**

**Patient 13: Metastasis 2
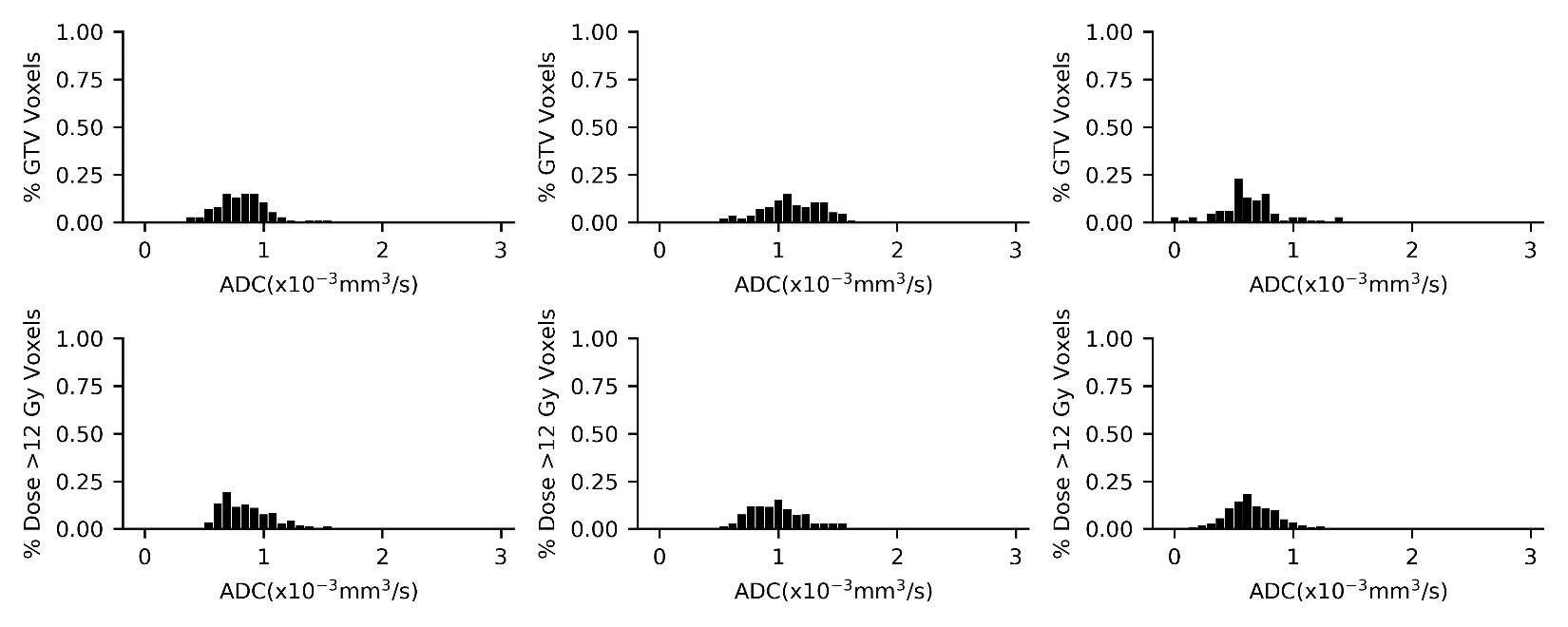
**


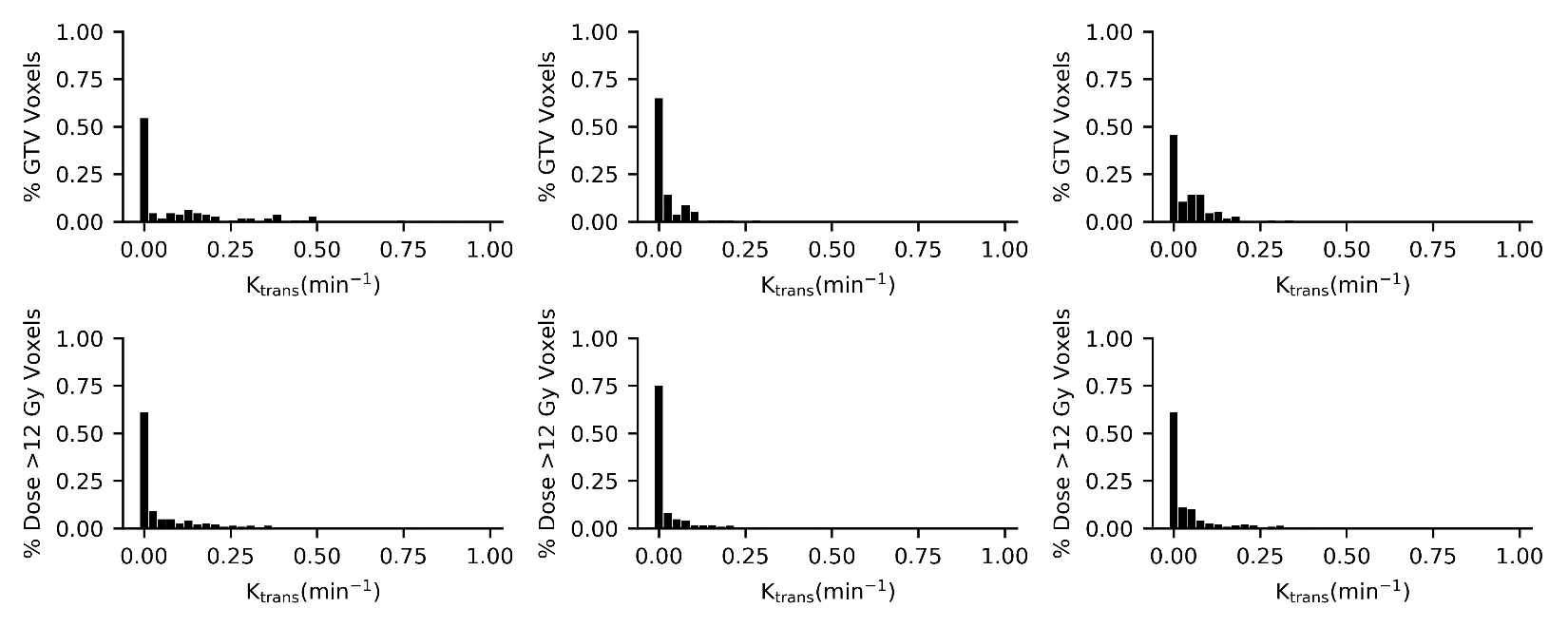


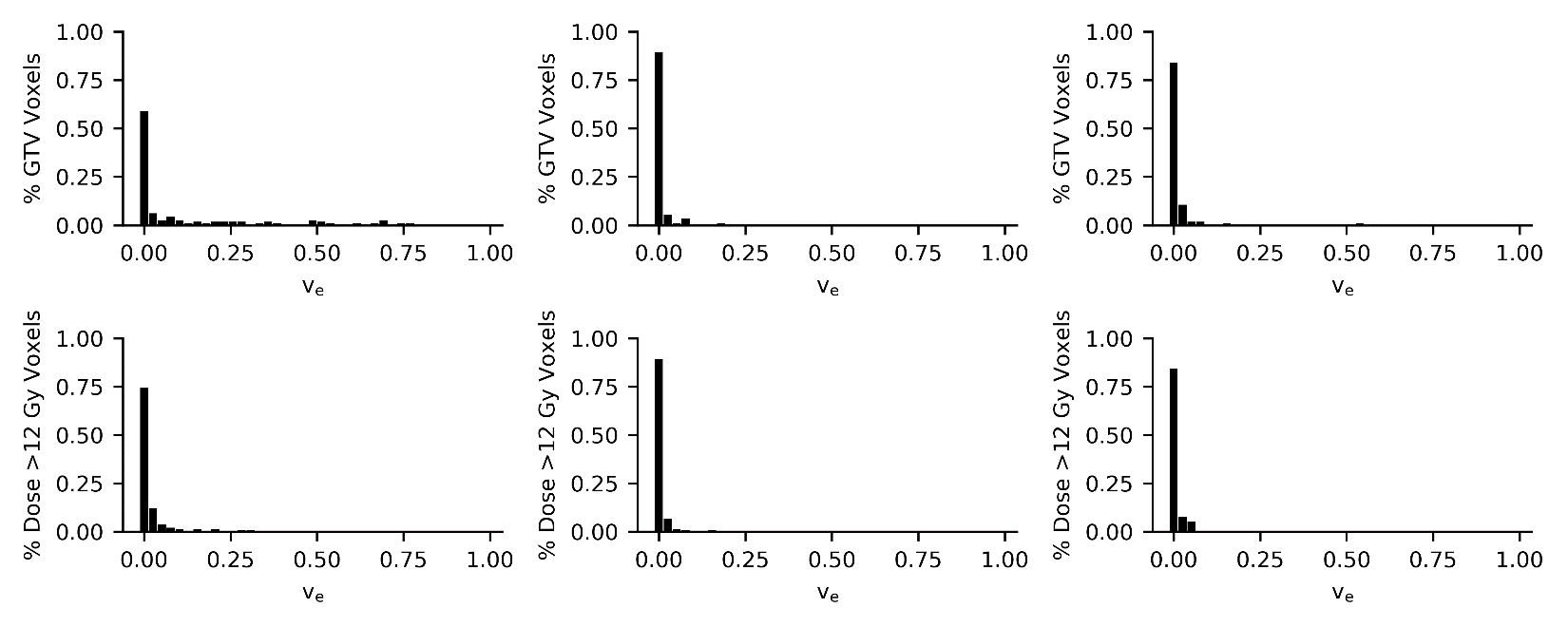


**Day 0 Day 3 Day 20**

**Patient 13: Metastasis 3
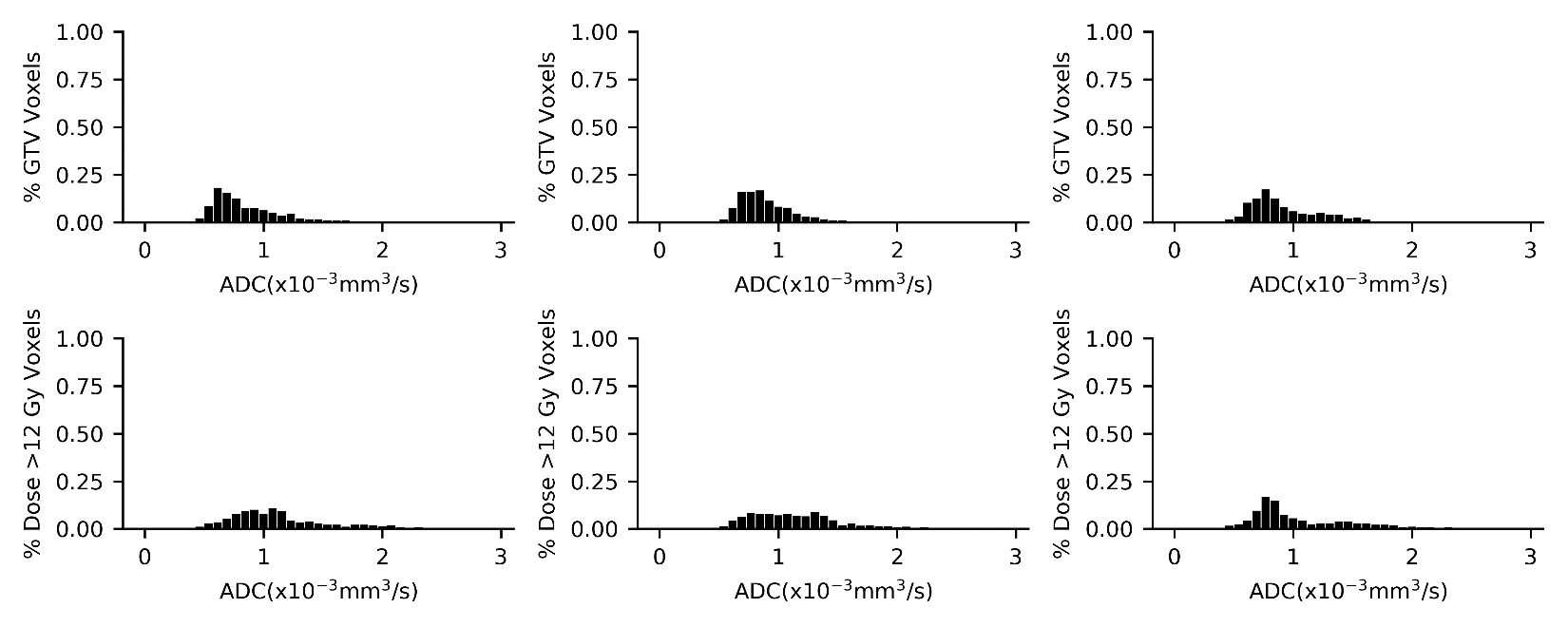

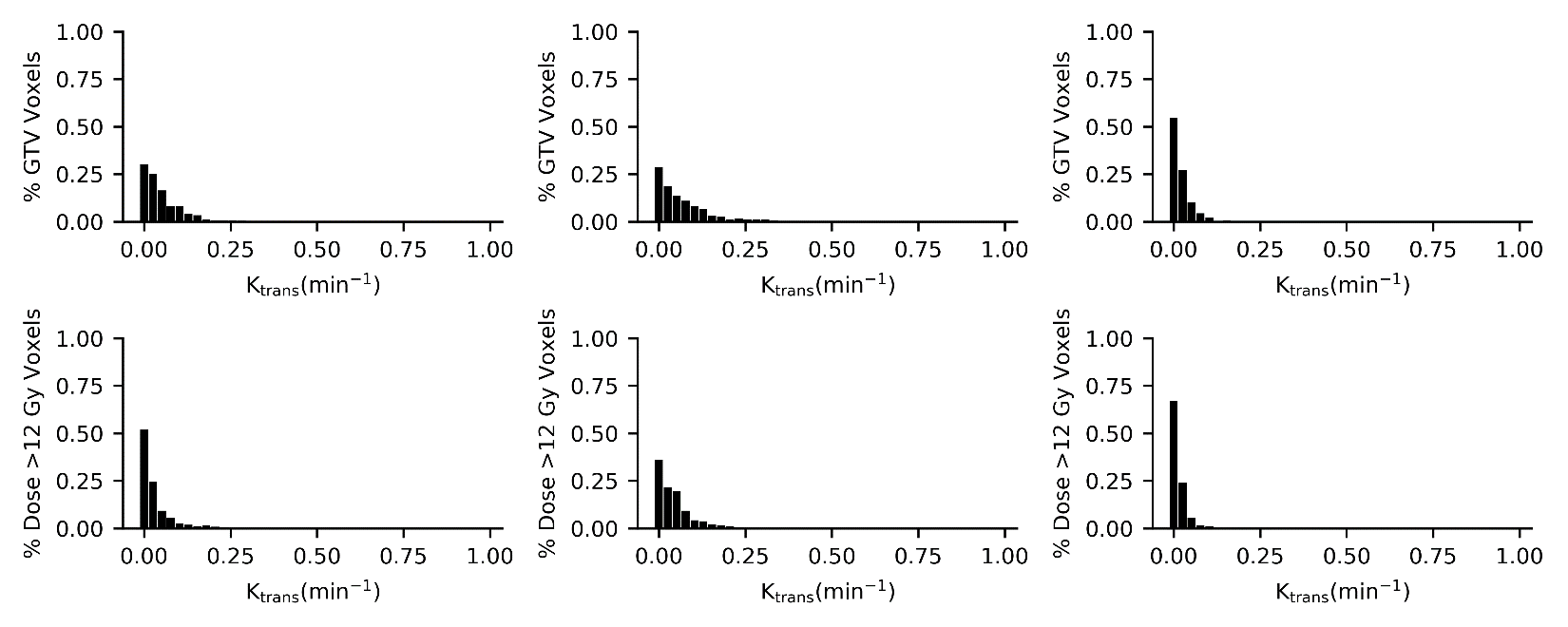
**

**
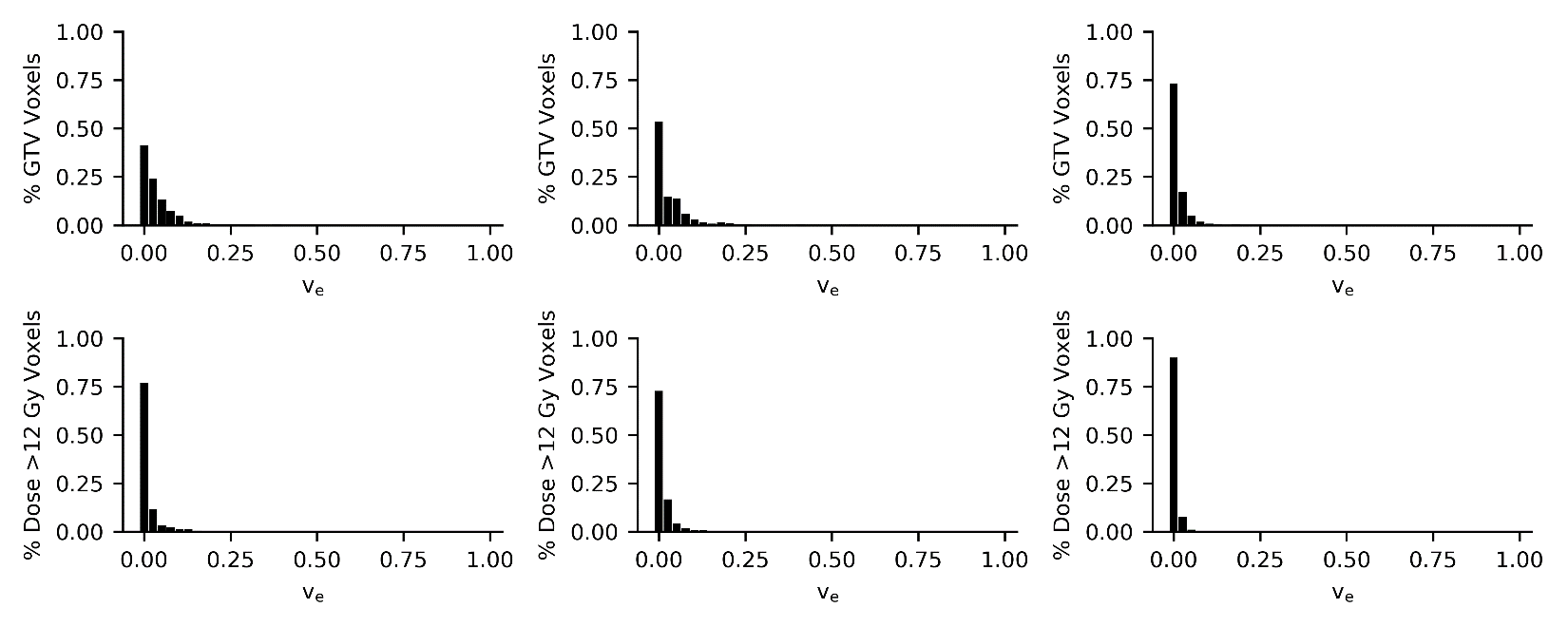
**

**Day 0 Day 3 Day 20**

**Patient 14: Metastasis 1
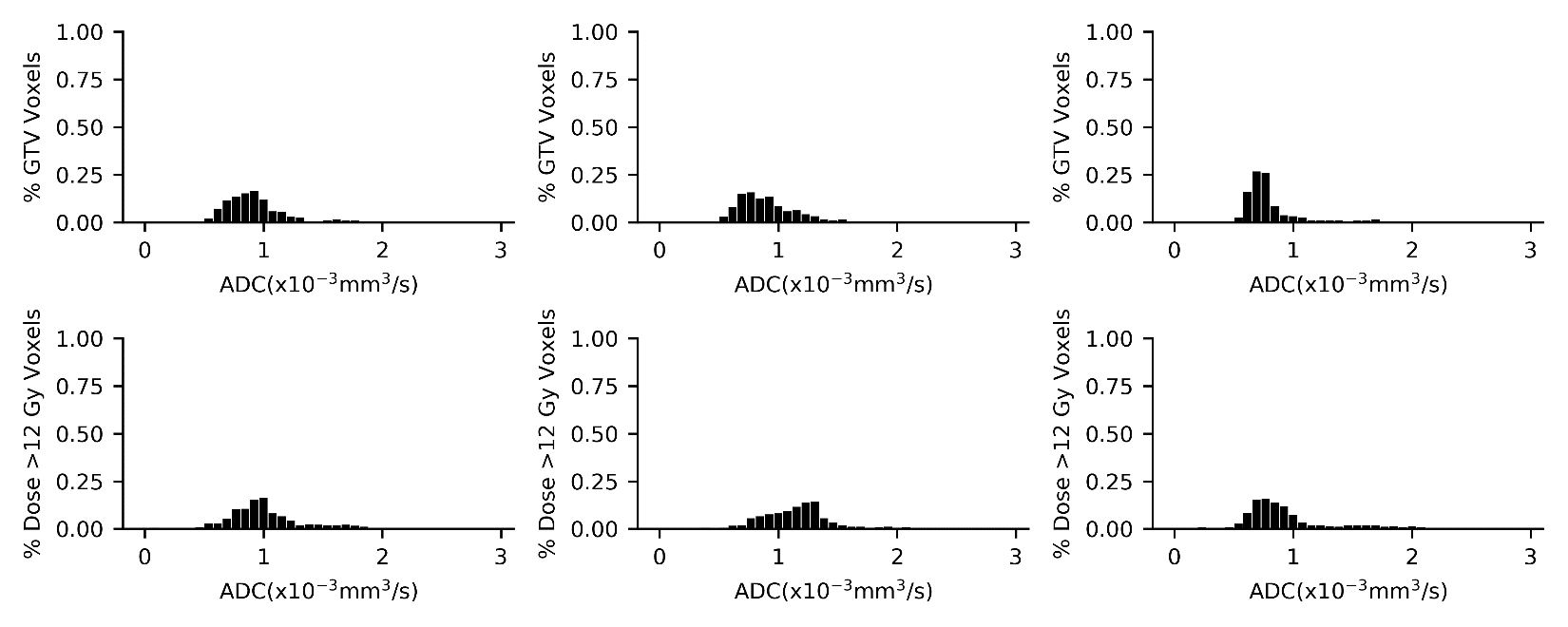
**


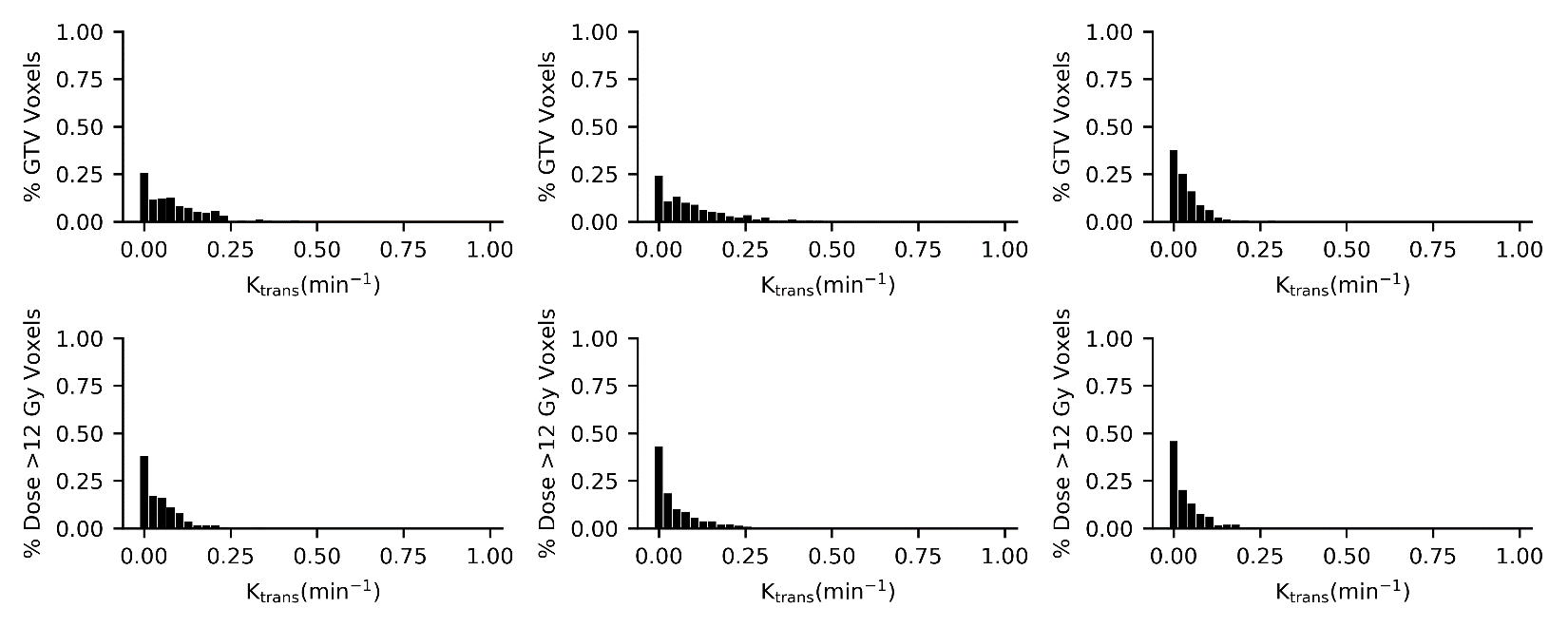


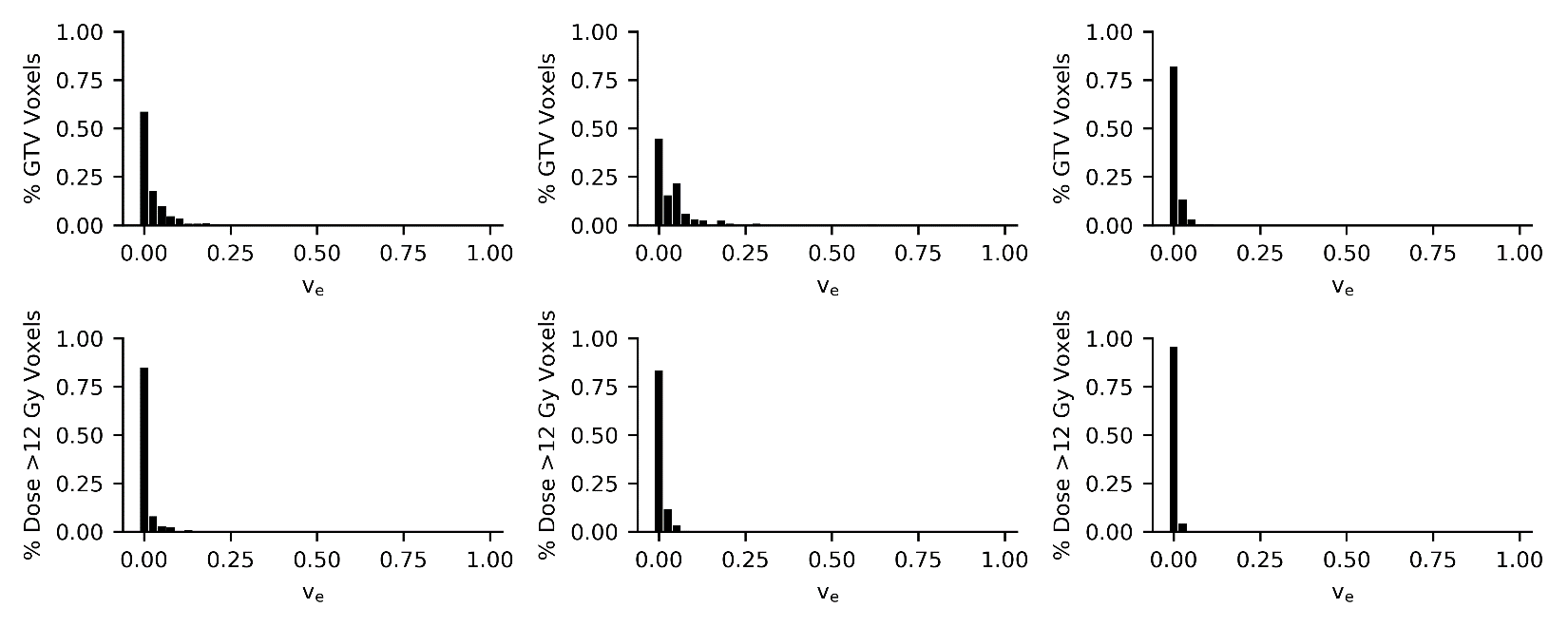


**Day 0 Day 3 Day 20**

**Patient 14: Metastasis 2
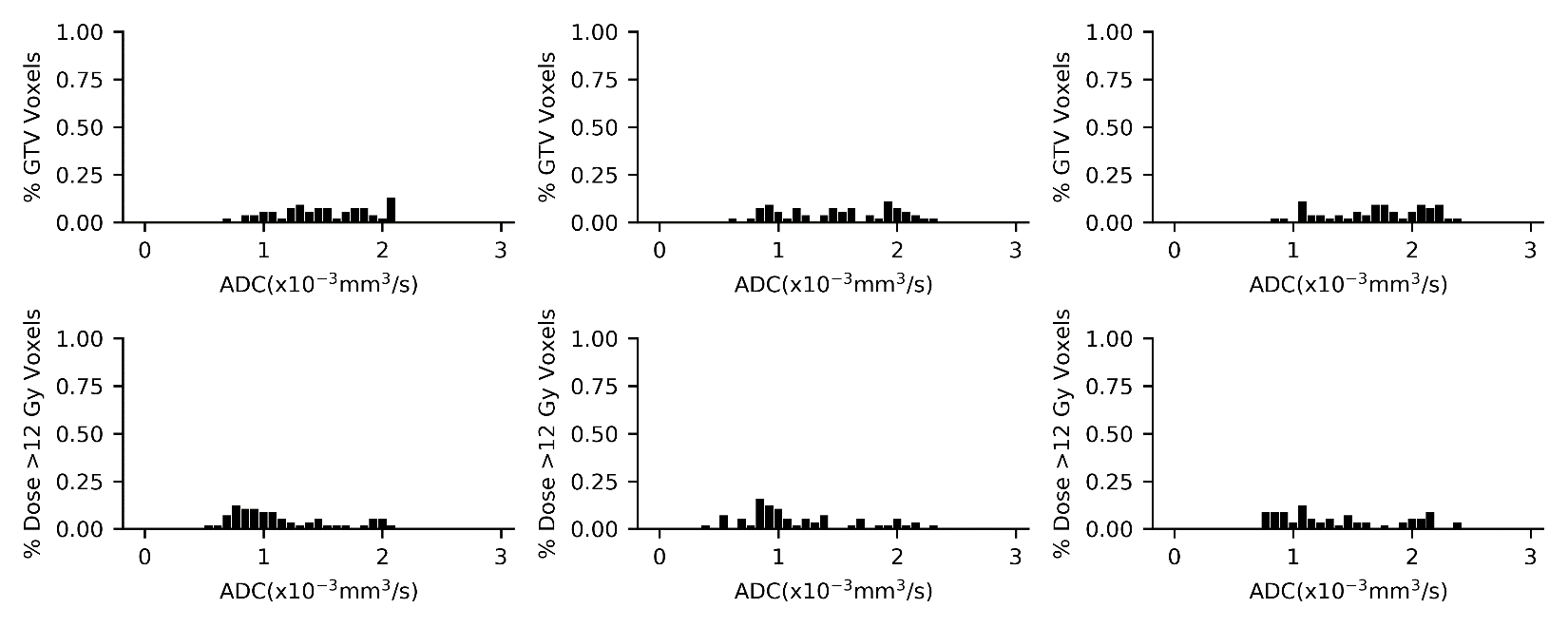
**

**
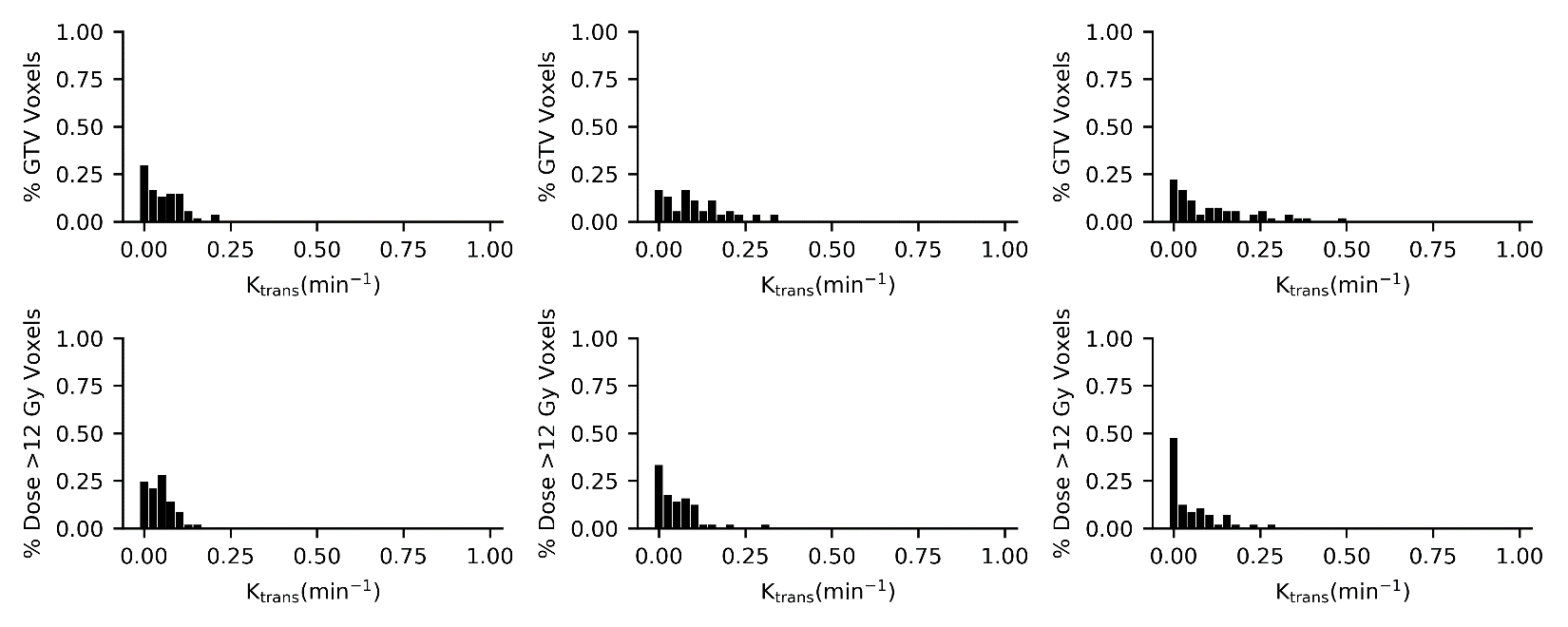
**

**
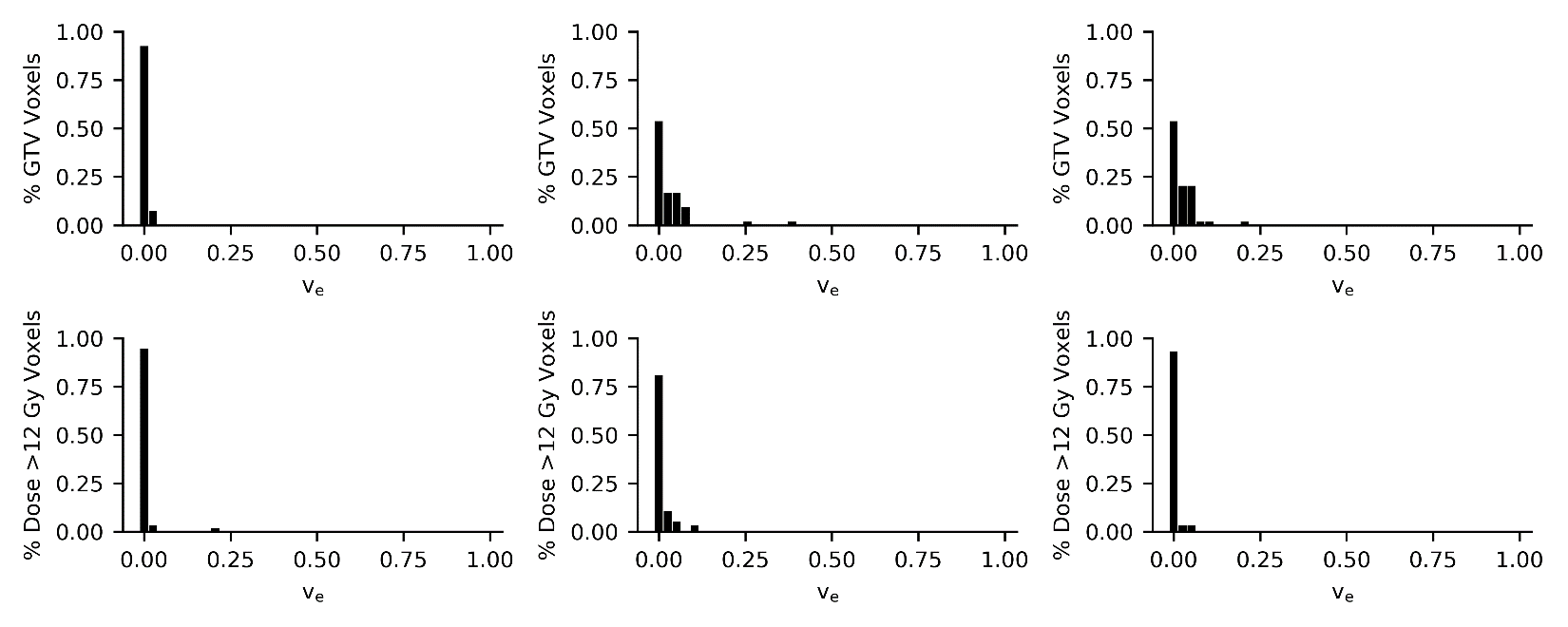
**

**Day 0 Day 3 Day 20**

**Patient 15: Metastasis 1
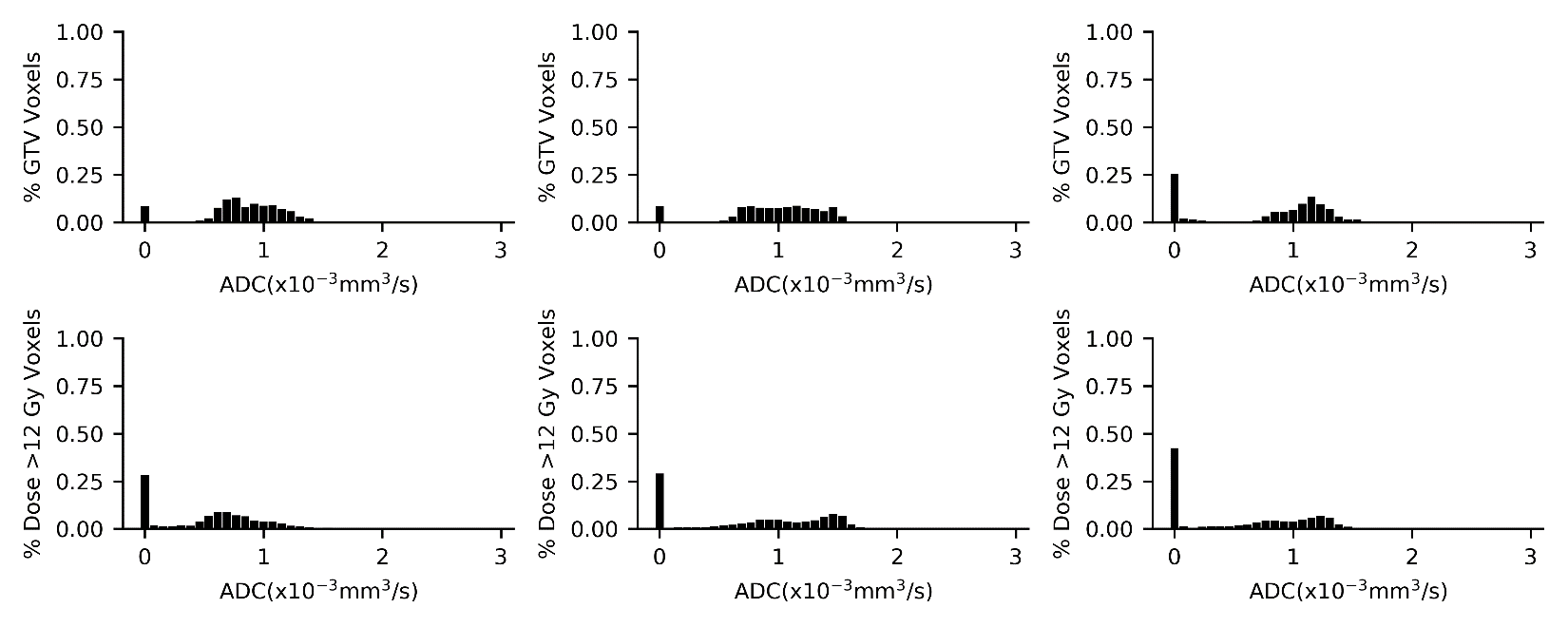
**

**
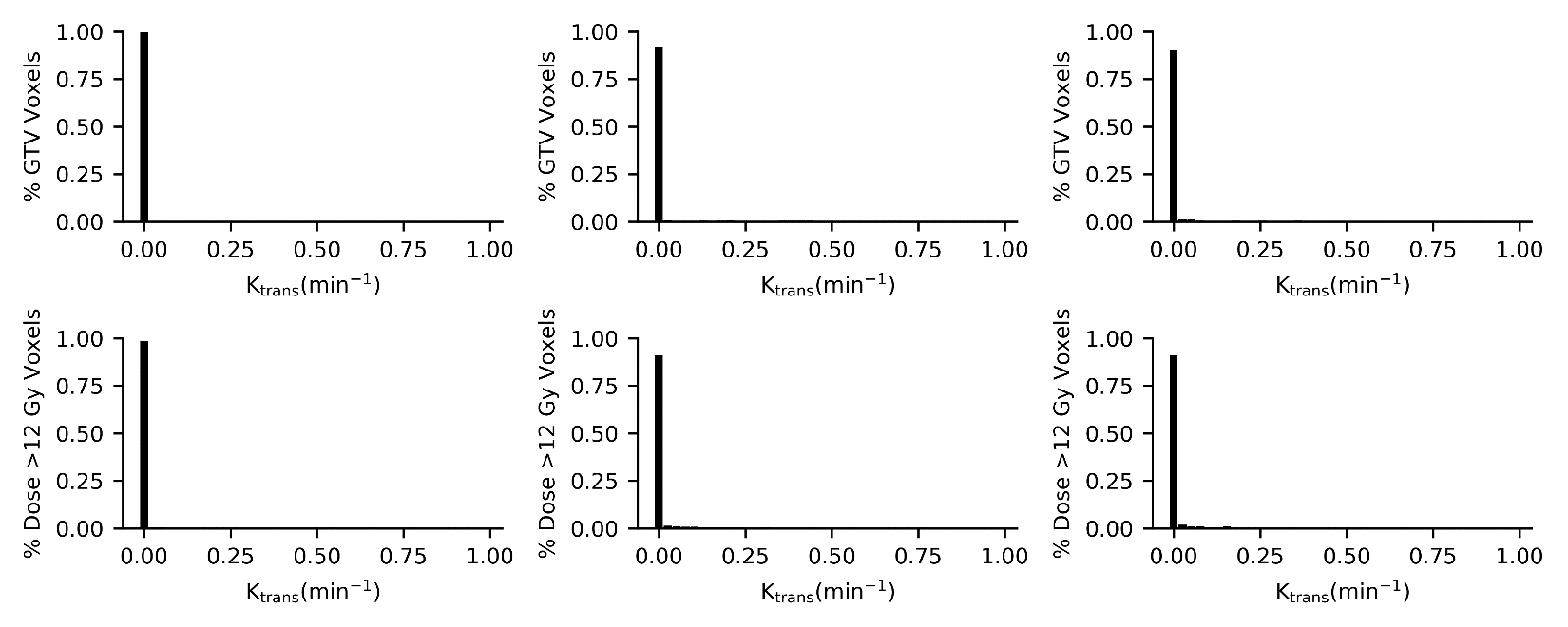
**

**
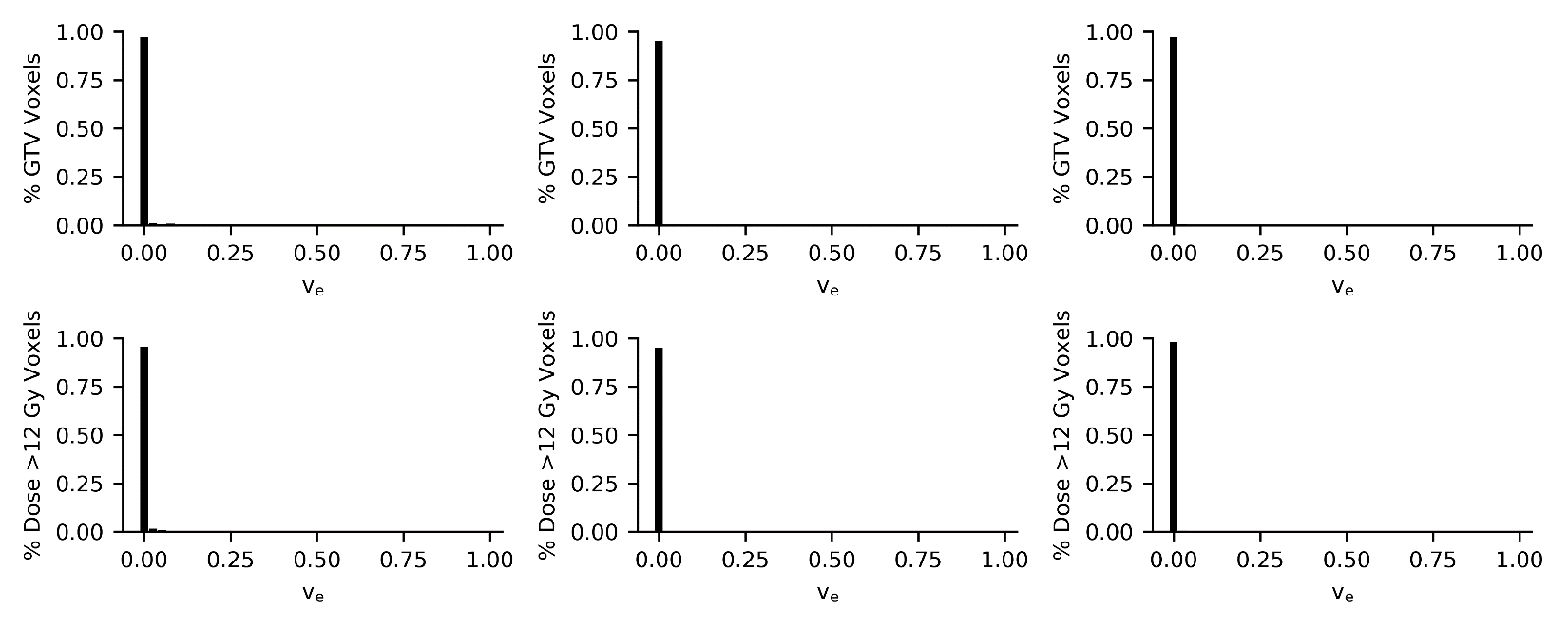
**

**Day 0 Day 3 Day 20**

**Patient 15: Metastasis 2
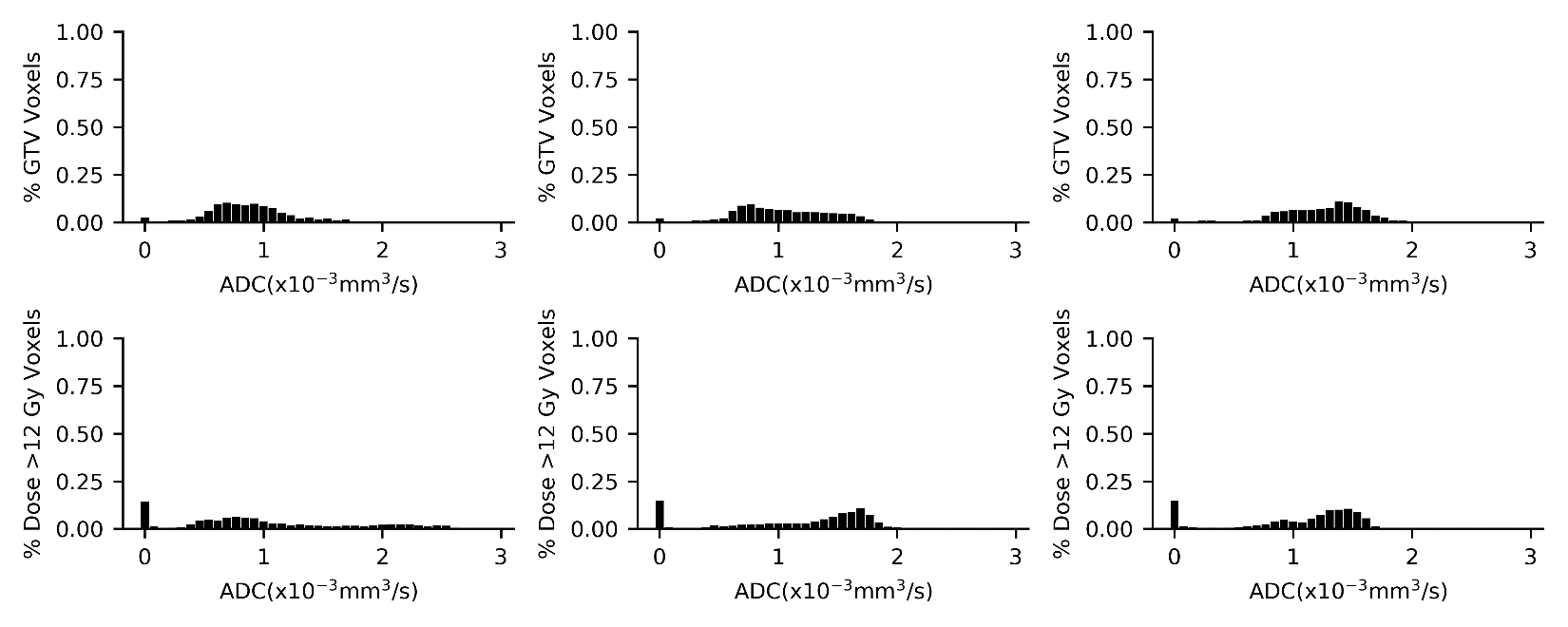
**


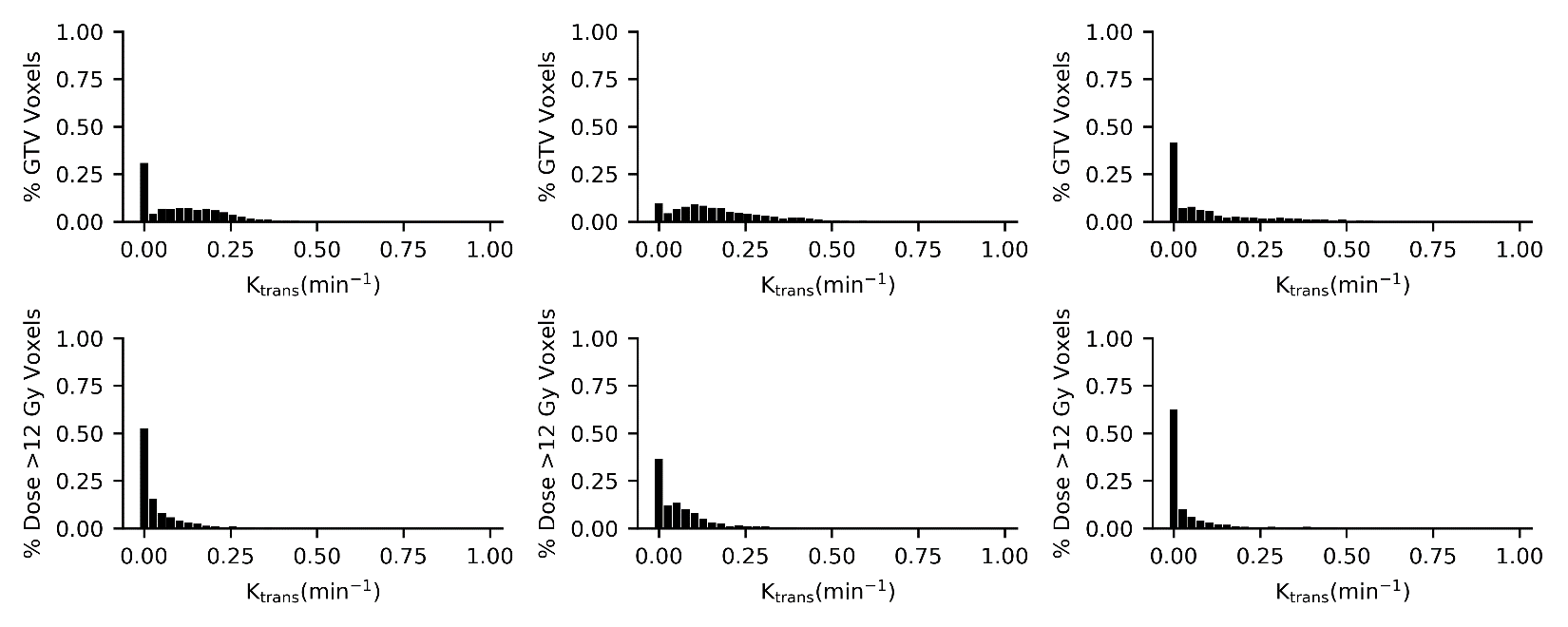


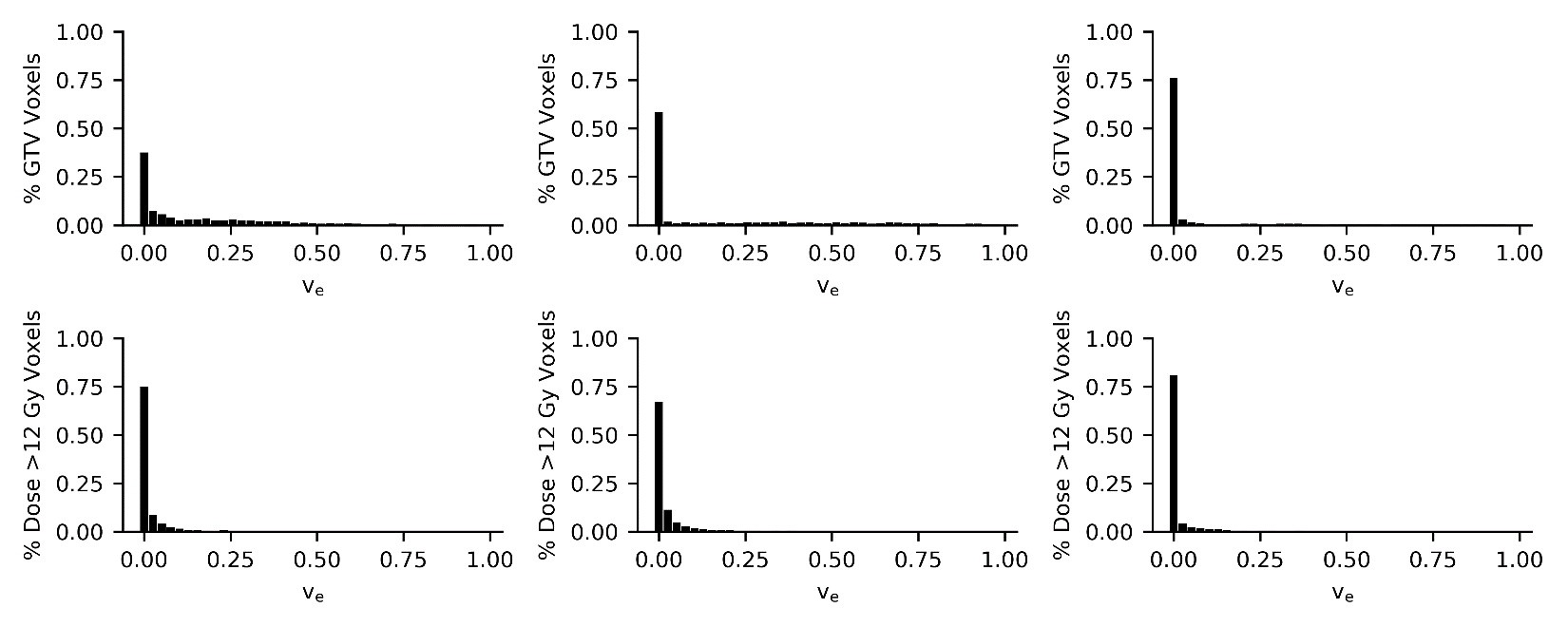


**Day 0 Day 3 Day 20**

**Patient 16: Metastasis 1
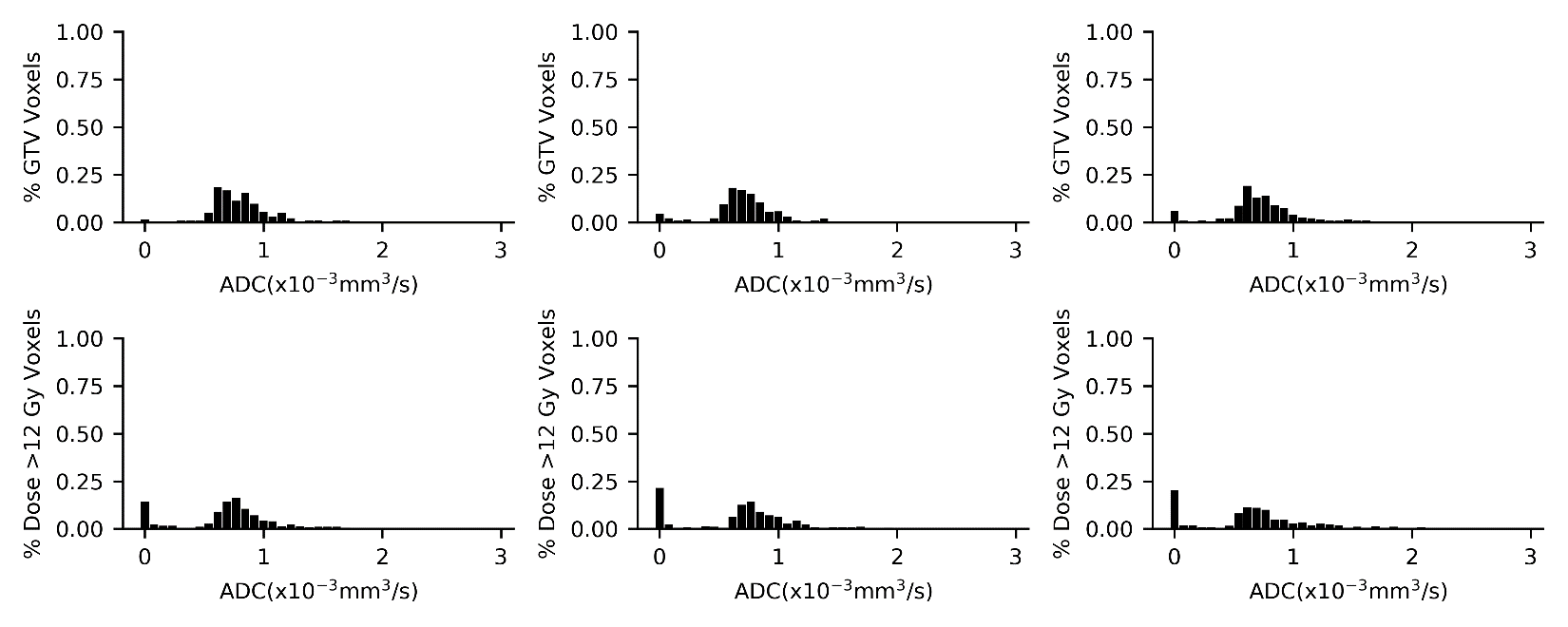
**

**
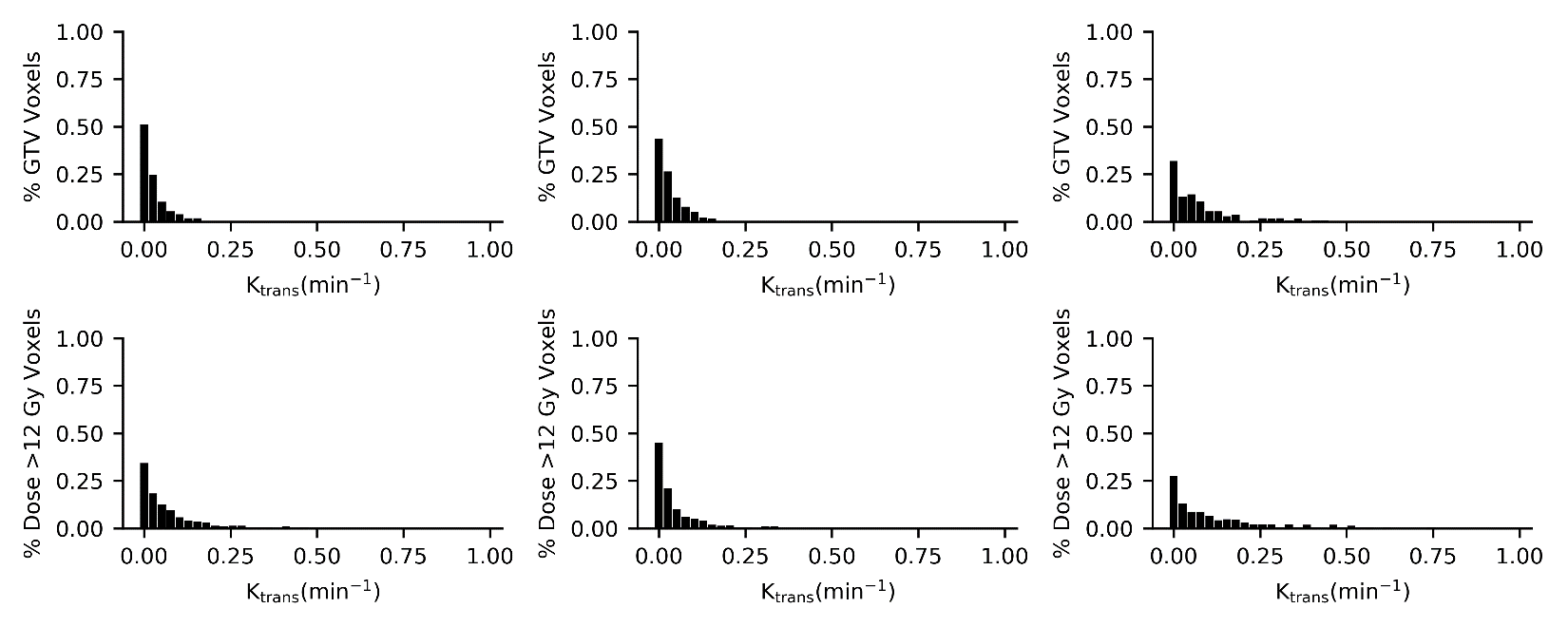
**

**
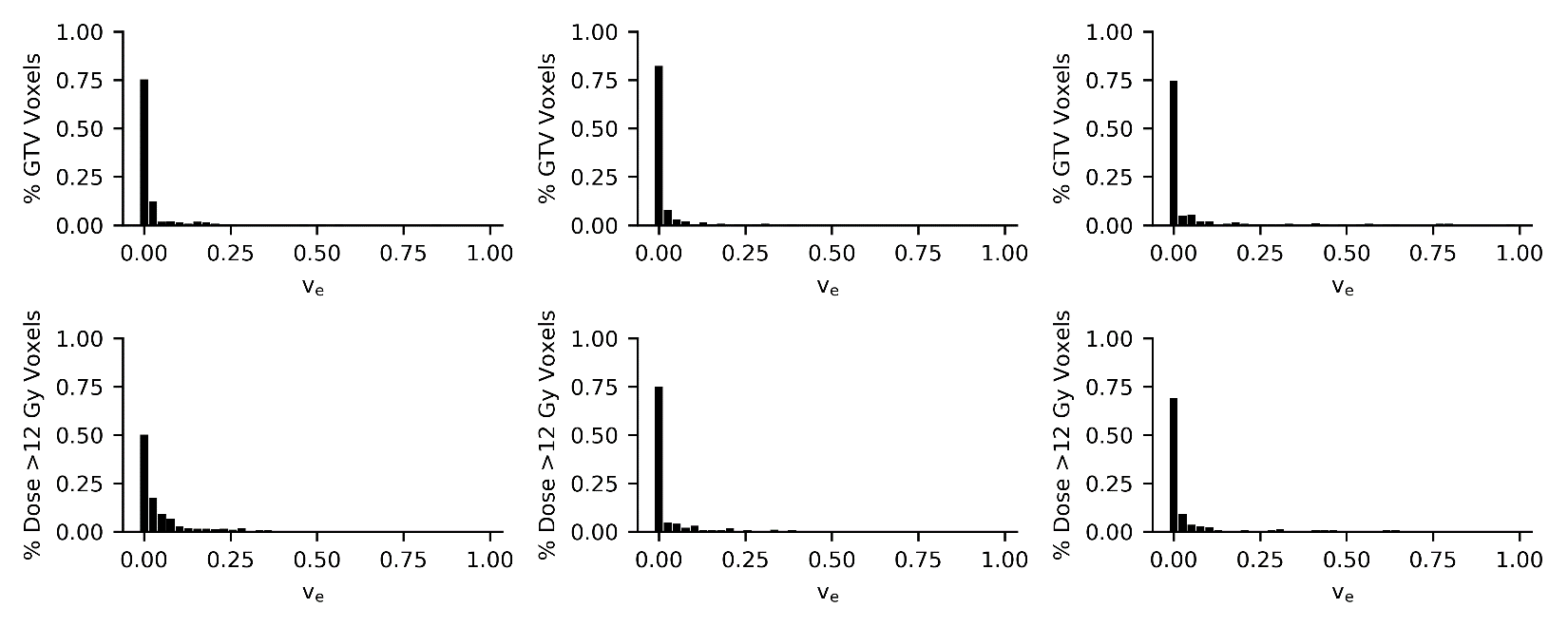
**

**Day 0 Day 3 Day 20**

**Patient 16: Metastasis 2
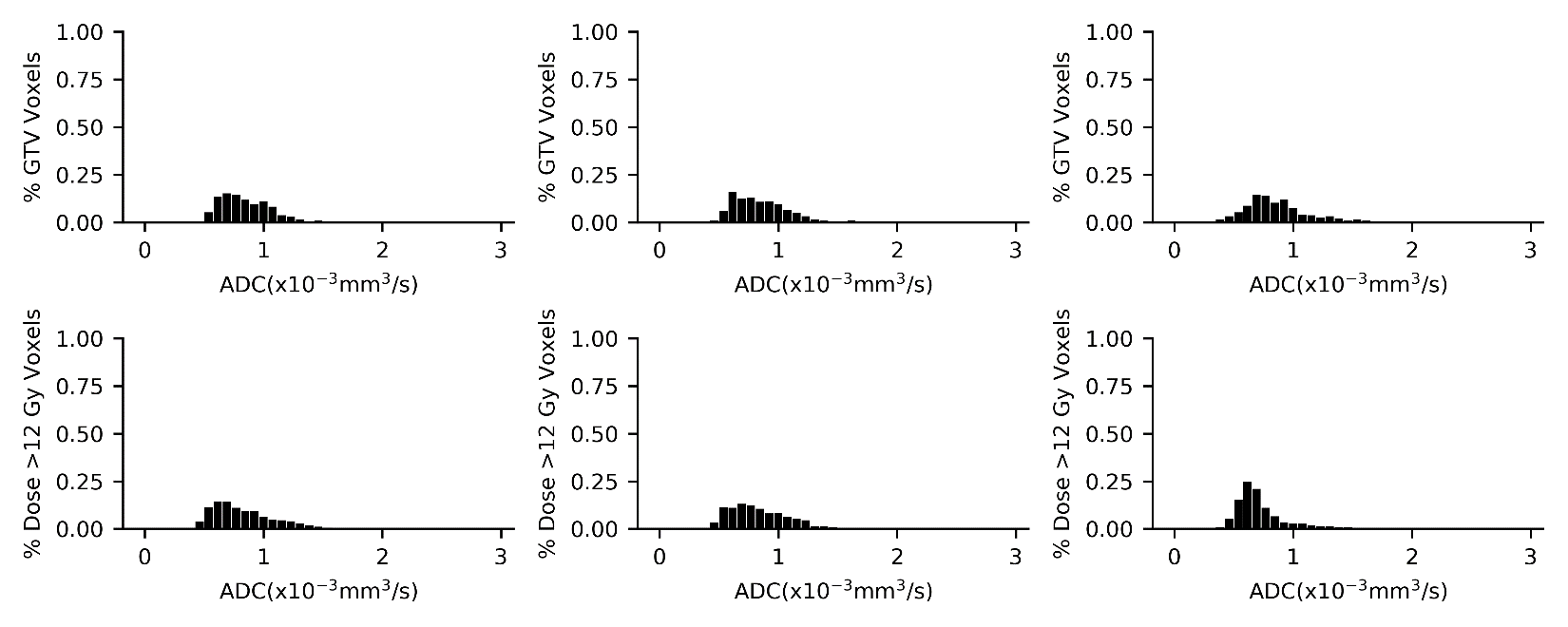
**


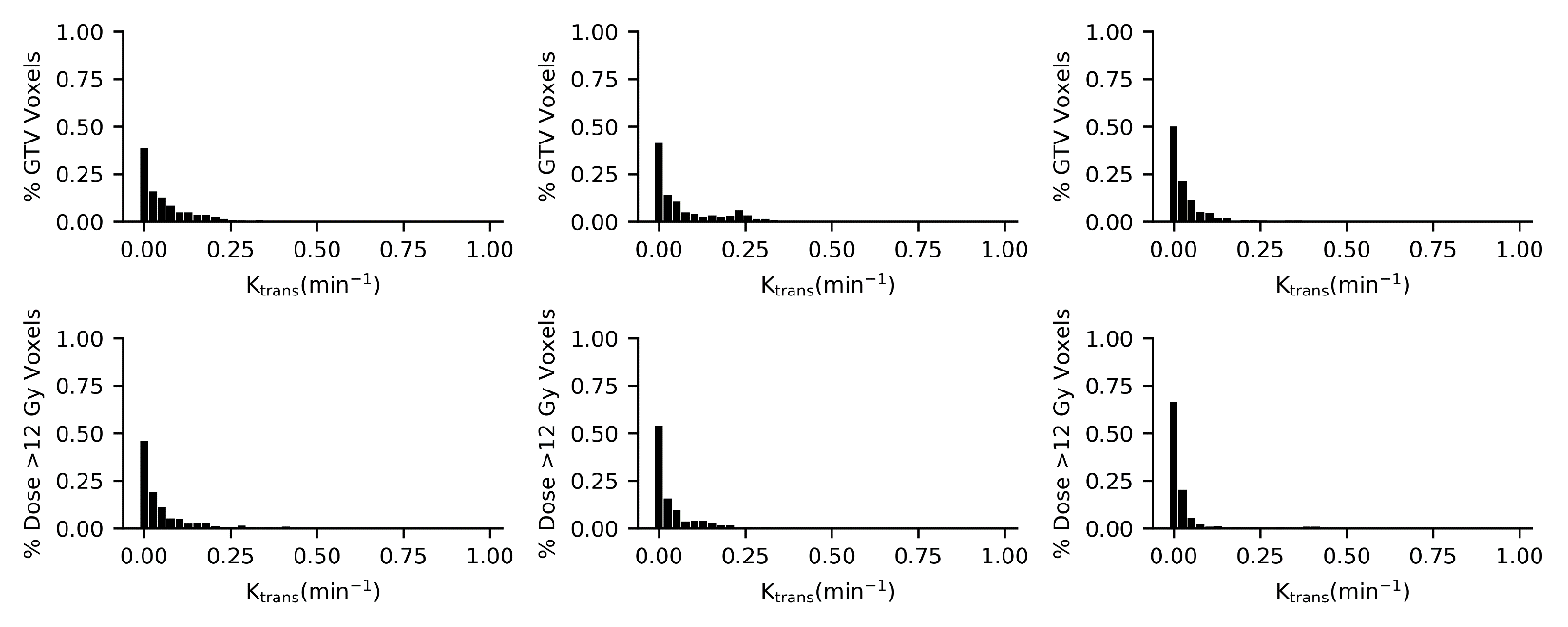


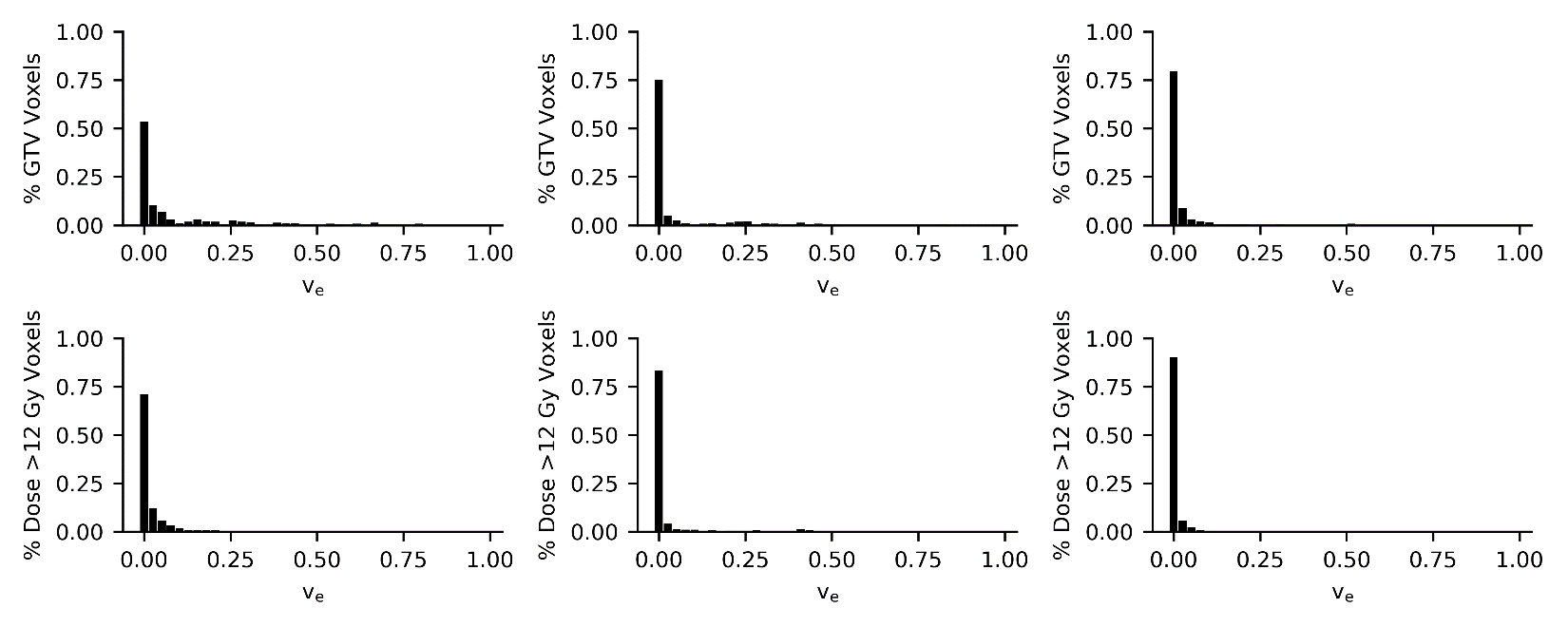


**Day 0 Day 3 Day 20**

**Patient 17: Metastasis 1
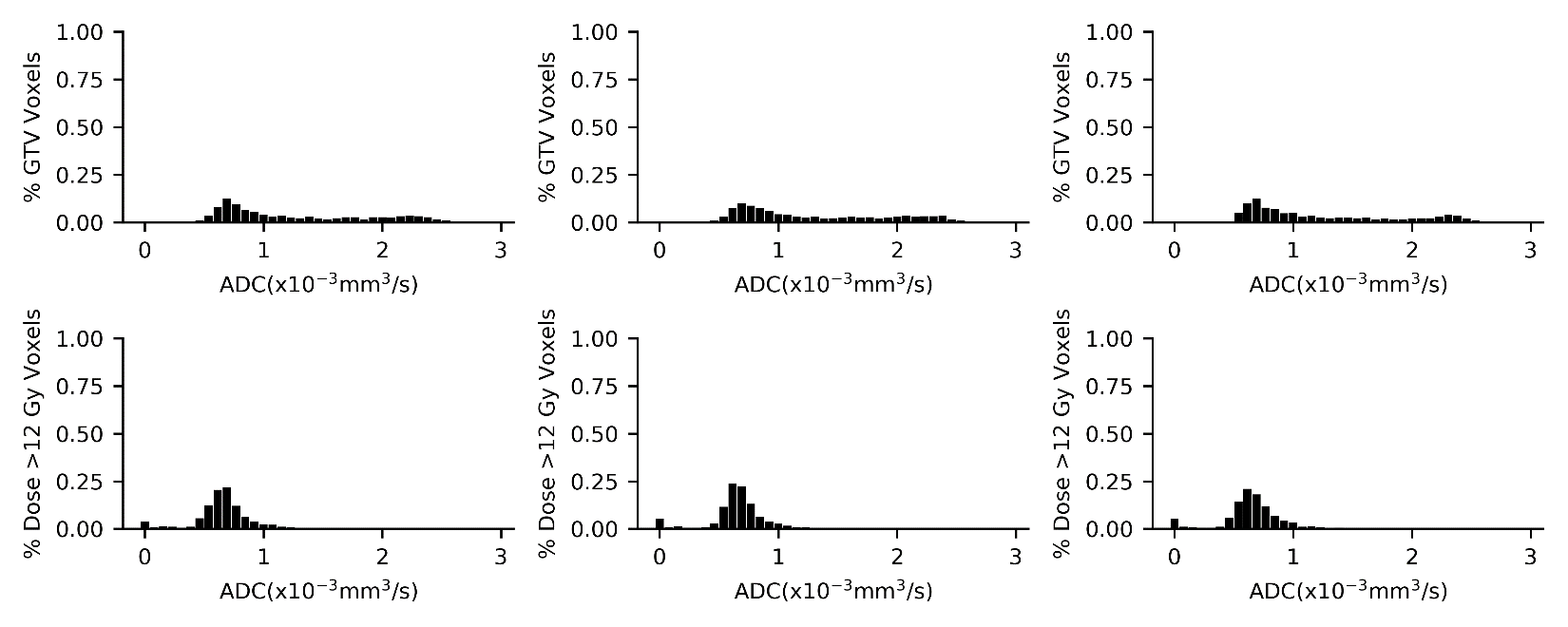
**

**
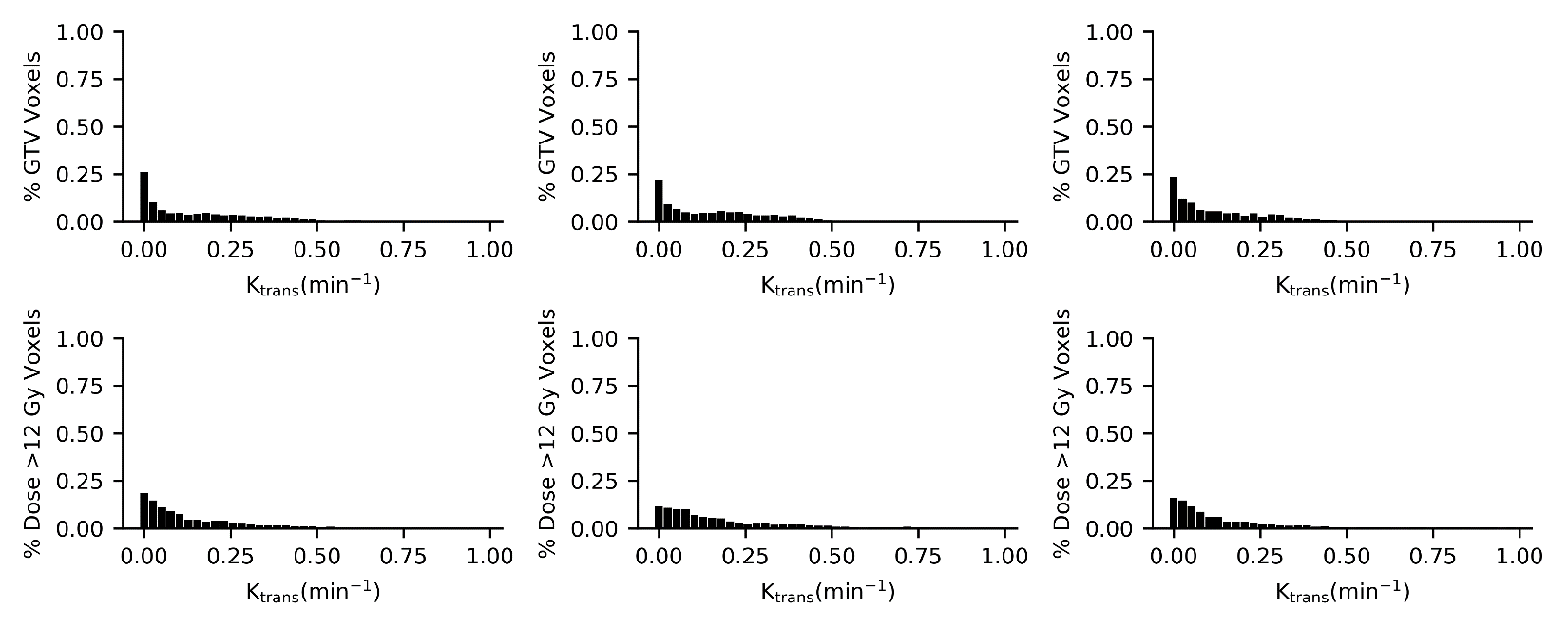
**

**
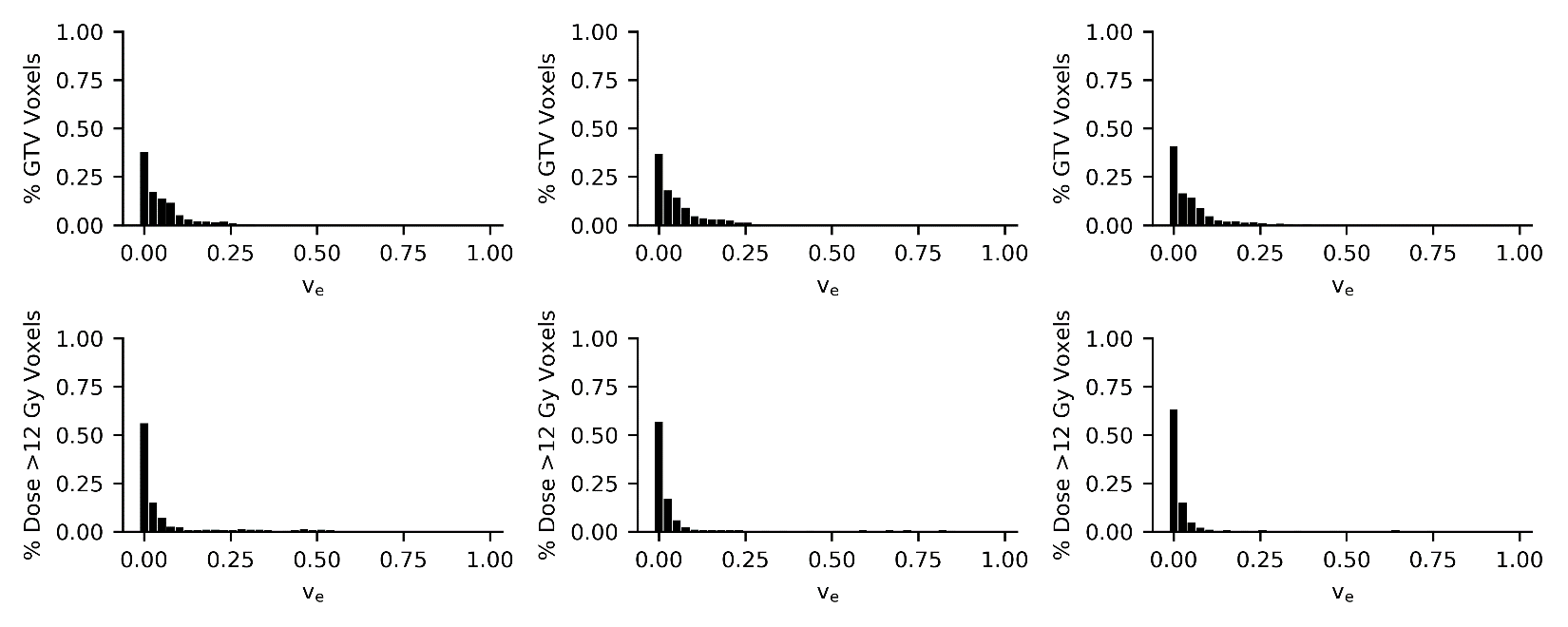
**

**Day 0 Day 3 Day 20**

**Patient 17: Metastasis 2
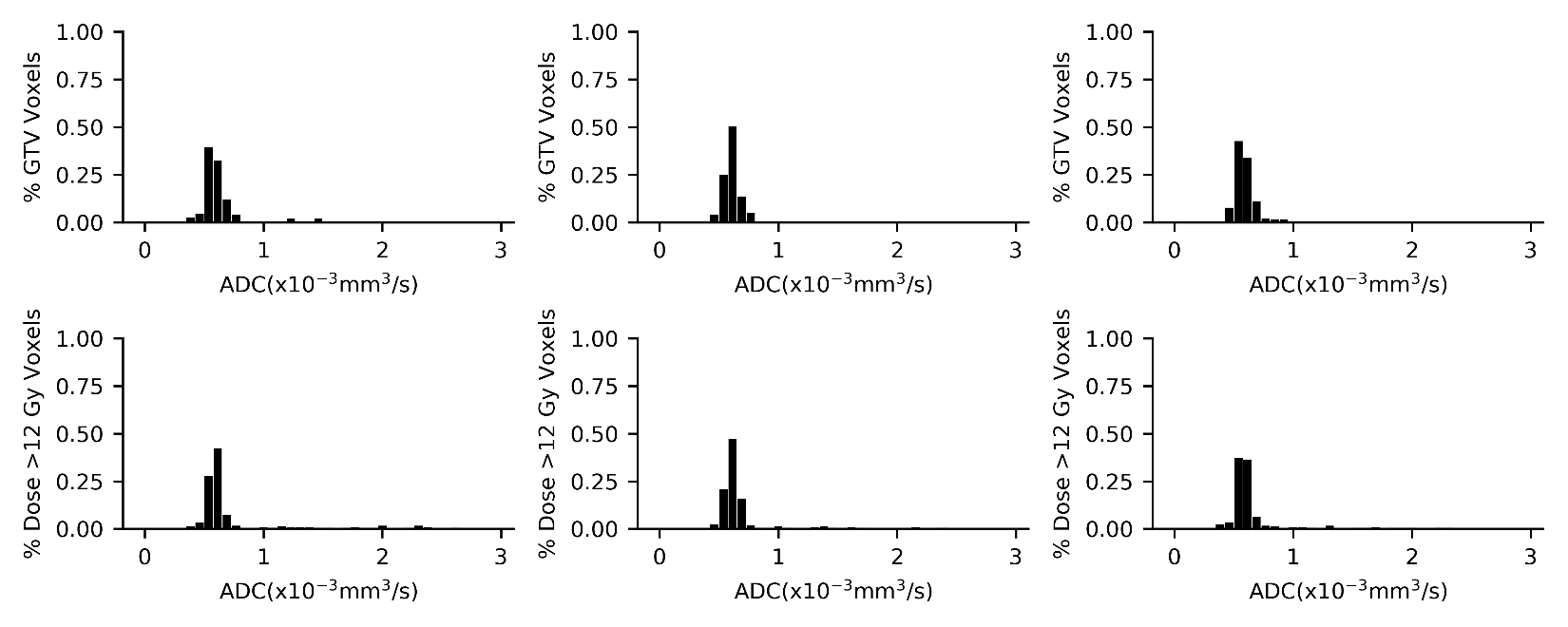
**

**
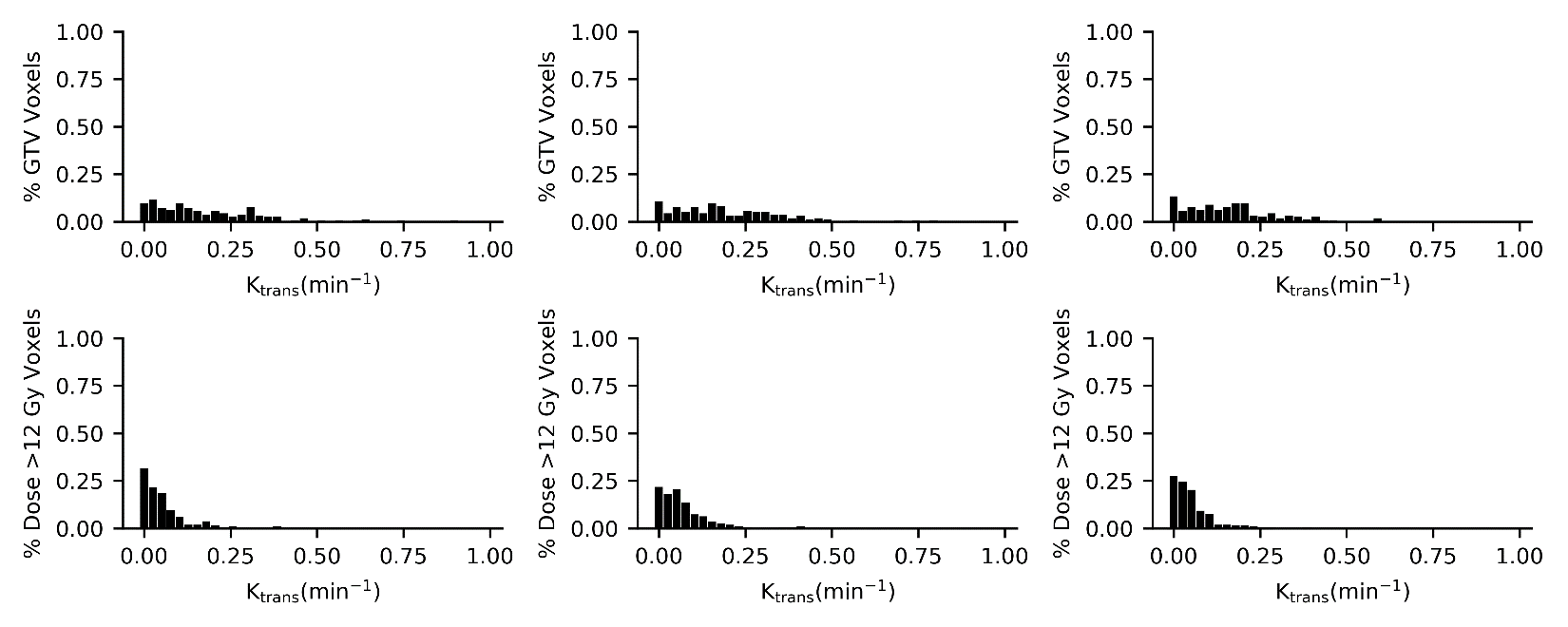
**

**
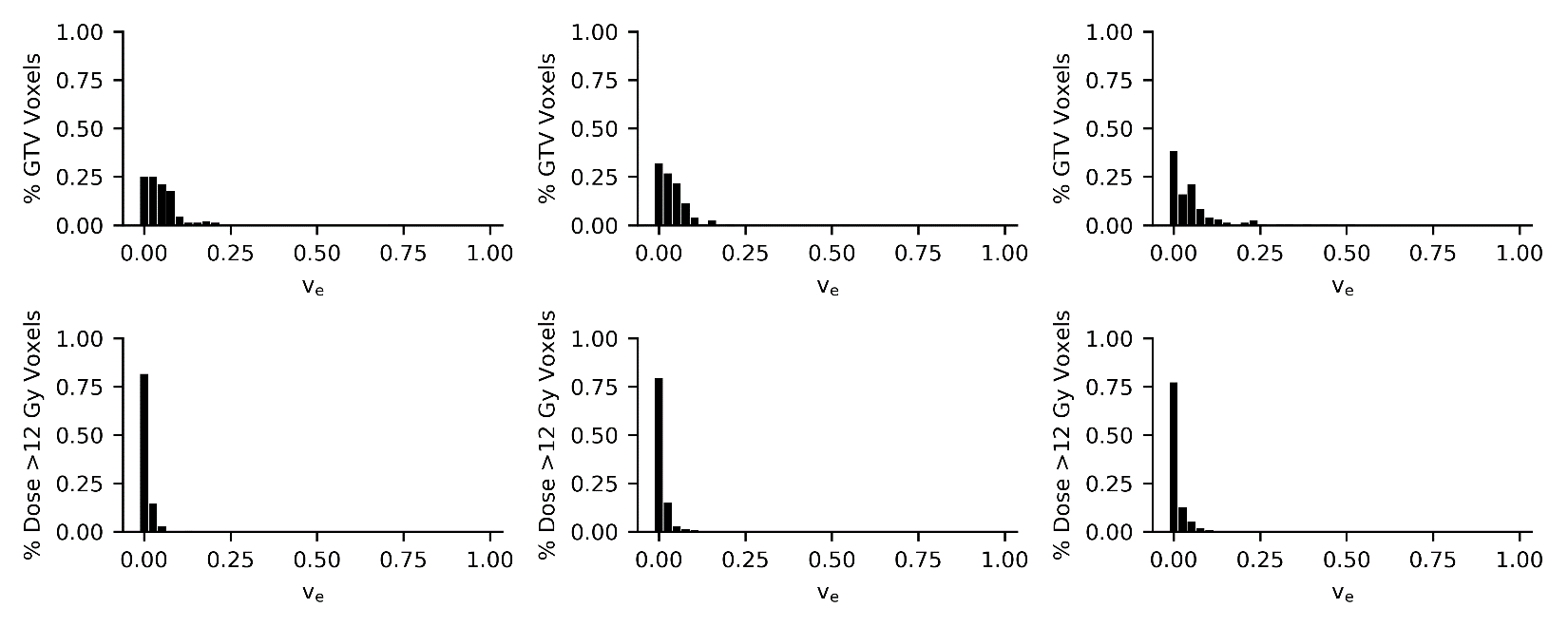
**

**Day 0 Day 3 Day 20**

**Patient 18: Metastasis 1
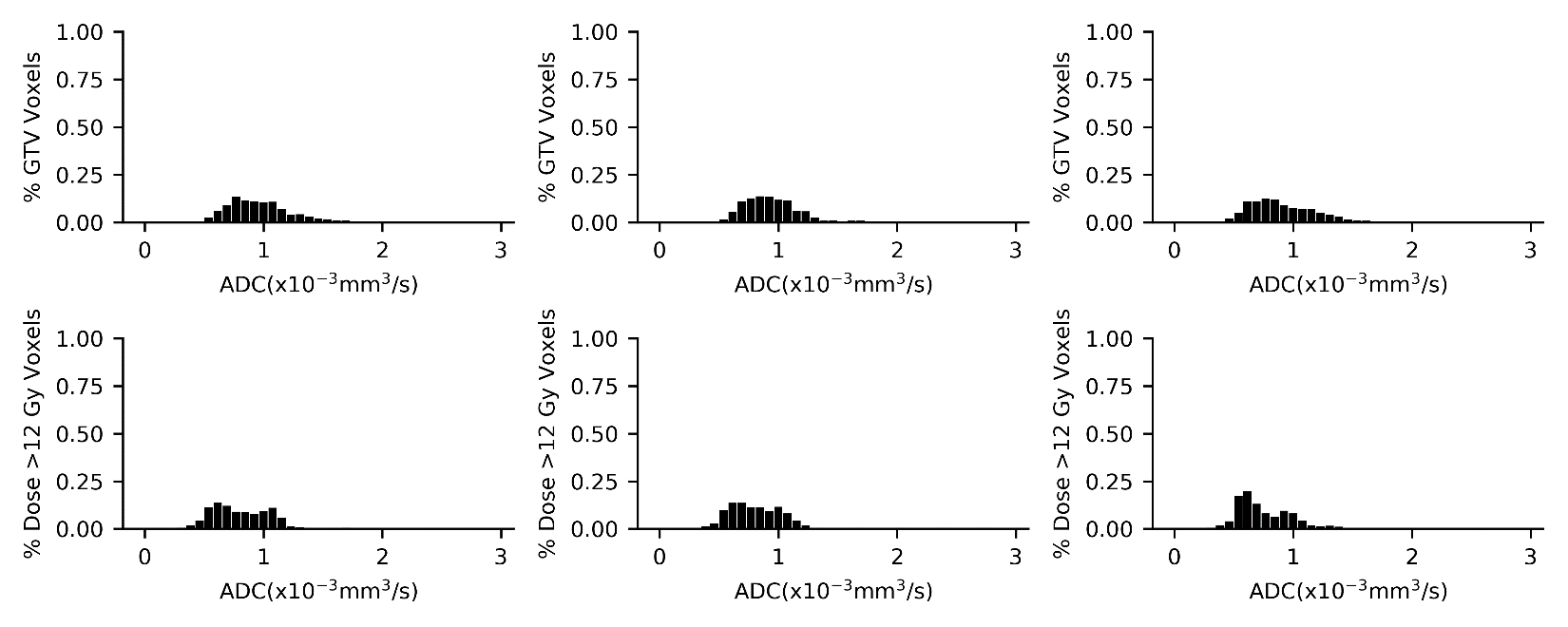
**


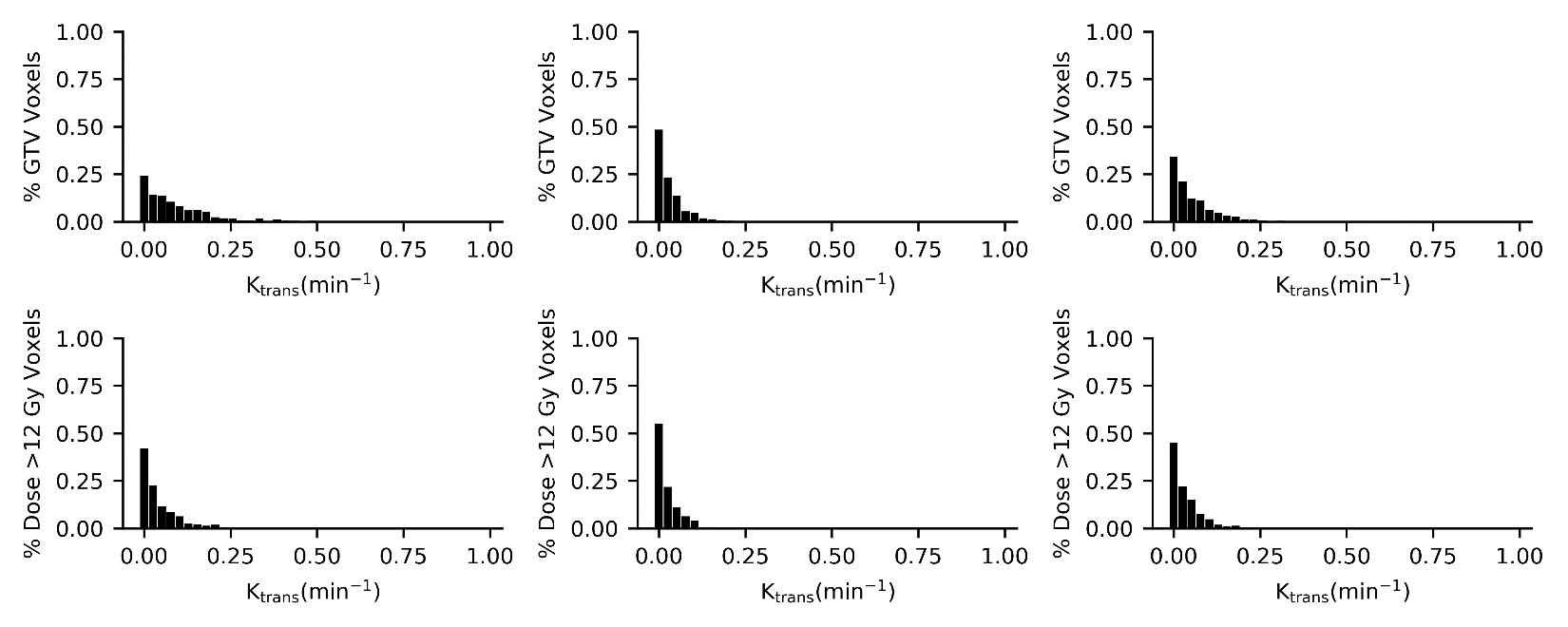


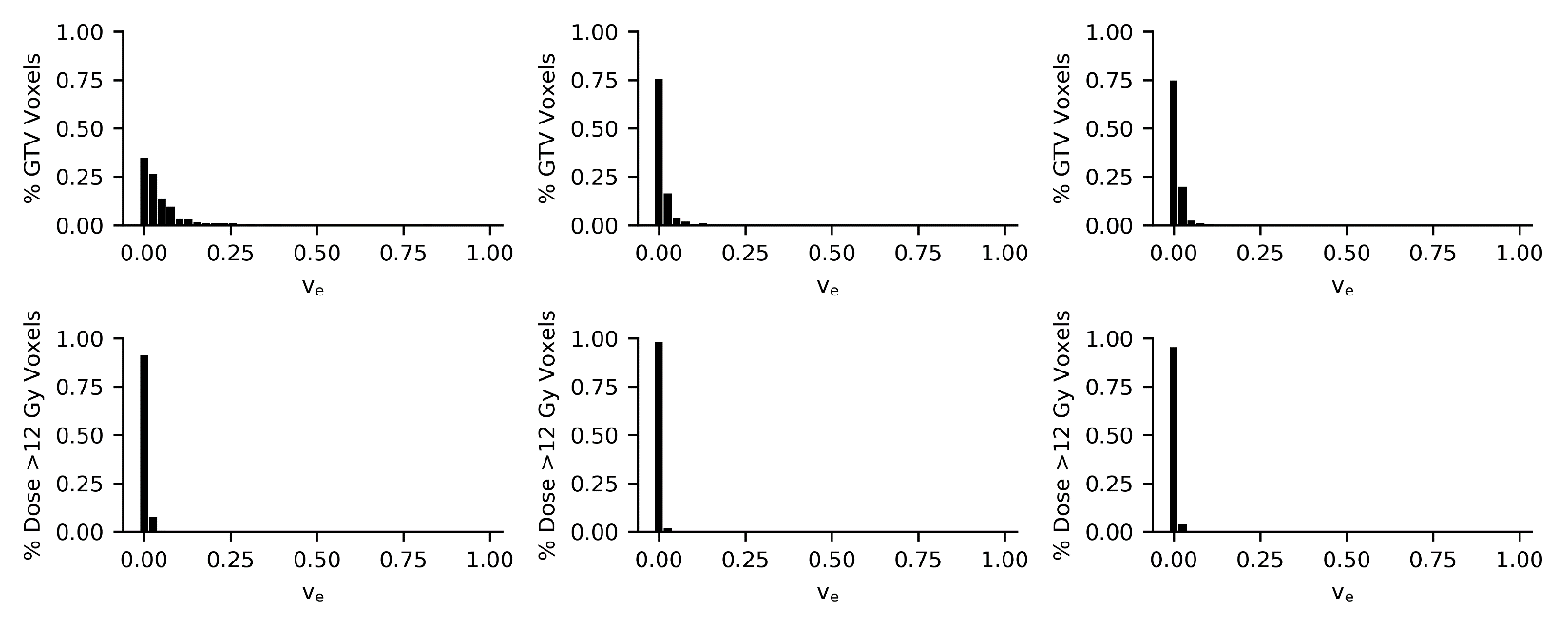


**Day 0 Day 3 Day 20**
