# Supplementary figures and images for: Detectability of radiation-induced changes in magnetic resonance biomarkers following stereotactic radiosurgery: A pilot study
Source: PLoS One. 2018 Nov 26;13(11):e0207933. doi: 10.1371/journal.pone.0207933 (PMC6258119; doi:10.1371/journal.pone.0207933)

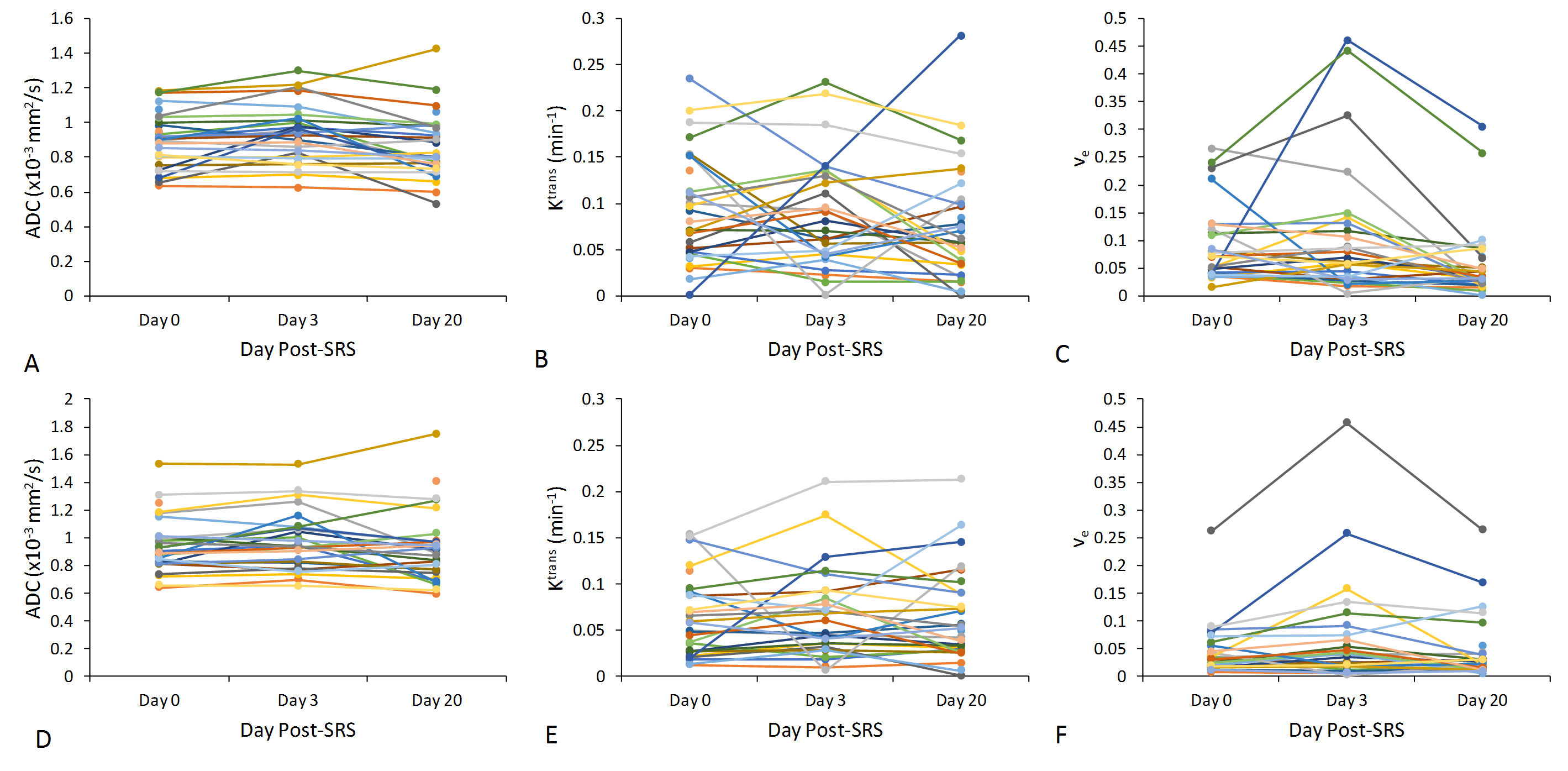

Supplement: S2 Fig — Mean ADC, Ktrans and ve value changes per metastasis at each time point for all patients in the GTV (A—C) and non-target region > 12 Gy (D–F). (TIF) [file pone.0207933.s002.tif]
